# Radiotherapy boost to the primary tumour in locally advanced rectal cancer: Systematic review of practices and *meta*-analysis

**DOI:** 10.1016/j.ctro.2025.101014

**Published:** 2025-07-13

**Authors:** Julien Pierrard, Lorraine Donnay, Alix Collard, Geneviève Van Ooteghem

**Affiliations:** a Institut de Recherche Expérimentale et Clinique (IREC), Center of Molecular Imaging, Radiotherapy and Oncology (MIRO), Université Catholique de Louvain, Brussels, Belgium; b Department of Radiation Oncology, Cliniques Universitaires Saint-Luc, Brussels, Belgium; c Department of Radiation Oncology, CHU UCL Namur, Namur, Belgium; d Statistical Support Unit, King Albert II Cancer Institute, Cliniques universitaires Saint-Luc, Brussels, Belgium

**Keywords:** Locally advanced rectal cancer, Meta-analysis, Rectal boost, Dose escalation, Neo-adjuvant therapy, Complete response

## Abstract

**Introduction:**

In locally advanced rectal cancer (LARC), increasing the complete response (CR) rate after total neo-adjuvant treatment may increase the patient eligibility for non-operative management (“watch and wait”, W&W). Although a radiotherapy (RT) boost to the primary tumour may enhance CR rates, clear guidelines are currently lacking. This systematic review and *meta*-analysis investigate the technical parameters used for rectal boost RT and assess their impact on oncological outcomes.

**Methods:**

Following PRISMA guidelines, the terms “rectum,” “radiotherapy,” and “boost” were searched in PubMed and EMBASE (PROSPERO: CRD42023444685). Studies reporting on external beam RT boost to the primary tumour in LARC and meeting quality criteria were included. Descriptive analyses extracted data on RT technique, preparation, boost delineation, dose, chemotherapy, and follow-up. A mixed-effects *meta*-analysis model evaluated the impact of selected parameters on CR and local recurrence rate (LRR). Studies were analysed separately based on treatment intent: planned surgery or W&W.

**Results:**

Out of 3904 references, 83 were included in the descriptive analysis and 78 in the *meta*-analysis. Substantial variability in RT parameters was observed across studies. Pathologic CR rates were significantly higher with intensity-modulated/volumetric-modulated arc RT (IMRT/VMAT, p = 0.007), simultaneous boost (p = 0.020), dose escalation (Biological equivalent dose > 74 Gy, p = 0.035), and the combination of induction and consolidation chemotherapy (p = 0.023). No significant associations were found for clinical CR or LRR.

**Conclusion:**

While rectal RT boost is already part of real-world practices, the wide heterogeneity in techniques highlights the urgent need for standardisation. Our *meta*-analysis suggests that IMRT/VMAT, simultaneous boost, and dose escalation are associated with higher pathological CR rates and should be considered in future rectal boost guidelines. These findings, however, warrant careful interpretation due to the absence of adjustments for clinical, tumoral, or patient-related parameters that may also influence response rate and oncological outcomes.

## Highlights

• Primary tumour RT boost is common practice in locally advanced rectal cancer.
• High variability exists in techniques used for external radiotherapy rectal boost.
• IMRT/VMAT, simultaneous boost, and dose escalation improve pathological CR.
• No RT parameters are associated with clinical CR and LRR, while data are limited.
• Rectal boost radiotherapy guidelines are urgently required to standardise practice.

## Introduction

Rectal cancer is the 8th most common malignancy worldwide and accounts for approximatively 344 000 deaths annually [1]. In locally advanced rectal cancer (LARC, defined as cT3-cT4 or N + M0 disease), Total Neo-adjuvant Treatment (TNT) has emerged as a treatment option for selected patients, before surgical resection [2]. TNT consists of a preoperative combination of induction or consolidation chemotherapy with radio(chemo)therapy [[2], [3], [4]]. In recent years, the “watch and wait” (W&W) strategy after TNT has also emerged as an interesting approach. This W&W strategy offers a non-operative management with active surveillance for patients achieving a clinical complete response (cCR) after TNT, with the aim of preserving the rectum and improve quality of life [[5], [6], [7]]. During radiotherapy (RT), dose escalation to the primary tumour, or “boost”, can be delivered via external-beam RT (EBRT) or contact x-ray brachytherapy [2,5,6,8]. This boost has shown the potential to improve complete response (CR) rates [9,10]. However, its benefit, particularly for EBRT boost, has not been confirmed in prospective trials and may be ironically associated with a transient decline in quality of life [8,11]. Further investigation is therefore warranted. While international recommendations exist for pelvic lymph node RT in LARC, no consensus is currently available for the primary rectal boost [12]. Before such guidelines can be established, a comprehensive understanding of the different techniques used for this RT boost and their impact is essential.

The first objective of this systematic review is to provide a descriptive analysis of the technical approaches used for RT boost planning across institutions. The second objective is to evaluate, through *meta*-analysis, the impact of these technical parameters on CR rates and local recurrence rates (LRR). Overall, this work aims to provide the foundation for future consensus guidelines on rectal boost RT.

## Methods

This systematic review and *meta*-analysis were conducted in accordance with the Preferred Reporting Items for Systematic reviews and Meta-Analyses (PRISMA) guidelines [13]. The protocol was published in the PROSPERO database (CRD42023444685).

### Search strategy

MEDLINE and Embase were searched using different combinations of the terms “rectum”, “radiotherapy”, “radiation therapy”, “boost”, and “dose escalation”. The initial search covered studies published from January 2000 to May 2023. The search was relaunched to include publications until June 14, 2024. Duplicate records were removed.

### Studies selection

Titles and abstracts were screened independently by two operators (J.P. and G.V.O.) using the PICOS (population, intervention, control, outcome, study design) approach [14]. Studies were eligible for full-text screening if they involve (P) human patients with rectal adenocarcinoma treated with (IC) a neo-adjuvant RT rectal boost/dose escalation in the primary setting. Analysed outcomes (O) are described in the next section. Retrospective, prospective, *in silico* studies, and study protocols (S) were included. Exclusion criteria included non-human studies, other cancers, intraoperative or postoperative boosts, non-English/French language, and inaccessible full texts. Conflicts were resolved by consensus. Full-text screening was conducted by one operator (J.P.) using the same eligibility criteria. Additionally, studies using only brachytherapy or contact X-ray therapy for the boost, as well as conference abstracts, were excluded. Reference lists of review articles were screened to identify any relevant studies not captured by the initial search. Aggregate data were extracted by J.P., using only publicly available information; no attempt was made to contact authors for missing data.

### Boost descriptive analysis

The descriptive analysis collected data on publication details, RT technique, delivery modalities, patient preparation, boost delineation, dose, concomitant chemotherapy, neo-adjuvant chemotherapy (induction and/or consolidation), and treatment strategy. Due to anticipated interstudy heterogeneity, data were categorised (Supplementary Table 1). RT doses were converted into biologically effective dose (BED) using an alpha/Beta ratio of 5.06 (Appendix A) [15].

When multiple studies from the same institution were identified, only the most recent was retained to better reflect current clinical practices.

### Meta-analysis

The primary endpoint was the CR rate across subgroups defined in the descriptive analysis, including publication year, RT technique, clinical target volume (CTV) definition, planning target volume (PTV) margin, boost sequence, boost BED, concomitant chemotherapy, neo-adjuvant chemotherapy, and surgical delay. Studies with planned surgery reporting pathological complete response (pCR) rate were analysed separately from W&W studies reporting clinical complete response (cCR). A tumour regression grade (TRG) 1 was considered equivalent to a pCR [16]. Given the expected limited number of eligible W&W cohorts, the corresponding analysis is presented separately (Appendix B).

The secondary endpoint was the local recurrence rate (LRR), within the same subgroups as CR analysis and based on the time-to-event data available in each study. In planned surgery cohorts, LRR was defined as the number of pelvic recurrences among all included patients. In W&W studies, LRR included pelvic recurrences in both patients undergoing immediate surgery for non-cCR and those managed non-operatively after achieving an initial cCR.

Only studies consistently reporting or enabling the direct calculation of intention-to-treat results were included. When a single study reported multiple independent cohorts, based on differences in analysed factors, each cohort was analysed separately, provided CR outcomes were reported individually.

### Quality assessment

A modified Newcastle-Ottawa Scale adapted to this study purpose was used for quality evaluation [17]. Eight items were scored by one operator (J.P.) from “A” (high quality) to “C” (poor quality, Supplementary Table 2). For the descriptive analysis, studies were excluded if one of the two first items (“Complete RT technique description” and “Correct terminology”) scored “C”. For the *meta*-analysis, exclusion applied if any item scored “C”.

### Statistical analysis

Wilcoxon signed-rank tests were used for comparisons of ordered variables. For the *meta*-analysis, pooled estimates of CR and LRR were presented as forest plots with 95 % confidence interval, using the Knapp-Hartung adjustment [18]. The between-study heterogeneity variance (τ^2^) was evaluated using the maximum likelihood estimator [19,20]. For subgroup containing fewer than five studies, a pooled τ^2^ was calculated [21]. Statistical heterogeneity was quantified using the I^2^ statistic [22]. Subgroup comparisons were performed using a mixed-effects model using the Q-test, without adjustment for confounding factors. Given the study objective of identifying parameters associated with CR and LRR, only subgroup *meta*-analyses were reported, and no overall pooled estimates were provided.

A *meta*-regression model was conducted to explore the relationship between the RT boost dose and pCR rate (Appendix C).

Publication bias was assessed by visual inspection of funnel plots and Peters’ tests [23,24].

For all statistical analyses, a p-value < 0.05 was considered significant. All analyses were performed in RStudio (R version 4.2.1.) using the “meta” (v7.0–0), “dmetar” (v0.1.0), and “nls2” (v0.3–4) packages [25].

## Results

### Descriptive analysis

**Fig. 1:**
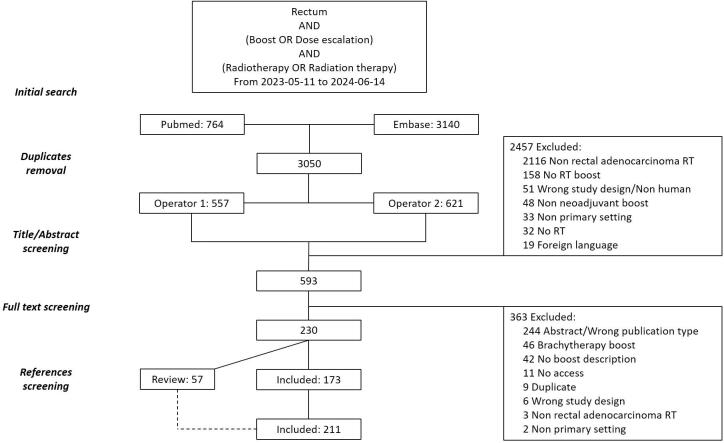
Flow chart of study selection. (Should NOT be printed in colours). RT: Radiotherapy.

The final search (June 14, 2024) identified 3904 references. After duplicate removal, 3,050 unique records remained. Of these, 2378 were excluded by both operators, with consensus achieved for 110 studies. A total of 593 references were selected for full-text assessment. After including references identified from reviews, 211 full papers were included in the systematic review (Fig. 1, Supplementary Figs. 1 and 2). Four studies reported two different RT boost dose levels; only the highest was considered for analysis [[26], [27], [28], [29]].

After institutional duplicate removal, 115 studies were included for quality assessment. Among these, 22 and 29 studies were rated “C” for the quality criteria “Complete RT description” and “Correct terminology”, respectively; 19 of them received a “C” for both criteria (Supplementary Fig. 3). Ultimately, 83 studies including 8060 patients were included in the descriptive analysis (Supplementary Tables 3 and 4) [5,6,[26], [27], [28], [29], [30], [31], [32], [33], [34], [35], [36], [37], [38], [39], [40], [41], [42], [43], [44], [45], [46], [47], [48], [49], [50], [51], [52], [53], [54], [55], [56], [57], [58], [59], [60], [61], [62], [63], [64], [65], [66], [67], [68], [69], [70], [71], [72], [73], [74], [75], [76], [77], [78], [79], [80], [81], [82], [83], [84], [85], [86], [87], [88], [89], [90], [91], [92], [93], [94], [95], [96], [97], [98], [99], [100], [101], [102], [103], [104], [105]]. There were 39 prospective, 23 retrospective, 18 in-silico studies, and 3 protocols included (Supplementary Table 3). Detailed references are reported in Supplementary Table 3 for clarity.

#### RT technique: IMRT/VMAT more frequently used than 3D in recent studies

Three-dimensional (3D) RT was used exclusively in 28.9 % (24/83) of studies, while intensity-modulated RT (IMRT)/volumetric-modulated arc RT (VMAT) was used in 38.6 % (32/83). The median publication year was significantly lower for studies using 3D compared to those using IMRT/VMAT (2012 vs. 2019, p < 0.001). Magnetic resonance imaging (MRI)-guided RT was used by 4.8 % (4/83) of teams. IMRT/VMAT and 3D were both used in 16.9 % (14/83) of studies. Proton therapy was investigated in 2.4 % (2/83) of studies. The RT technique was not reported in 9.6 % (8/83) of studies.

#### RT boost delivery modalities: sequential and simultaneous boosts are equally common; adaptive RT is emerging

The boost was delivered sequentially in 42.2 % (32/83) and simultaneously in another 42.2 % (32/83) of publications, with no significant difference in the median publication year (2016 vs. 2019, p = 0.09). Both approaches were used in 9.6 % (8/83), and 3.6 % (3/83) described mixed sequential/simultaneous boosts. The delivery modality was not specified in 2.4 % (2/83) of studies. Four teams delivered the boost during a second daily session on selected days [58,60,91,106]; one used bifractionated sequential boost [79], and another reported bifractionated RT for the entire treatment course [78]. Since 2018, adaptive RT for rectal boost delivery has been reported by nine teams [29,42,53,59,66,72,73,94,101].

#### RT preparation and setup: bladder filling protocols and supine positioning are advised, but high variability remains

**Fig. 2:**
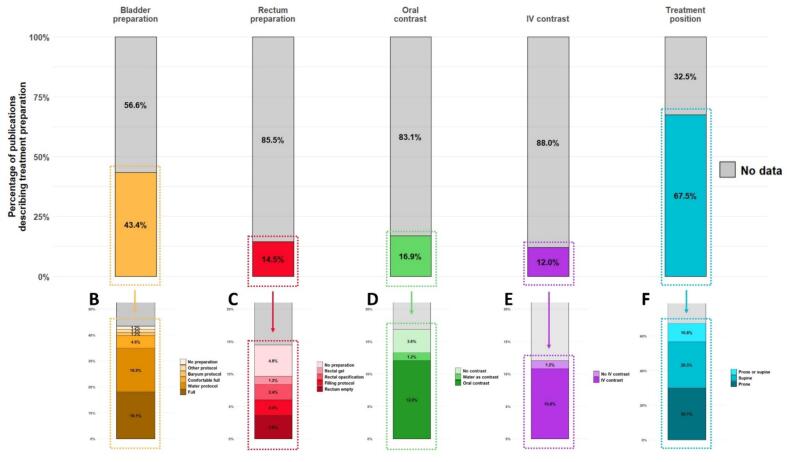
Radiotherapy treatment preparation parameters (A) across included studies (n = 83). (B) Details of bladder preparation in 36/83 studies, (C) rectum preparation in 12/83 studies, (D) oral preparation in 11/83 studies, (E) IV contrast use in 10/83 studies, and (F) treatment position in 56/83 studies reporting the corresponding parameter. (Should NOT be printed in colours). IV: Intravenous.

Treatment positioning and bladder preparation were the most frequently reported parameters, mentioned in 67.5 % (56/83) and 43.4 % (36/83) of studies, respectively. Details on oral or rectal preparation and the use of intravenous contrast for planning CT were less commonly reported (Fig. 2A).

Bladder protocols were multiple but the most frequent approaches involved a full bladder (18.1 %, 16/83) or water intake (16.9 %, 15/83, Fig. 2B).

Rectal preparations, reported in 14.5 % (12/83), also varied widely: rectal emptying [30,50,57], filling [68,86], opacification [39,89], gel use [42], or no specific preparation in 4 studies [33,53,101,103] (Fig. 2C).

Oral contrast administration for planning CT was mentioned in 13.3 % (11/83) of studies, with one specifying water as the contrast agent [34]; three studies explicitly reported not using oral contrast (Fig. 2D) [53,100,103].

Intravenous contrast for planning CT acquisition was mentioned in 10.8 % (9/83) of studies; one explicitly stating its absence [27] (Fig. 2E).

When studies reported the treatment position, 30.1 % (25/83) used the prone position and 26.5 % (22/83) used supine. Supine positioning was more common in recent publications (median publication year 2020 vs. 2016 for prone, p < 0.001, Fig. 2F).

Other rare preparation techniques included use anal radiopaque marker during planning CT acquisition [81,84,90], pre-rectal hydrogel injection [30], and fasting [98].

#### RT boost volumes: marked heterogeneity across studies, from tumour-only to inclusion of total mesorectum and presacral space

As with RT preparation, substantial inter-study variability was observed in the boost volume definition. In 10.8 % (9/83) of studies, the boost target was defined merely as the “primary tumour” with no further details, making it impossible to distinguish between gross tumour volume (GTV), CTV, and PTV per ICRU standards [107]. These studies were excluded from the subsequent analysis.

**Fig. 3:**
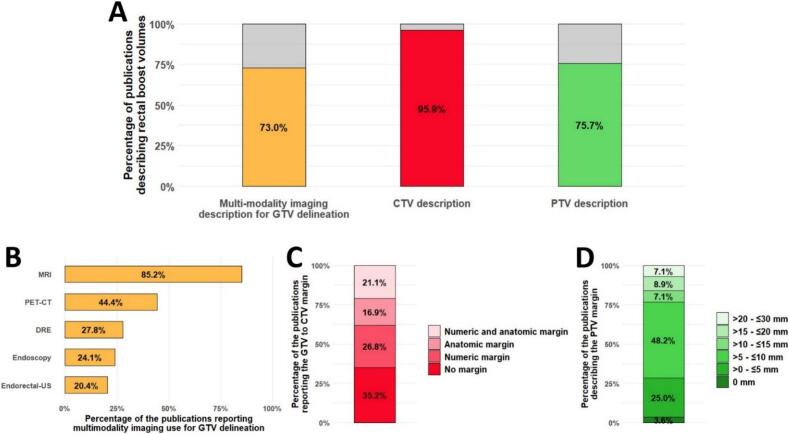
RT boost volume definitions and delineation methods. (A) Proportion of studies reporting the GTV, CTV and PTV description among the 74 studies describing their boost volumes. (B) Multi-modality imaging use in publications describing GTV delineation (54/74 studies). (C) GTV to CTV margin strategies among the studies reporting CTV definition (71/74). (D) PTV margin sizes among publications describing PTV boost (56/74). If margins were anisotropic, the largest value was reported. Two studies additionally incorporated anatomical boundaries alongside numeric margins. (Should NOT be printed in colours). CTV: Clinical target volume, DRE: Digital rectal examination, GTV: Gross tumour volume, MRI: Magnetic resonance imaging, PET-CT: Positron emission tomography − computed tomography, PTV: Planning target volume, US: Ultrasonography.

Among the remaining studies, 73.0 % (54/74) reported using multimodality imaging (Fig. 3A). The imaging and clinical modalities employed for GTV delineation were, in descending order, MRI (85.2 %, 46/54), positron emission tomography (PET-CT, 44.4 %, 24/54), digital rectal examination (27.8 %, 15/54), endoscopy (colonoscopy/rectoscopy, 24.1 %, 13/54), and endorectal ultrasonography (20.4 %, 11/54, Fig. 3B).

CTV definition was missing in 4.1 % (3/74) of publications. When described, 35.2 % (25/71) applied no margin to the GTV, 26.8 % (19/71) used numerical margins, 16.9 % (12/71) applied purely anatomical margins, and 21.1 % (15/71) combined both approaches. Numerical margins (including publications also using anatomic margins) were ≤10 mm in 8 studies [30,37,38,43,50,66,83,86], >10 and ≤20 mm in 18 studies [27,32,33,44,48,49,55,56,58,59,63,64,67,[79], [80], [81],^87^,^108^], and >20 mm in 4 studies [31,60,78,109]; four used anisotropic numerical margins [69,70,72,84]. Anatomic margins included: circumferential involved rectum wall in 5 papers [5,[33], [34], [35],^90^], involved mesorectum in 15 papers [31,32,37,48,49,59,61,63,71,77,82,86,96,105,109], entire presacral space in 2 papers [67,78], or other anatomical definitions in 6 papers [66,68,69,72,97] (Fig. 3C).

Empirically defined internal target volume (ITV) margins were reported by 3 teams [34,37,43].

PTV margins were described in 75.7 % (56/74) of publications, and among them, 80.4 % (45/56) used isotropic expansion (Fig. 3D). Two teams incorporated anatomic boundaries into their PTV definition [5,90].

#### RT dose: Rectal boost BED ranges from 56.9 to 103.2 Gy

All doses prescriptions were converted into BED. Equivalencies for common clinical dose are provided in Appendix A, Table A1.

**Fig. 4:**
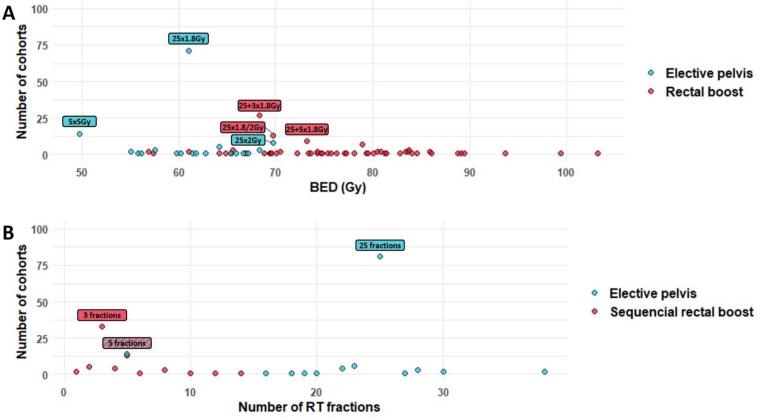
(A) Prescribed RT dose in the 117 different cohorts identified from the 83 included publications, for both the elective pelvic volume and the rectal boost volume. (B) Number of fractions delivered to the elective pelvic volume and the boost volume when given sequentially. (Should NOT be printed in colours). BED: Biologically effective dose, RT: Radiotherapy.

Across the 83 included publications, 117 distinct cohorts were identified based on dose prescription. The median BED for the elective pelvic volume was 61.0 Gy (range: 49.7–69.8 Gy), while the median BED for the boost volume was 70.5 Gy (range: 56.9–103.2 Gy, Fig. 4A). The median number of fractions was 25 (range: 5–38) for the elective pelvic volume and 3 (range: 1–14) for sequential boosts (Fig. 4B).

#### Chemotherapy: concomitant fluoropyrimidine is standard; induction and/or consolidation chemotherapy is still not frequent

**Fig. 5:**
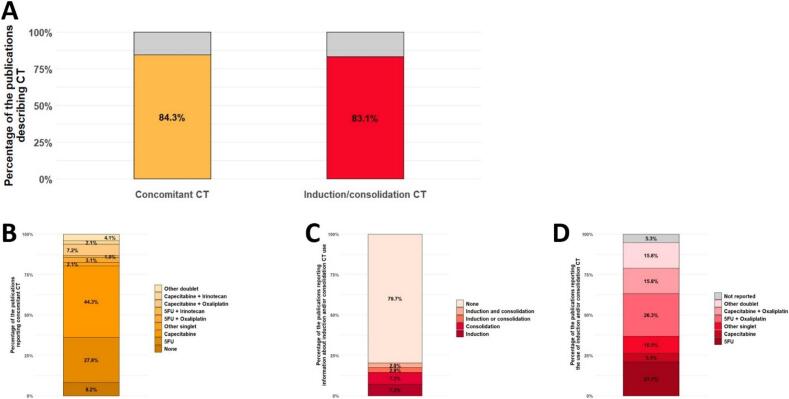
(A) Proportion of studies reporting data on concomitant and on induction and/or consolidation chemotherapy. (B) Distribution of concomitant chemotherapy regimens across the 97 cohorts identified among the studies that reported this information (70/83). (C) Chemotherapy sequencing (induction and/or consolidation) in the studies reporting such data (69/83). (D) Regimens used in the 19 cohorts reporting induction and/or consolidation chemotherapy. (Should NOT be printed in colours). 5FU: 5-fluorouracile, CT: Chemotherapy.

Concomitant chemotherapy was not reported in 15.7 % (13/83) of studies (Fig. 5A). Among the remaining 70 studies, multiple regimens were described, resulting in 97 different cohorts. Of these: 8.2 % (8/97) received no chemotherapy, 74.2 % (72/97) received single-agent, and 17.5 % (17/97) received doublet. The most frequently used single-agents were capecitabine (44.3 %, 43/97), and 5-fluorouracil (27.8 %, 27/97). The most common doublet was capecitabine + oxaliplatin (7.2 %, 7/97, Fig. 5B). Less frequent regimens included raltitrexed [66], UFT [34], capecitabine + anakinra [54], capecitabine + bevacizumab [56], irinotecan + s-1 [71], and UFT + oxaliplatin [95].

Data on induction and/or consolidation chemotherapy were unavailable in 16.9 % (14/83, Fig. 5A) of studies. Among the studies that did report this information: 79.7 % (55/69) used no additional systemic treatment, 7.2 % (5/69) reported induction chemotherapy [26,53,56,81,91], 7.2 % (5/69) reported consolidation chemotherapy [37,73,101,102,108], 2.9 % (2/69) reported either induction or consolidation chemotherapy [5,46], and 2.9 % (2/69) administered both induction and consolidation chemotherapy [74,95] (Fig. 5C). Among the 19 reported induction and/or consolidation regimens: 36.8 % were single-agent (mostly 5-FU [37,74,81,108]), and 57.9 % were doublets, typically fluoropyrimidine + oxaliplatin [5,26,73,74,81,91,102] (Fig. 5D). The regimen was not described in 1 study [46]. The use of induction and/or consolidation chemotherapy appears to be a relatively recent development, as reflected by a median publication year of 2019 for studies reporting such regimens. Only five studies implementing these strategies were published before 2017 [26,56,81,95,108].

#### Treatment strategy: the W&W approach is increasingly adopted

The post-RT treatment strategy was planned surgery in 63.9 % (53/83) and W&W in 15.7 % (13/83) publications, while 20.5 % (17/83) did not report their approach. The median publication year was 2022 for W&W vs. 2017 for planned surgery (p < 0.001).

Among the planned surgery publications, surgical delay was < 9 weeks in 43.4 % (23/53), >9 weeks in 7.5 % (4/53), overlapping both categories in 41.5 % (22/53), and not reported in 7.5 % (4/53).

In W&W publications, clinical response assessment involved MRI in 76.9 % (10/13), endoscopy in 69.2 % (9/13), digital rectal examination in 46.2 % (6/13), CT in 23.1 % (3/13), PET-CT in 7.7 % (1/13) [69], and endorectal ultrasound in 7.7 % (1/13). No information was provided in 2 publications [35,53]. The timing of cCR assessment ranged from 6 to 24 weeks. It was not specified in 3 publications [53,69,76] (Supplementary Table 3). The frequency of follow-up evaluations was missing in 5 publications [29,35,53,76,83]. This frequency differed among teams, but generally followed a schedule of every 3 months for 2 years, then every 6–12 months for the subsequent 3 years [5,6,34,42,54,55,69,94].

### Meta-analysis results

Of the 141 initially eligible studies (including 212 different cohorts), 63 were excluded following quality assessment (Supplementary Table 5 and Supplementary Fig. 4), leaving 78 publications with 97 analysable cohorts. The most frequent reasons for exclusion were scoring “C” for “Correct terminology” (30.7 %, 65/212) and “Complete RT description” (20.8 %, 44/212) criteria. Conversely, “Cohort selection” and “Outcome assessment” were rated “A” in 99.5 % (211/212) and 88.2 % (187/212) of cases, respectively, reflecting a good confidence in the *meta*-analysis results based on these criteria. Among the 97 retained cohorts, 87 reported pathological complete response (pCR) rates (n = 7125 patients, Supplementary Table 6), and 10 reported clinical complete response (cCR) under a W&W strategy (n = 927 patients, Supplementary Table 7).

**Fig. 6:**
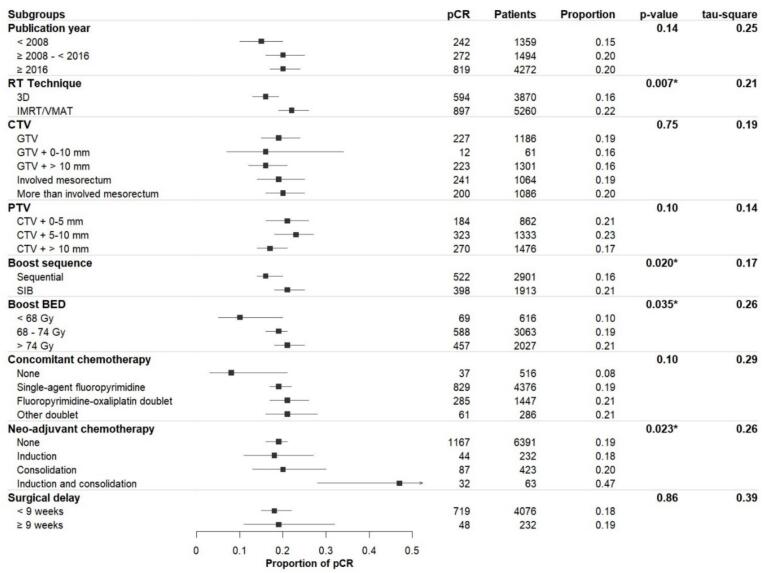
Subgroup *meta*-analysis of planned surgery studies reporting pCR rates. The “pCR” column indicates the number of pCR events within each subgroup. The “Patients” column reports the total number of patients included in each subgroup across the pooled studies. The “Proportion” column is the estimated pCR rate. The “p-value” corresponds to the statistical comparison between subgroups, calculated using a mixed-effects model and the Q-test. The “tau-square” column refers to the between-study heterogeneity variance, assessed using the maximum likelihood estimator. (Should NOT be printed in colours). *Significant p-value (<0.05). 3D: Three-dimensional radiotherapy, BED: Biologically effective dose, CTV: Clinical target volume, GTV: Gross tumour volume, IMRT: intensity-modulated radiotherapy, pCR: Pathologic complete response, PTV: Planning target volume, RT: Radiotherapy, SIB: simultaneous integrated boost, VMAT: Volumetric-modulated arc radiotherapy.

Because the primary objective was to investigate subgroup-specific associations, global pooled *meta*-analyses were not reported. The following factors were significantly associated with higher pCR rates: using IMRT/VMAT instead of 3D-RT (p = 0.007), giving a simultaneous integrated boost rather than a sequential boost (p = 0.020), delivering a boost dose > 74 Gy in BED (p = 0.035), and using a combination of induction and consolidation chemotherapy (p = 0.023). Publication year (p = 0.14), CTV (p = 0.75) and PTV (p = 0.10) definitions, concomitant chemotherapy (p = 0.10), and surgical delay (p = 0.86) were not associated with pCR rate (Fig. 6 and Supplementary Fig. 5 for detailed results).

Meta-regression confirmed that the boost BED dose was a significant continuous predictor of pCR (p < 0.001, Appendix C). Between-study heterogeneity was substantial, with I2 values ranging from 55 % to 81 % across subgroups (Supplementary Table 8). Significant publication bias was detected (Peters’ test p-value = 0.013, Supplementary Fig. 6).

Results regarding cCR in W&W and LRR in planned surgery and W&W studies are described in Appendix B.

## Discussion

This systematic review highlights a substantial heterogeneity in how external beam radiotherapy (EBRT) rectal boosts are implemented across institutions worldwide. This variability is exemplified by the wide range of CTV definitions (Fig. 3C), the 52 different dose prescriptions (Fig. 4A), and the 13 different concomitant chemotherapy regimens (Fig. 5B). Such diversity underscores the need for international guidelines to standardise RT rectal boost protocols in LARC. Our systematic review suggests that, in current practice, most centres use modern RT techniques (IMRT/VMAT) to deliver either a sequential or simultaneous boost, typically in supine position. Bladder filling protocols are frequently applied, whereas rectal preparation protocols remain uncommon. Boost CTV delineation is highly inconsistent, varying from GTV alone to the entire mesorectum. The median BED of the boost delivered to the primary tumour (70.5 Gy) remains modest, corresponding to approximatively 50–54 Gy in 25 fractions, a standard prescription. Concomitant radiochemotherapy is common, most often based on fluoropyrimidines. However, the use of induction and/or consolidation chemotherapy remains limited to more recent studies. Meta-analysis results demonstrate that the use of modern RT techniques (IMRT/VMAT) compared to 3D, a simultaneous integrated boost compared to a sequential boost, and higher BED doses (>74  Gy) are all significantly associated with improved pCR rates. Due to the limited number of studies reporting outcomes in the W&W setting, no conclusion can be drawn regarding cCR. In the following sections, we summarise the main findings in light of the current literature and provide technical insights to support the implementation of rectal boost EBRT in LARC patients.

In rectum cancer, only a limited number of studies have compared 3D with IMRT/VMAT. Dosimetric studies have shown that IMRT provides superior target coverage conformity and improved sparing of organs at risk (OARs) compared to 3D [103,110]. However, retrospective comparative studies, typically involving small cohorts, failed to demonstrated any difference in response and survival outcomes, although they suggest a reduction in radiation-induced toxicities with IMRT/VMAT [84,111]. Our descriptive analysis confirms that these conformal techniques are now widely established in clinical practice. Moreover, the *meta*-analysis supports the superiority of IMRT/VMAT over 3D when delivering an EBRT boost to the primary tumour. This analysis does not aim to provide conclusions regarding alternative rectal boost modalities such as contact x-ray brachytherapy. Prospective trials are required to validate whether the observed improvements in pCR rates can translate into higher cCR and organ preservation rates, as well as improved OAR sparing—outcomes that were beyond the scope of the present work.

By shortening the overall treatment time and increasing the dose per fraction, a simultaneous integrated boost may theoretically limit tumour cell repopulation and increase tumour control, which has never been confirmed in other cancer type trials or in rectal cancer retrospective publications [37,69,84,[112], [113], [114], [115], [116], [117]]. However, dosimetric and toxicity benefits have been highlighted for simultaneous boost for rectal cancers as well as in other cancers [37,84,[112], [113], [114]]. Additionally, simultaneous integrated boost also offers practical benefits, including reduced number of treatment sessions, with potential economic and patient comfort advantages [118]. While our descriptive analysis indicates that sequential and simultaneous boosts are used with similar frequency, our *meta*-analysis shows that delivering the boost simultaneously with pelvic irradiation is significantly associated with higher pCR rates compared to sequential approaches. These findings support the inclusion of simultaneous integrated boost in future recommendations to encourage its broader adoption in clinical practice.

Rectum and bladder preparations are important for the successful completion of RT in rectal cancer, serving 2 main objectives: (1) limiting toxicity by displacing the small bowel and other pelvic OARs out of the high-dose region, and (2) ensuring interfractional anatomical reproducibility, thereby avoiding dosimetric deviation from the scheduled RT plan [[119], [120], [121]]. In practice, although strict rectal preparation protocols are feasible, they are subject to random daily variations and patient compliance issues. These uncertainties must be accounted for in PTV margins [121,122]. Technologies such as CBCT and MRI-guided online-adaptive RT are emerging as effective solutions to manage daily anatomical variability and are particularly relevant in pelvic RT, where interfraction organ motion is considerable [123,124]. Several teams have already implemented online-adaptive RT for rectal cancers, showing promising findings regarding PTV reduction and dosimetric results [[125], [126], [127]]. In our systematic review, most studies reported using a full bladder protocol, while rectal preparation was less consistently addressed. Regarding treatment position, both prone and supine offer advantages: prone position can reduce the small bowel dose, while supine position improves reproducibility and patient comfort. With advances in RT delivery and image-guidance, the differences between these positions are becoming less relevant [[128], [129], [130]]. Our analysis revealed that both were commonly used, with a trend toward increased use of the supine position in recent years.

The greatest interstudy variability was observed in the boost volume definitions (GTV, CTV, PTV). Furthermore, several studies did not use the standardised denomination recommended by the International Commission on Radiation Units and Measurements (ICRU) report No. 83 [131], leaving therefore a risk of inaccurate results. Current guidelines recommend the integration of DRE, endoscopy and MRI to guide the delineation in rectal cancer. Particularly, high-resolution T2-weighted MRI allow direct visualisation and accurate delineation of the tumour and its relationship to the mesorectal fascia [132]. While the use of PET-CT for GTV delineation is not recommended, preliminary studies suggest it may reduce interobserver delineation variability and GTV size [97,[133], [134], [135]]. Diffusion-weighted MRI also appears promising, with data suggesting improved interoperator reproducibility and further GTV reduction when compared to PET-CT [136]. In our descriptive analysis, MRI was the most frequently reported imaging modality for GTV definition and should be recommended for this purpose. The lack of consistent reporting regarding volume definition and imaging modalities underscores once again the need for clear guidelines on rectal boost delineation, although such recommendations already exist for the elective CTV (pelvic lymph nodes) definition [12]. This gap was reflected in our findings: some teams applied numerical margins around the GTV, others used anatomical landmarks, and some combined both. The resulting CTV volumes ranged widely, from the primary tumour alone to the entire mesorectum or even presacral space. Importantly, the *meta*-analysis results showed that the CTV definition did not significantly impact CR rate or LRR. These results, aligned with the objective of non-operative management and limitation of total irradiated volume, support the consideration of no additional GTV to CTV margin, especially given that the whole rectum and mesorectum are already irradiated as part of the prophylactic pelvic RT [12]. Future trials should investigate whether limiting the boost CTV could safely validate those principles. PTV margins were most often defined empirically and isotropically, though not always supported by experimental and clinical evidence. Anisotropic PTV expansions have also been used to account for the variability of rectal motion in the 3 dimensions, which is greater in the antero-posterior and supero-inferior axes than in the left–right axis [[137], [138], [139]]. Other groups have suggested more individualised PTV definitions incorporating setup errors, delineation errors, rectal motion, gender, and tumour location [86,127,128,140]. The *meta*-analysis found no impact of the PTV boost definition on RT outcomes, suggesting that margin definition should be adapted to each institution's protocols (image-guided RT, adaptive RT, RT preparation, etc.).

Two neo-adjuvant RT regimens are currently recommended for LARC: (1) short-course RT (25 Gy in 5 fractions), and (2) long-course RT (45–50 Gy in 25 fractions with a boost up to 54–56 Gy) [2]. A *meta*-analysis by Burbach et al. (2014) showed that escalating the dose above 60 Gy (EQD2) was associated with high pCR-rate, good resectability and acceptable toxicity [10]. Our systematic review revealed that the clinical practice often diverges from standard RT fractionations, with more exotic schemes. We converted all boost doses in BED and categorised them into 3 categories: (1) BED < 68 Gy, mainly short-course regimens; (2) BED 68 – 74 Gy, standard long-course regimens; and (3) BED > 74 Gy, dose-escalation regimens. Both subgroup *meta*-analysis and *meta*-regression demonstrated a positive correlation between dose escalation and pCR, although the absolute level of escalation remains modest (e.g. 74 Gy BED ≈ 25 × 2.09 Gy). In contrast, no association was found in W&W studies or for LRR, likely due to smaller cohorts in the W&W analysis and heterogenous follow-up for LRR. Nevertheless, these findings support further exploration of rectal boost dose escalation, provided toxicity remains acceptable, which was not assessed here.

Single-agent fluoropyrimidine is the standard during long-course RT. Doublet regimens demonstrated a higher toxicity without consistent oncological benefit [[141], [142], [143], [144], [145]]. In our analysis, over 70 % of cohorts used 5-FU or capecitabine alone, and doublet regimens did not improve outcomes. TNT, validated in recent phase III trials (PRODIGE-23 and RAPIDO), remains a new paradigm, with both studies published in 2021 [3,4]. Reflecting this, only 14 of the 83 publications included in our descriptive analysis reported the use of induction and/or consolidation chemotherapy. In the *meta*-analysis, only the combination of induction and consolidation chemotherapy showed a significant pCR benefit. However, this was based on one retrospective and one small phase I study, deserving confirmation in prospective studies [74,95]. Unexpectedly, an increased LRR was observed in W&W cohorts using induction or consolidation chemotherapy, coming exclusively from the “OPRA” study [5]. The proportion of patients managed with a non-operative approach in the “OPRA” study was higher than other W&W studies—except the study of Boeke et al., 2022, which included only five patients with limited follow-up [5,6,8,42,76]. This study likely applied less stringent criteria for non-operative management, therefore leading to the inclusion of patients with residual disease despite initial cCR after TNT and a higher risk of local recurrence. Also, these results could be due to the interstudy difference in follow-up. These results should be taken cautiously, and no conclusions should be drawn over those of randomised phase III trials, without future robust prospective evaluations. Finally, most studies evaluating induction and/or consolidation chemotherapy focused on survival endpoints, not local control, limiting their integration in this *meta*-analysis.

Planned surgery remains the standard approach, with a 6–8-week delay between neo-adjuvant treatment and surgery being associated with higher pCR rates [2,146]. However, the W&W strategy is gaining interest, aiming to preserve organ and function and the avoidance of a surgery in patients achieving cCR, thereby improving quality of life [7]. With TNT, W&W patients can reach over 50 % of three-year total mesorectal excision-free rates [5]. The recommended clinical evaluation after neo-adjuvant therapy includes DRE, rectoscopy, and MRI, the modalities that are most frequently reported in our descriptive analysis. In planned surgery cohorts, no significant pCR or LRR difference was observed between surgical delays <9 weeks and ≥9 weeks. However, these results are limited by the small number of studies in the second category.

Regarding limitations of this study, to provide the most up-to-date information in the descriptive analysis, we included only the most recent publication from each team, which may not always reflect the most detailed descriptions of RT boost techniques. Also, although our research algorithm was chosen to be as wide-ranging as possible, some relevant publications may have been overlooked due to the extensive literature on rectal radiotherapy. A considerable proportion of studies were excluded based on the quality evaluation: 27.8 % (32/115) in the descriptive analysis and 44.7 % (63/141) in the *meta*-analysis, due to stringent criteria. This ensure high-quality data but may have led to the exclusion of studies that could have significantly influenced the *meta*-analysis results (e.g., studies with large cohorts) or that had strong methodological designs (e.g., prospective studies). The *meta*-analysis aimed to identify RT parameters associated with increased complete response rates, potentially guiding strategies to expand eligibility for W&W. However, the W&W literature remains limited, with short follow-up and small cohorts, contributing to inconclusive findings, particularly regarding induction and/or consolidation chemotherapy. To limit this bias, only studies with at least 12 months of follow-up were included. In contrast, planned surgery publications were more numerous, providing valuable findings on RT practices to optimise pCR. Still, although pCR is associated with better long-term oncological outcomes, its translation into cCR for a W&W strategy remains uncertain [16,147,148]. Each factor in the *meta*-analysis was analysed independently, which may be misleading. For instance, IMRT/VMAT use increased significantly over time (p < 0.001), as did the use of simultaneous boosts (p = 0.09) and the use of induction and/or consolidation chemotherapy. These co-evolutions undermine the assumption of variable independence. Since all the evaluated factors were inconstantly reported in a high proportion of the studies, adjusting the results for dependency would require extensive imputation of missing values, which could distort representativeness. Additionally, we focused exclusively on RT parameters, whereas tumour, biological, and socio-economic factors also influence p/cCR [146,[149], [150], [151], [152], [153]]. Important tumour-related predictors include the tumour size [146,149,151,153], the T stage [146,149,151], the N-stage [146], the differentiation grade [146,151], the circumferential invasion [151], the macroscopic ulceration [151]. In some studies, RT-related parameters (RT technique, RT dose, surgical delay) were not associated with pCR [151,153]. Biological parameters such as low carcinoembryonic antigen, neutrophil-to-lymphocyte ratio, and albumin levels, or high haemoglobin and lymphocyte counts, have also been associated with higher pCR probability, sometimes more than demographic features including age, gender, and body mass index [149,151]. In addition, advanced methods like machine learning and genomic profiling are being explored to predict pCR in LARC, further underlining the relevance of this outcome in LARC patients. Therefore, RT parameters should not be viewed as the sole determinants of treatment response, and potential confounding factors must be acknowledged. Regarding LRR, given the variable follow-up durations of the included publications, results should be interpreted cautiously (Appendix B). Finally, interstudy heterogeneity was high, particularly for pCR and cCR results, calling for cautious interpretation. Significant publication bias was also detected in planned surgery studies but not in W&W studies. However, Peter’s test for publication bias analysis lacks power when the cohort number is lower than 10, as was the case for W&W publications [154].

## Conclusion

This systematic review highlights the important variability in how EBRT boosts to the primary rectal tumour are delivered in patients with LARC worldwide. The *meta*-analysis provides insights into RT technique, the RT preparation, the RT boost delineation, the RT dose, the chemotherapy, and the treatment strategy. It demonstrates that higher pCR rates are significantly associated with the use of IMRT/VMAT over 3D, simultaneous rather than sequential boost delivery, RT dose escalation, and the combination of induction and consolidation chemotherapy. However, these results should be interpreted cautiously, as the analysis assumes independence between variables, despite known temporal and clinical interdependencies, and considers only technical treatment parameters, omitting key clinical factors correlated with pCR such as the tumour stage or size. The positive correlation between pCR and the RT dose was confirmed further by *meta*-regression, reinforcing the potential benefits of RT dose escalation for organ preservation strategies. Nonetheless, literature on this “watch and wait” (W&W) approach remains limited. Therefore, no definitive conclusions can be drawn from this *meta*-analysis regarding RT techniques that may improve cCR in W&W settings.

In conclusion, these results underscore the urgent need for clear international guidelines on rectal boost RT. Such recommendations would help standardise clinical practice, improve comparability across future studies, and support the development of non-operative management strategies. Ultimately, this would lead to enhancement of treatment effectiveness and quality of life for patients.

## Funding statement

Julien Pierrard was funded by Fonds De La Recherche Scientifique–FNRS, grant number FC 50079 and by Varian, a Siemens Healthineers Company.

## CRediT authorship contribution statement

**Julien Pierrard:** Conceptualization, Methodology, Formal analysis, Data curation, Writing – original draft, Visualization. **Lorraine Donnay:** Writing – review & editing. **Alix Collard:** Formal analysis, Writing – review & editing. **Geneviève Van Ooteghem:** Data curation, Writing – review & editing, Supervision.

## Declaration of competing interest

The authors declare the following financial interests/personal relationships which may be considered as potential competing interests: Julien Pierrard: PhD project was partially funded by Varian, a Siemens Healthineers Company.

Lorraine Donnay: None.

Alix Collard : None.

Geneviève Van Ooteghem : None.

## Data Availability

Data will be share upon acceptable request to the corresponding author.
