# Supplementary material for: Radiotherapy boost to the primary tumour in locally advanced rectal cancer: Systematic review of practices and meta-analysis
Source: Clin Transl Radiat Oncol. 2025 Jul 13;54:101014. doi: 10.1016/j.ctro.2025.101014 (PMC12284667; doi:10.1016/j.ctro.2025.101014)
Supplement: Supplementary Data 1 [file mmc1.docx]

**Appendix A: Conversion of radiotherapy doses in biological equivalent doses.**

The conventional linear quadratic model was used to obtain the biologically effective doses (BED) for each study [1]:

$$BED=n\times d\times\left( 1+\frac{d}{\alpha/\beta} \right) Eq.A.1$$

Where *n* is the number of RT sessions, *d* is the dose per session, and *α/β* is the ratio between the linear and quadratic components of cell killing. In case of using bifractionated RT or a varying dose between different RT sessions, the modified linear quadratic model incorporating time factor published by van de Geijn et al., 1989 [2] was used:

$$BED=n\times d\times\left( 1+\frac{d}{\alpha/\beta} \right)- \frac{\ln\left( 2 \right)\times\left( t_{n}-t_{k} \right)}{\left( \alpha\times t_{p} \right)} Eq.A.2$$

Where *t_n_* is the total RT duration, *t_k_* is the time to repopulation, and *t_p_* is the doubling time for proliferation of rectal cancer. Values for rectal cancer were retrieved from the literature and set to *α/β* = 5.06 Gy [3], *α* = 0.339 Gy [3], *t_k_* = 21 days [4], and *t_p_* = 5.9 days [5].

The Appendix A Table 1 shows the BED conversion of RT doses frequently used in clinical practice

| **N fractions** | **Dose per fraction (Gy)** | **Total BED (Gy)** |
| --- | --- | --- |
| 5 | 5 | 49.7 |
| 25 | 1.8 | 61.0 |
| 25 | 2 | 69.8 |
| 25 | 2.2 | 78.9 |
| 28 | 1.8 | 68.3 |
| 30 | 1.8 | 73.2 |
| 25 | 1.96 | 68.0 |
| 25 | 2.09 | 74.0 |
| 50 (BID) | 1.2 | 69.4 |
| 25 + 5 second daily fractions the last week of treatment | 1.8 (+ second daily fraction: 1.5) | 76.2 |
| 24.4 | 2 | 68.0 |
| 26.5 | 2 | 74.0 |
| **Appendix A Table 1:** Conversion table of frequently used radiotherapy doses in BED. BED: Biologically effective dose, BID: Bis in die (Twice daily). | | |

1. Fowler, J.F., *The linear-quadratic formula and progress in fractionated radiotherapy.* Br J Radiol, 1989. **62**(740): p. 679-94.

2. van de Geijn, J., *Incorporating the time factor into the linear-quadratic model.* Br J Radiol, 1989. **62**(735): p. 296-8.

3. Suwinski, R., et al., *Moderately low alpha/beta ratio for rectal cancer may best explain the outcome of three fractionation schedules of preoperative radiotherapy.* Int J Radiat Oncol Biol Phys, 2007. **69**(3): p. 793-9.

4. Rew, D.A., et al., *Proliferation characteristics of human colorectal carcinomas measured in vivo.* Br J Surg, 1991. **78**(1): p. 60-6.

5. Fowler, J.F., *21 years of biologically effective dose.* Br J Radiol, 2010. **83**(991): p. 554-68.
