# Supplementary material for: Radiotherapy boost to the primary tumour in locally advanced rectal cancer: Systematic review of practices and meta-analysis
Source: Clin Transl Radiat Oncol. 2025 Jul 13;54:101014. doi: 10.1016/j.ctro.2025.101014 (PMC12284667; doi:10.1016/j.ctro.2025.101014)
Supplement: Supplementary Data 2 [file mmc2.docx]

Appendix B: Results of the meta-analysis for cCR in W&W studies and LRR in planned and W&W studies.

# cCR results in W&W studies

In studies evaluating W&W strategy, there was no significant impact of the RT technique (p = 0.24), the CTV definition (p = 0.69), the PTV definition (p = 0.19), the boost sequence (p = 0.07), the boost dose (p = 0.25), the concomitant chemotherapy (p = 0.36), and the induction/consolidation chemotherapy (p = 0.54) on the cCR rate. The publication year was not evaluated here since all included W&W studies were published after 2016 (Appendix B Figure 1 and 7 for detailed results).


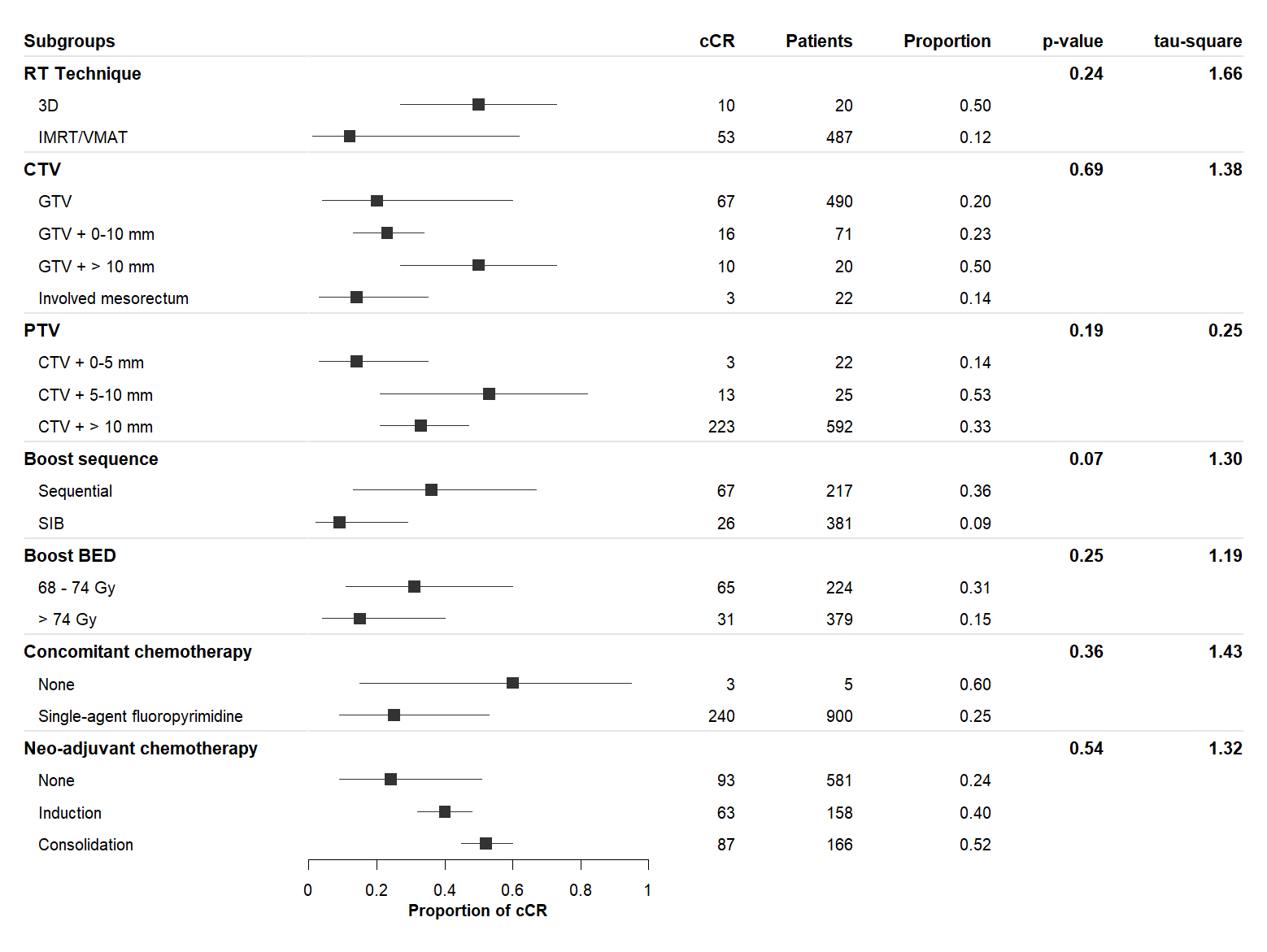


**Appendix B Figure 1:** Subgroup meta-analysis of W&W studies reporting cCR rate.

The “cCR” column is the number of cCR events within subgroup, the “Patients” column reported the total of the patients within each subgroup among the pooled publications, the “Proportion” column is the estimated cCR rate within each subgroup, the “p-value” column is the p-value for subgroup differences analysed by a mixed-effects model using the Q-test, and the “tau-square” column refers to the between-study heterogeneity variance assessed using the maximum likelihood estimator.

3D: Three-dimensional radiotherapy, BED: Biologically effective dose, CTV: Clinical target volume, GTV: Gross tumour volume, IMRT: intensity-modulated radiotherapy, pCR: Pathologic complete response, PTV: Planning target volume, RT: Radiotherapy, SIB: simultaneous integrated boost, VMAT: Volumetric-modulated arc radiotherapy, W&W: Watch and wait.

The heterogeneity among studies quantified by the I² statistics was important (range: 82 - 94%, p < 0.01) for all the parameters analysed (Appendix B Table 1).


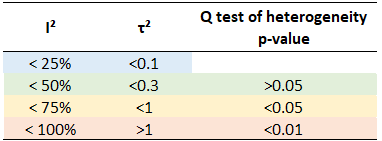


| **Meta-analysis of cCR**  **in W&W publications** | **I²** | **τ²** | **Q test of heterogeneity**  **p-value** |
| --- | --- | --- | --- |
| **RT Modulation** | 94% | 1.66 | < 0.01 |
| 3D | NA | | |
| IMRT/VMAT | 94% | 0.95 | < 0.01 |
| **CTV** | 91% | 1.38 | < 0.01 |
| GTV | 95% | 1.07 | < 0.01 |
| GTV + 0-10 mm | NA | | |
| GTV + > 10 mm | NA | | |
| Involved mesorectum | NA | | |
| **PTV** | 82% | 0.25 | < 0.01 |
| CTV + 0-5 mm | NA | | |
| CTV + 5-10 mm | 0% | 0.15 | 0.69 |
| CTV + > 10 mm | 89% | 0.15 | < 0.01 |
| **Boost sequence** | 92% | 1.30 | < 0.01 |
| Sequential | 77% | 0.58 | 0.01 |
| SIB | 93% | 0.58 | < 0.01 |
| **Boost BED** | 89% | 1.19 | < 0.01 |
| 68 - 74 Gy | 75% | 0.90 | < 0.01 |
| > 74 Gy | 92% | 0.90 | < 0.01 |
| **Concomitant chemotherapy** | 94% | 1.43 | < 0.01 |
| None | NA | | |
| Single-agent fluoropyrimidine | 95% | 1.28 | < 0.01 |
| **Neo-adjuvant chemotherapy** | 93% | 1.32 | < 0.01 |
| None | 92% | 1.06 | < 0.01 |
| Induction | NA | | |
| Consolidation | NA | | |
| **Appendix B Table 1:** Heterogeneity analysis of the cCR meta-analysis of W&W publications.  3D: Three-dimensional radiotherapy, BED: Biologically effective dose, cCR: Clinical complete response, CTV: Clinical target volume, GTV: Gross tumour volume, IMRT: intensity-modulated radiotherapy, NA: Not applicable, PTV: Planning target volume, RT: Radiotherapy, SIB: simultaneous integrated boost, VMAT: Volumetric-modulated arc radiotherapy, W&W: Watch and wait. | | | |

No significant publication bias was highlighted (Peters’ test p-value = 0.90, Appendix B Figure 2).


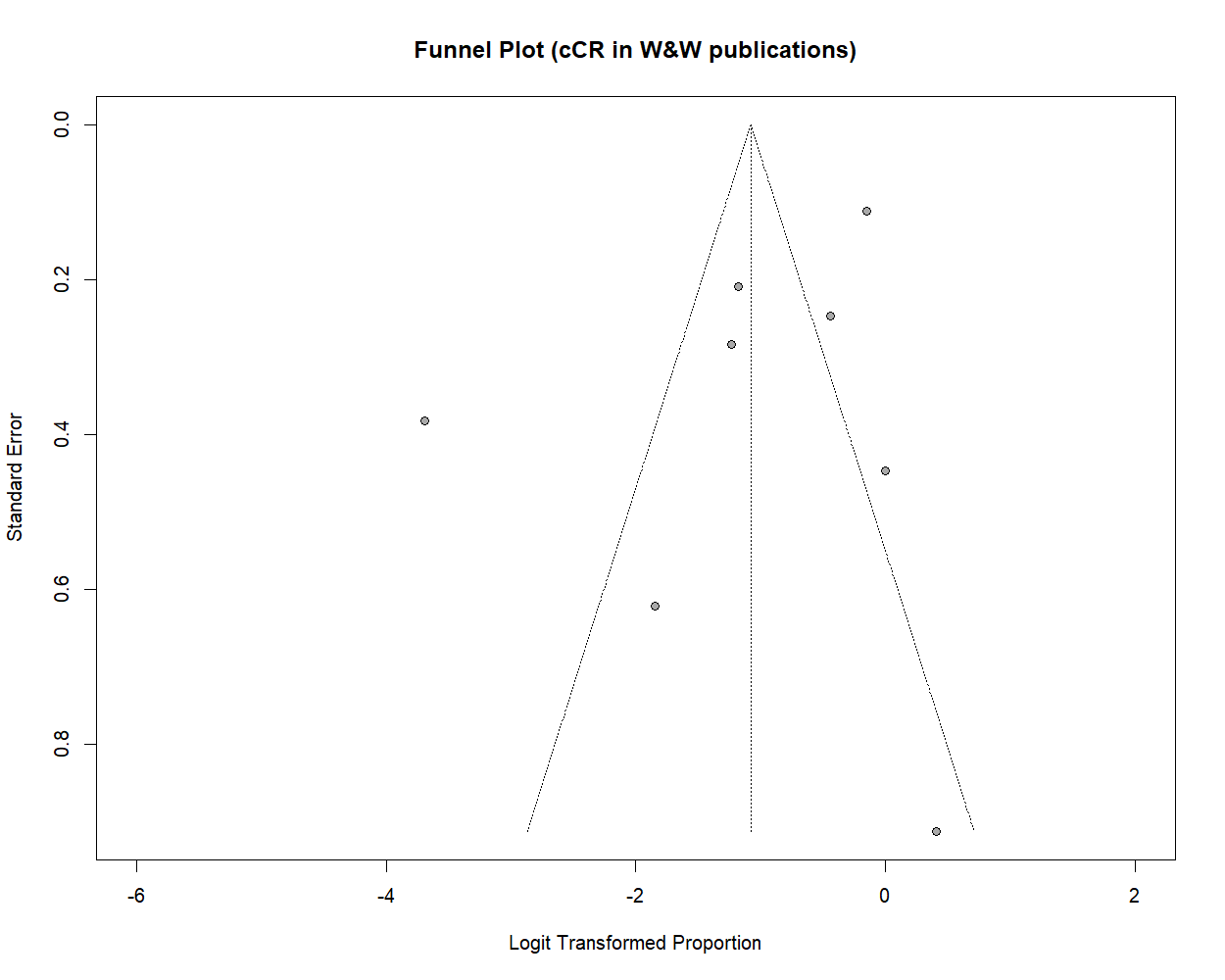


**Appendix B Figure 2:** Funnel plot of W&W publications reporting cCR.

cCR: Clinical complete response, W&W: Watch and wait.

# LRR results in planned surgery studies


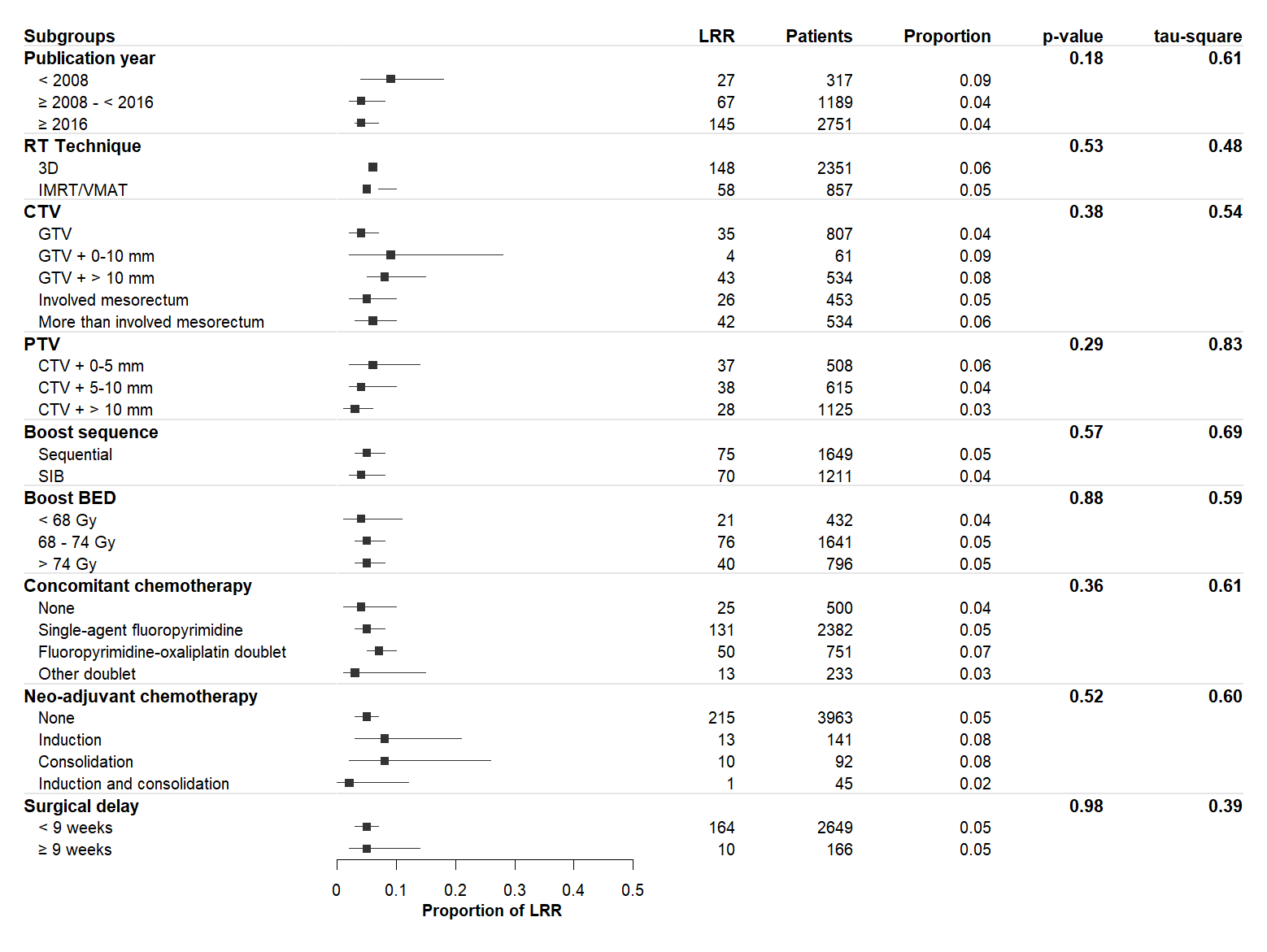
In studies with planned surgery, there was no significant impact of the publication year (p = 0.18), the RT technique (p = 0.53), the CTV definition (p = 0.38), the PTV definition (p = 0.29), the boost sequence (p = 0.57), the boost dose (p = 0.88), the concomitant chemotherapy (p = 0.36), the induction/consolidation chemotherapy (p = 0.52), and the surgical delay (p = 0.98) on the LRR rate (Appendix B Figure 3 and 8 for detailed results).

**Appendix B Figure 3:** Subgroup meta-analysis of planned surgery studies reporting LRR rate.

The “LRR” column is the number of LRR events within subgroup, the “Patients” column reported the total of the patients within each subgroup among the pooled publications, the “Proportion” column is the estimated LRR rate within each subgroup, the “p-value” column is the p-value for subgroup differences analysed by a mixed-effects model using the Q-test, and the “tau-square” column refers to the between-study heterogeneity variance assessed using the maximum likelihood estimator.

3D: Three-dimensional radiotherapy, BED: Biologically effective dose, CTV: Clinical target volume, GTV: Gross tumour volume, IMRT: intensity-modulated radiotherapy, LRR: Local relapse rate, PTV: Planning target volume, RT: Radiotherapy, SIB: simultaneous integrated boost, VMAT: Volumetric-modulated arc radiotherapy.

The heterogeneity among studies quantified by the I² statistics was moderate (range: 12 - 59%, p: < 0.01 – 0.28) for all the parameters analysed (Appendix B Table 2).

| **Meta-analysis of LRR**  **in planned surgery publications** | **I²** | **τ²** | **Q test of heterogeneity p-value** |
| --- | --- | --- | --- |
| **Publication year** | 52% | 0.61 | < 0.01 |
| < 2008 | 45% | 0.60 | 0.08 |
| ≥ 2008 - < 2016 | 49% | 0.76 | 0.02 |
| ≥ 2016 | 53% | 0.42 | < 0.01 |
| **RT Modulation** | 30% | 0.48 | 0.06 |
| 3D | 0% | 0.00 | 0.83 |
| IMRT/VMAT | 43% | 1.31 | 0.03 |
| **CTV** | 36% | 0.54 | 0.02 |
| GTV | 41% | 0.38 | 0.09 |
| GTV + 0-10 mm | 0% | 0.38 | 0.99 |
| GTV + > 10 mm | 38% | 0.38 | 0.15 |
| Involved mesorectum | 0% | 0.38 | 0.87 |
| More than involved mesorectum | 0% | 0.38 | 0.75 |
| **PTV** | 59% | 0.83 | < 0.01 |
| CTV + 0-5 mm | 35% | 0.65 | 0.15 |
| CTV + 5-10 mm | 22% | 1.40 | 0.22 |
| CTV + > 10 mm | 56% | 0.23 | 0.03 |
| **Boost sequence** | 55% | 0.69 | < 0.01 |
| Sequential | 60% | 0.44 | < 0.01 |
| SIB | 49% | 1.13 | < 0.01 |
| **Boost BED** | 48% | 0.59 | < 0.01 |
| < 68 Gy | 0% | 0.22 | 0.59 |
| 68 - 74 Gy | 61% | 0.59 | < 0.01 |
| > 74 Gy | 44% | 0.76 | 0.03 |
| **Concomitant chemotherapy** | 52% | 0.61 | < 0.01 |
| None | 55% | 0.44 | 0.05 |
| Single-agent fluoropyrimidine | 61% | 0.67 | < 0.01 |
| Fluoropyrimidine-oxaliplatin doublet | 21% | 0.00 | 0.28 |
| Other doublet | 25% | 1.29 | 0.24 |
| **Neo-adjuvant chemotherapy** | 53% | 0.60 | < 0.01 |
| None | 52% | 0.52 | < 0.01 |
| Induction | 67% | 0.52 | 0.05 |
| Consolidation | 0% | 0.52 | 0.99 |
| Induction and consolidation | NA | | |
| **Surgical delay** | 12% | 0.39 | 0.28 |
| < 9 weeks | 17% | 0.39 | 0.23 |
| ≥ 9 weeks | 0% | 0.39 | 0.95 |
| **Appendix B Table 2:** Heterogeneity analysis of the LRR meta-analysis of planned surgery publications.  3D: Three-dimensional radiotherapy, BED: Biologically effective dose, CTV: Clinical target volume, GTV: Gross tumour volume, IMRT: intensity-modulated radiotherapy, LRR: Local recurrence rate, NA: Not applicable, PTV: Planning target volume, RT: Radiotherapy, SIB: simultaneous integrated boost, VMAT: Volumetric-modulated arc radiotherapy. | | | |

Additionally, a significant publication bias was highlighted (Peters’ test p-value = 0.005, Appendix B Figure 4).


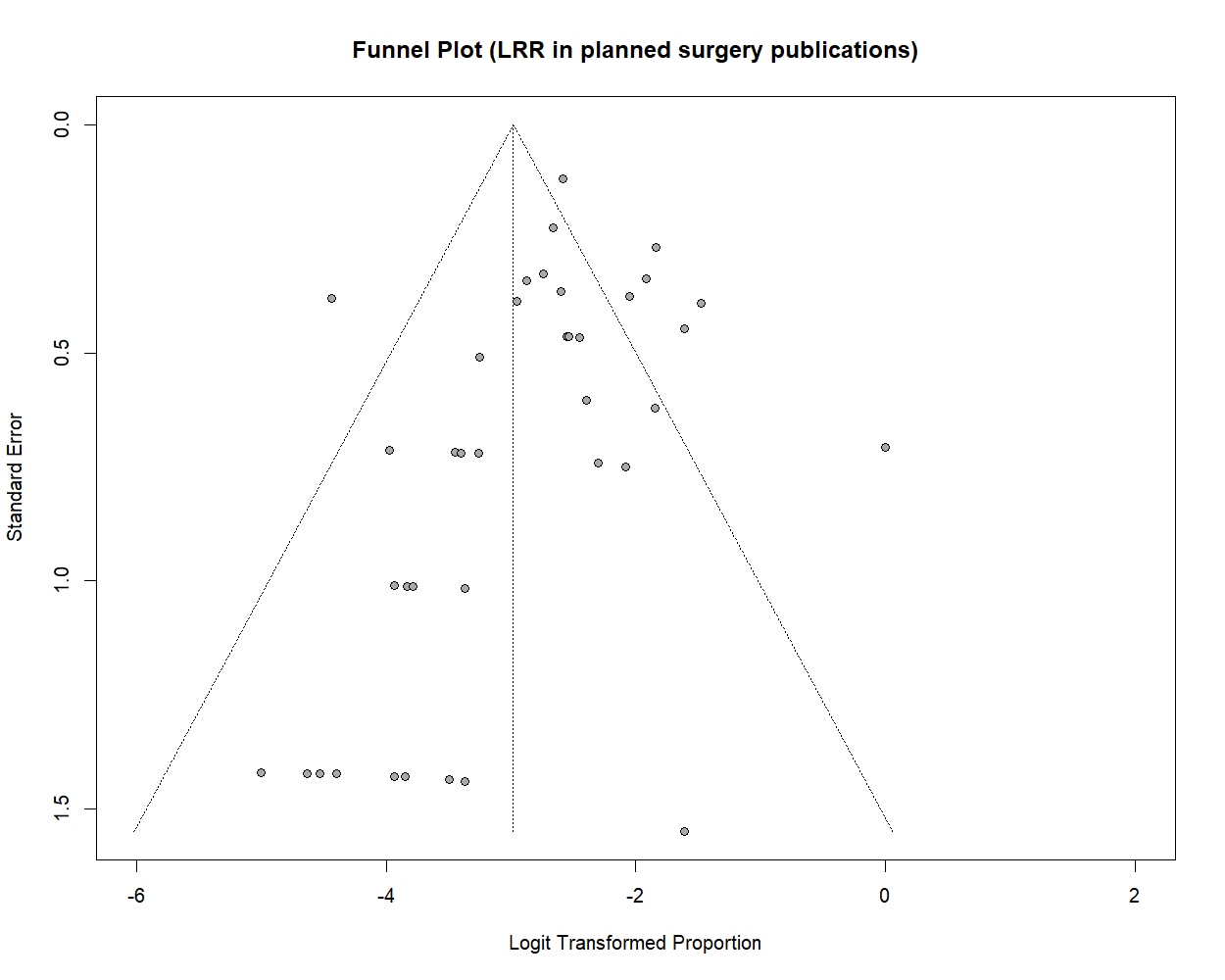


**Appendix B Figure 4:** Funnel plot of planned surgery publications reporting LRR.

LRR: Local recurrence rate.

# LRR results in planned W&W studies

In W&W studies, there was no significant impact of the RT technique (p = 0.24), the PTV definition (p = 0.97), the boost sequence (p = 0.66), the boost dose (p = 0.33), and the concomitant chemotherapy (p = 0.95). However, an exception was noted: the use of induction or consolidation chemotherapy was associated with a higher LRR (p = 0.015), which is contra-intuitive. The publication year and the CTV definition were not evaluated here since all studies are in the same category (publication year ≥ 2016, CTV defined as the GTV without margin, Appendix B Figure 5 and 9 for detailed results).


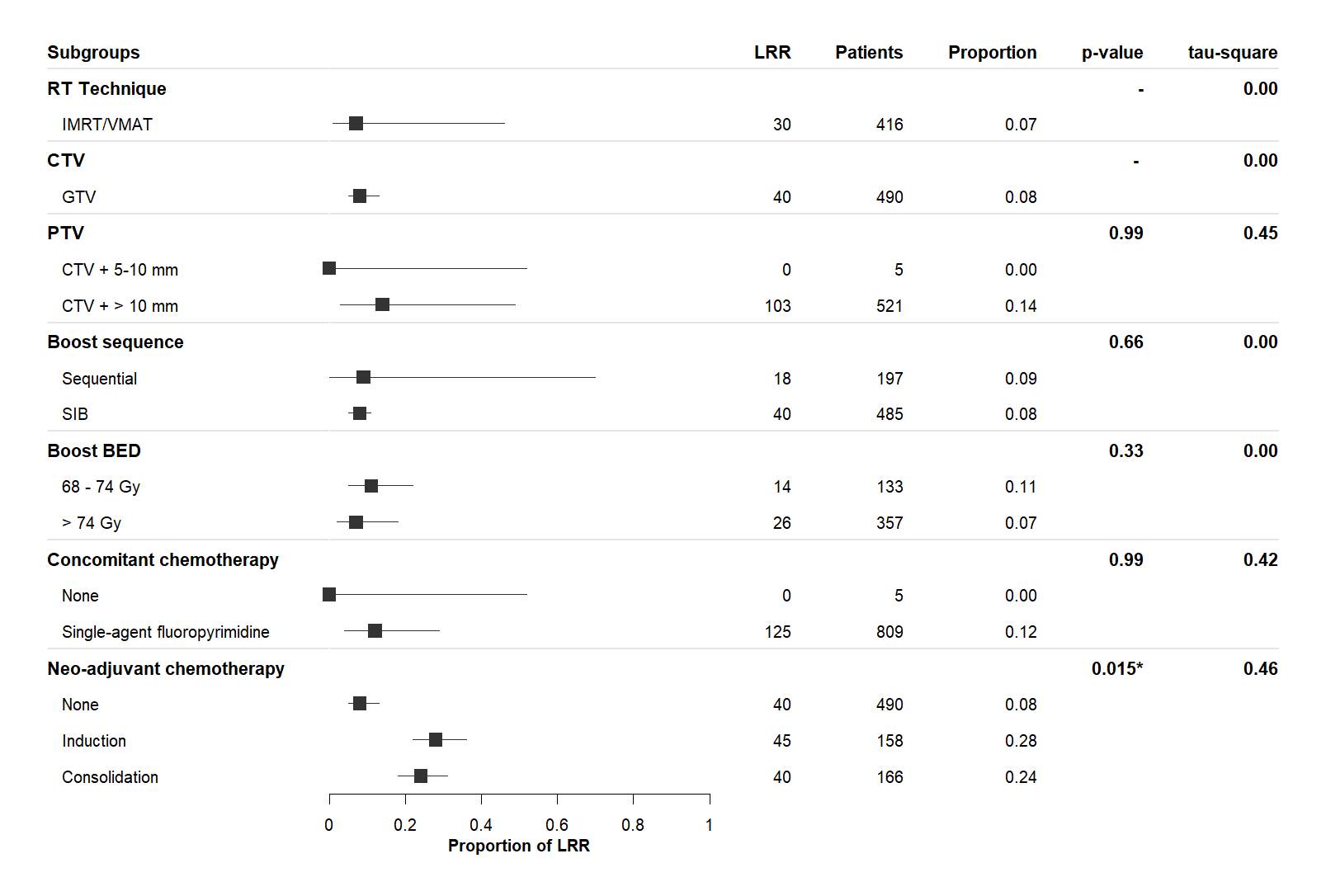


**Appendix B Figure 5:** Subgroup meta-analysis of W&W reporting LRR rate.

The “LRR” column is the number of LRR events within subgroup, the “Patients” column reported the total of the patients within each subgroup among the pooled publications, the “Proportion” column is the estimated LRR rate within each subgroup, the “p-value” column is the p-value for subgroup differences analysed by a mixed-effects model using the Q-test, and the “tau-square” column refers to the between-study heterogeneity variance assessed using the maximum likelihood estimator.

3D: Three-dimensional radiotherapy, BED: Biologically effective dose, CTV: Clinical target volume, GTV: Gross tumour volume, IMRT: intensity-modulated radiotherapy, LRR: Local relapse rate, PTV: Planning target volume, RT: Radiotherapy, SIB: simultaneous integrated boost, VMAT: Volumetric-modulated arc radiotherapy, W&W: Watch and wait.

The heterogeneity among studies quantified by the I² statistics varied widely between the parameters evaluated (range: 5 - 94%, p: < 0.01 – 0.38, Appendix B Table 3).

| **Meta-analysis of LRR**  **in W&W publications** | **I²** | **τ²** | **Q test of heterogeneity p-value** |
| --- | --- | --- | --- |
| **RT Modulation (= IMRT/VMAT)** | 0% | 0.00 | 0.61 |
| **CTV (= GTV)** | 28% | 0.00 | 0.24 |
| **PTV** | 86% | 0.45 | < 0.01 |
| CTV + 5-10 mm | NA | | |
| CTV + > 10 mm | 91% | 0.43 | < 0.01 |
| **Boost sequence** | 52% | 0.00 | 0.12 |
| Sequential | 71% | 0.00 | 0.06 |
| SIB | NA | | |
| **Boost BED** | 5% | 0.00 | 0.38 |
| 68 - 74 Gy | 56% | 0.00 | 0.13 |
| > 74 Gy | 0% | 0.00 | 0.93 |
| **Concomitant chemotherapy** | 91% | 0.42 | < 0.01 |
| None | NA | | |
| Single-agent fluoropyrimidine | 93% | 0.42 | < 0.01 |
| **Neo-adjuvant chemotherapy** | 89% | 0.46 | < 0.01 |
| None | 28% | 0.00 | 0.24 |
| Induction | NA | | |
| Consolidation | NA | | |
| **Appendix B Table 3:** Heterogeneity analysis of the LRR meta-analysis of W&W publications.  BED: Biologically effective dose, CTV: Clinical target volume, GTV: Gross tumour volume, IMRT: intensity-modulated radiotherapy, LRR: Local recurrence rate, NA: Not applicable, PTV: Planning target volume, RT: Radiotherapy, SIB: simultaneous integrated boost, VMAT: Volumetric-modulated arc radiotherapy, W&W: Watch and wait. | | | |

No significant publication bias was highlighted (Peters’ test p-value = 0.64, Appendix B Figure 6).


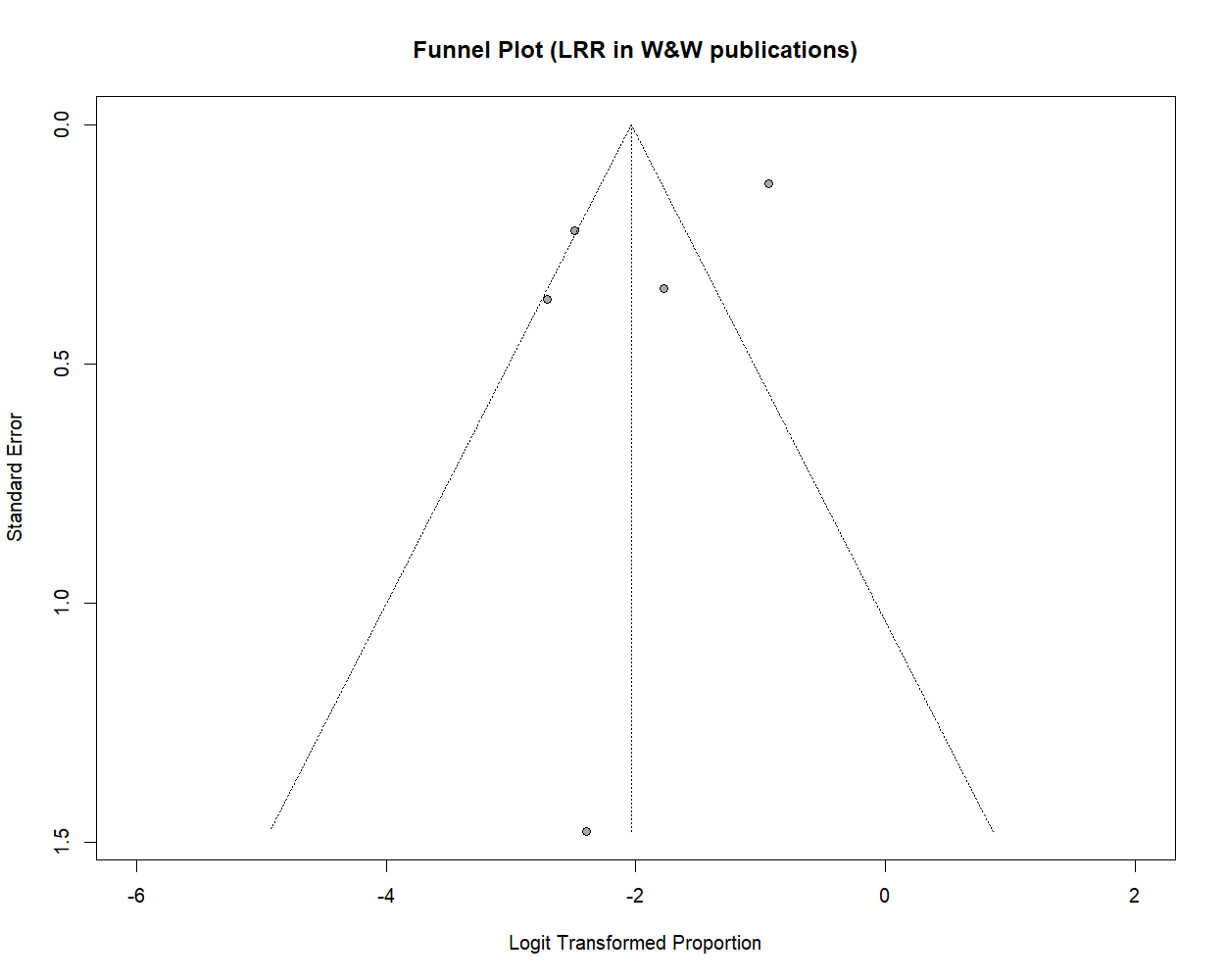


**Appendix B Figure 6:** Funnel plot of W&W publications reporting LRR.

LRR: Local recurrence rate, W&W: Watch and wait.

# Detailed meta-analysis results

4.1. cCR results in W&W studies


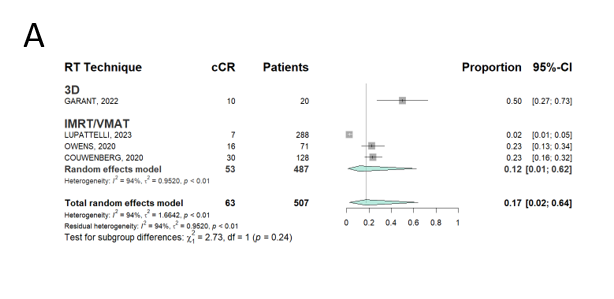


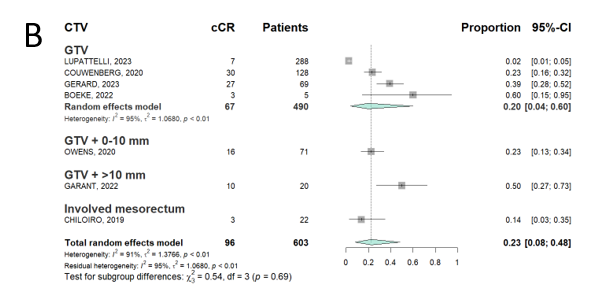


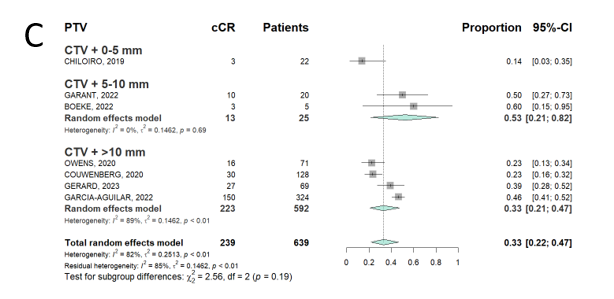


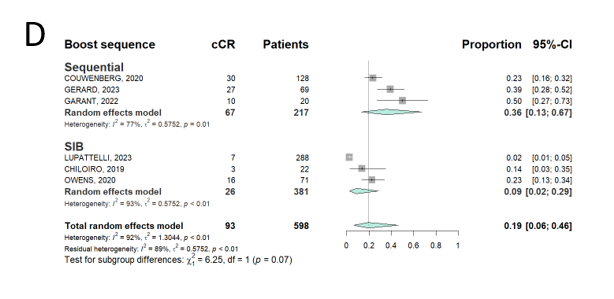


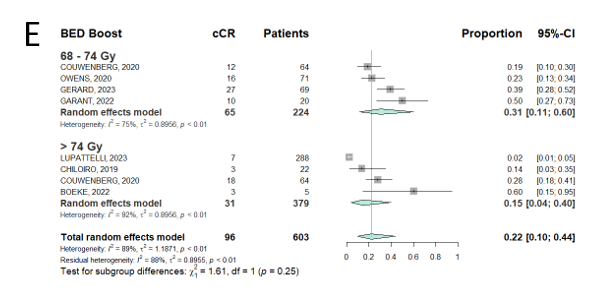


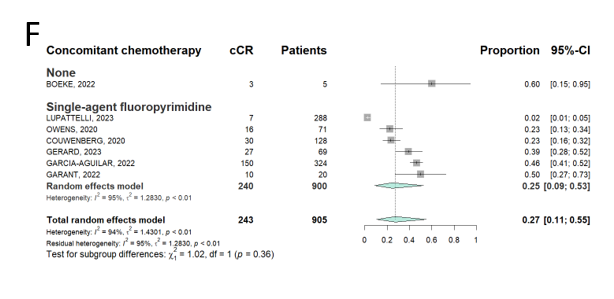


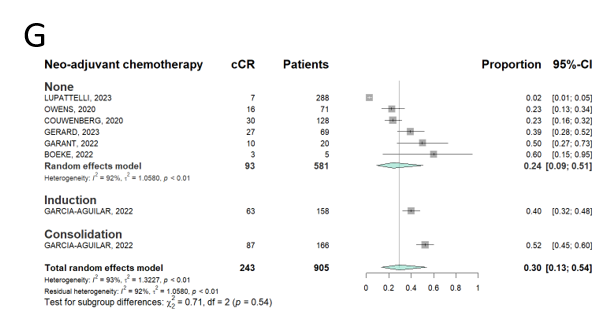


**Appendix B Figure 7:** Details of the meta-analysis of W&W studies reporting cCR rate as function of (A) RT modulation, (B) CTV definition, (C) PTV definition, (D) boost sequence, (E) boost BED, (F) concomitant chemotherapy, and (G) neo-adjuvant chemotherapy.

The “cCR” column is the number of cCR events within each study, the “Patients” column reported the total of the patients within each study among the pooled publications, the “Proportion” column is the cCR rate within each study, the “95%-CI” column is the 95% confidence interval of the LRR rate within each study.

3D: Three-dimensional radiotherapy, BED: Biologically effective dose, CTV: Clinical target volume, GTV: Gross tumour volume, IMRT: intensity-modulated radiotherapy, pCR: Pathologic complete response, PTV: Planning target volume, RT: Radiotherapy, SIB: simultaneous integrated boost, VMAT: Volumetric-modulated arc radiotherapy, W&W: Watch and wait.

4.2. LRR results in planned surgery studies


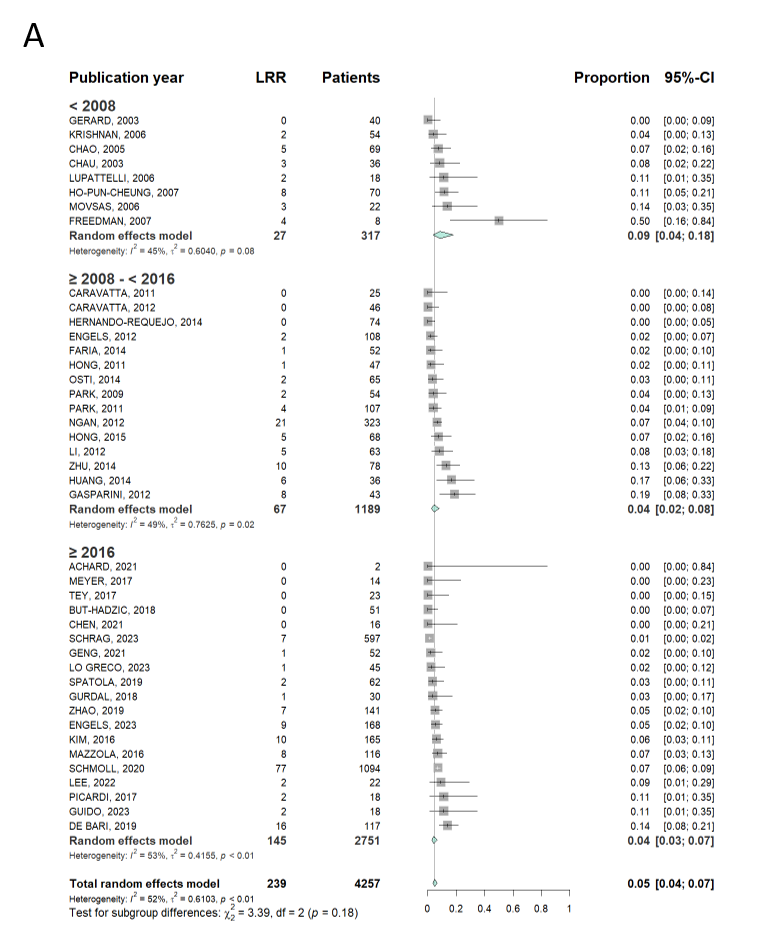


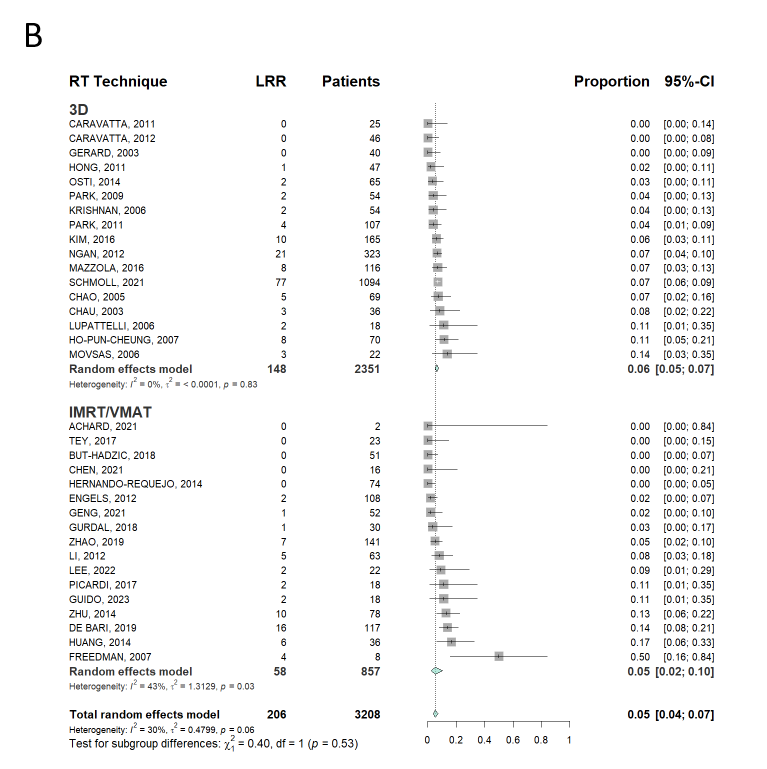


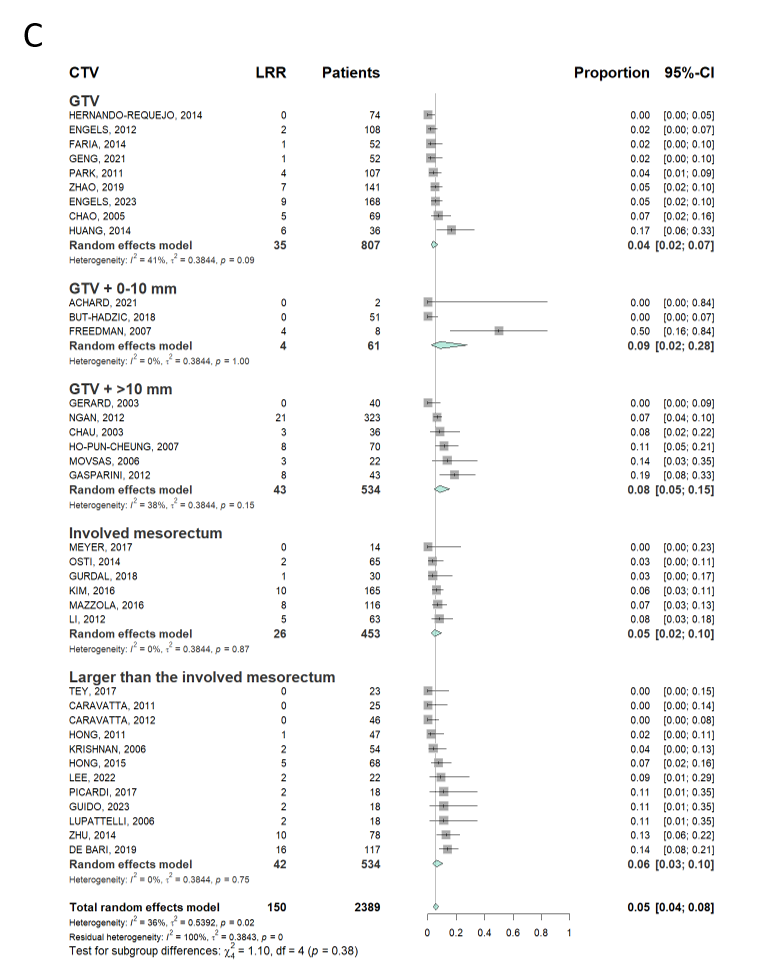


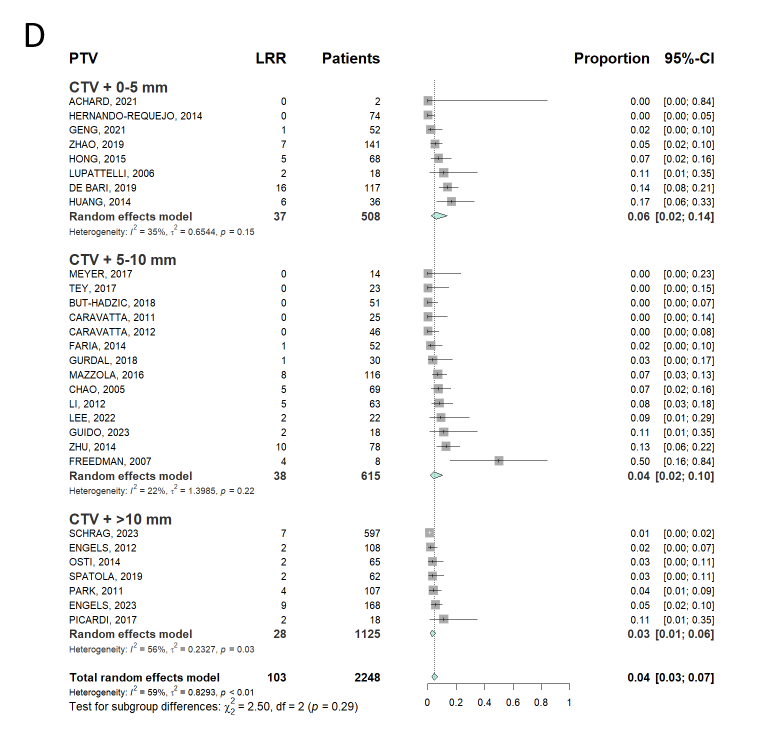


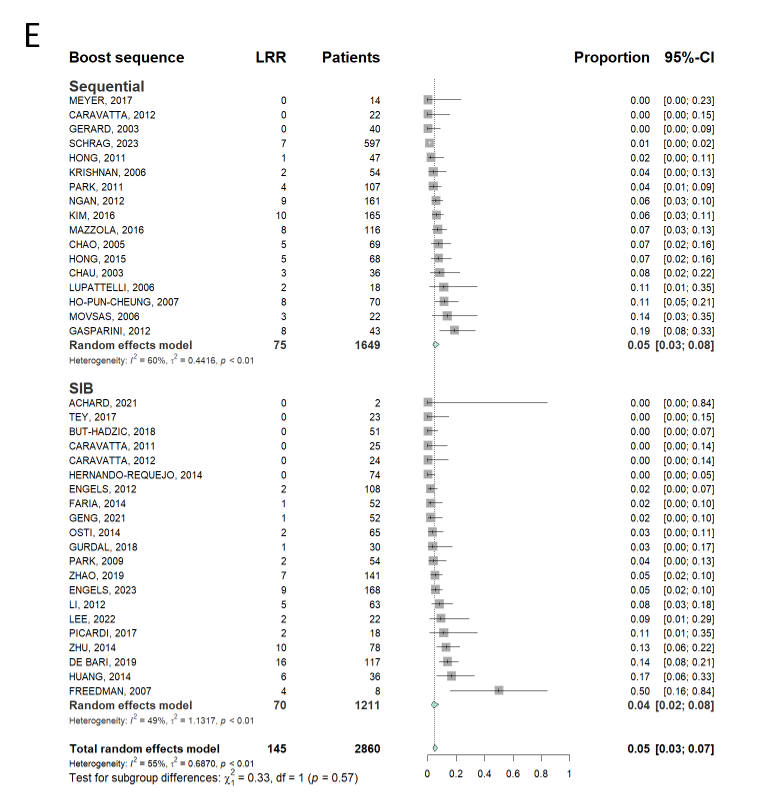


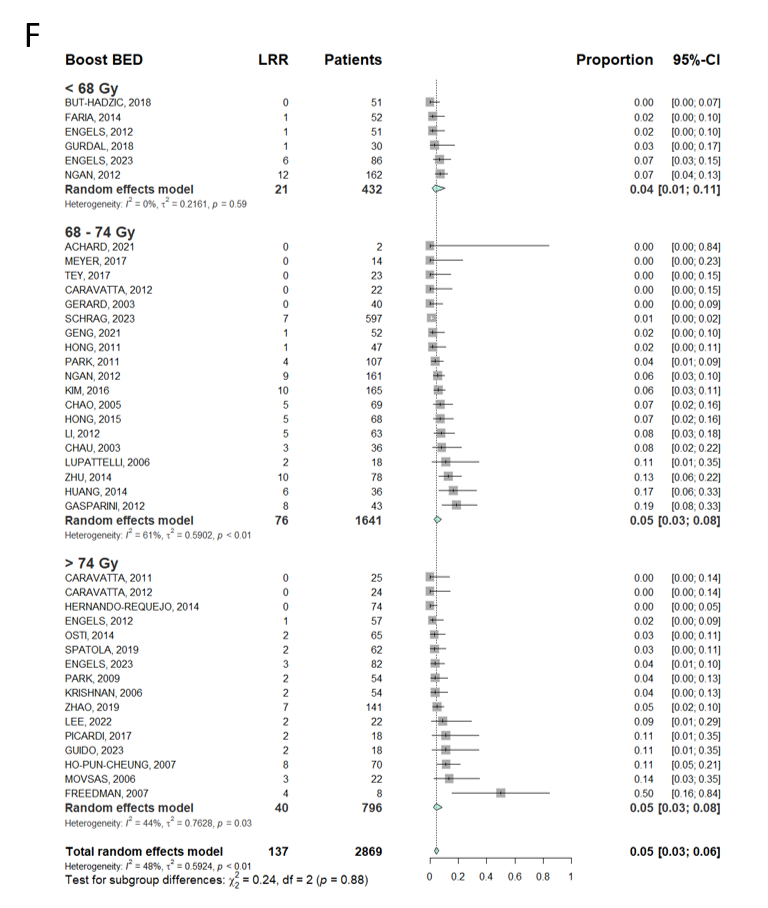


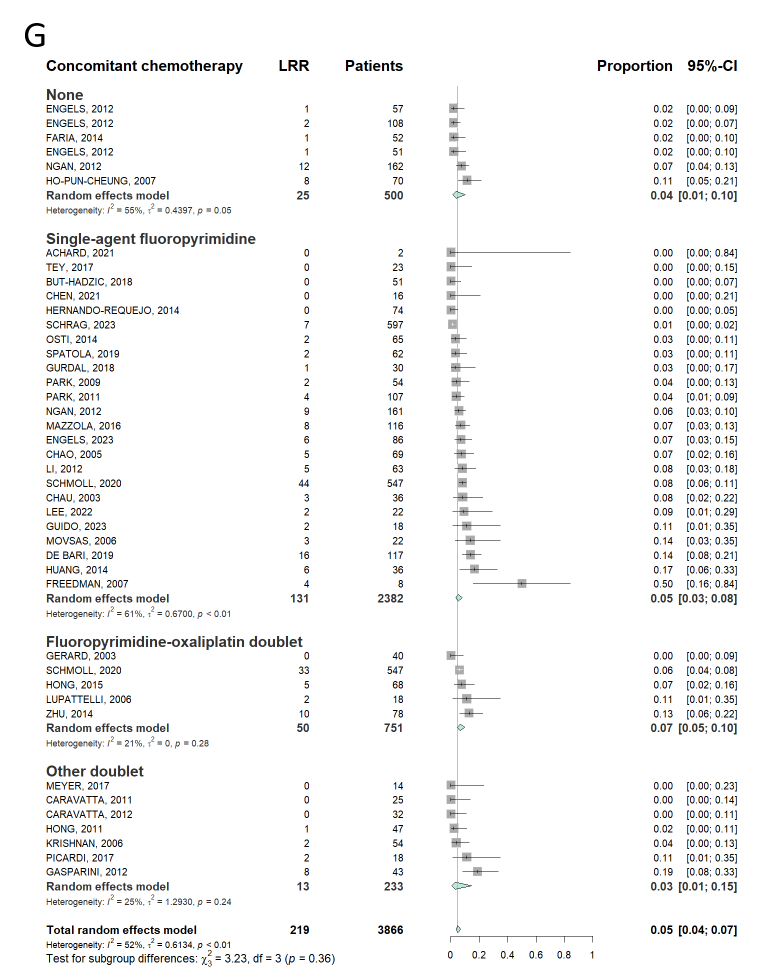


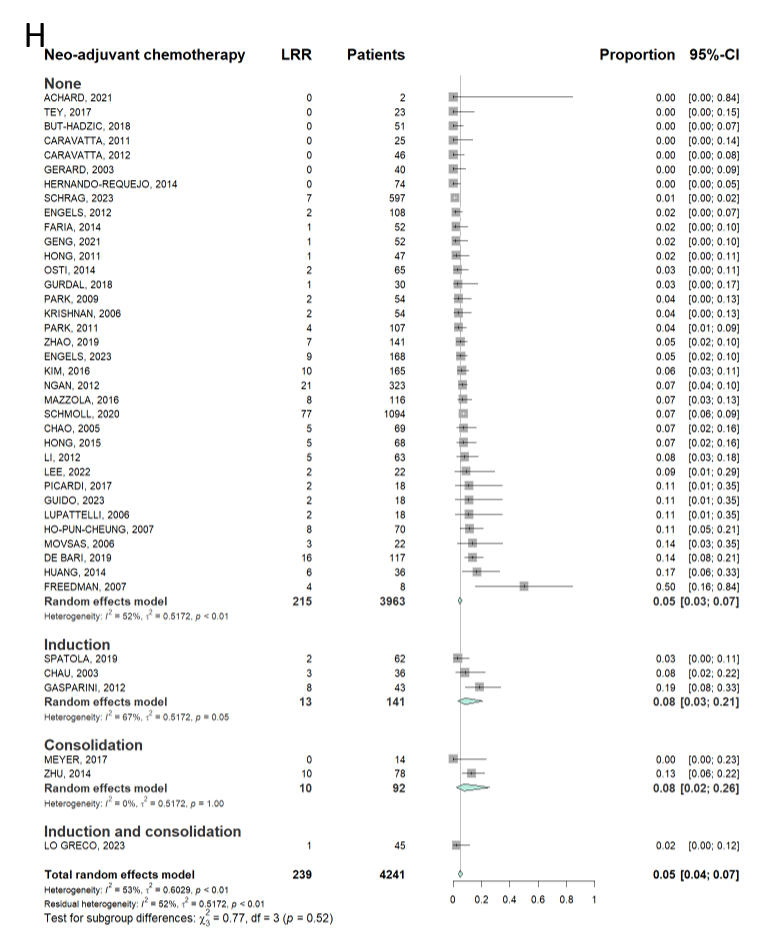


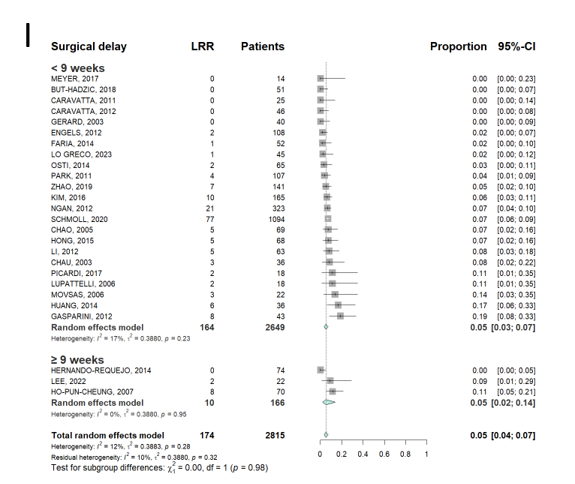


**Appendix B Figure 8:** Details of the meta-analysis of studies with planned surgery reporting LRR as function of (A) Publication year, (B) RT modulation, (C) CTV definition, (d) PTV definition, (E) boost sequence, (F) boost BED, (G) concomitant chemotherapy, (H) neo-adjuvant chemotherapy, and (I) Surgical delay.

The “LRR” column is the number of LRR events within each study, the “Patients” column reported the total of the patients within each study among the pooled publications, the “Proportion” column is the LRR rate within each study, the “95%-CI” column is the 95% confidence interval of the LRR rate within each study.

3D: Three-dimensional radiotherapy, BED: Biologically effective dose, CTV: Clinical target volume, GTV: Gross tumour volume, IMRT: intensity-modulated radiotherapy, LRR: Local recurrence rate, PTV: Planning target volume, RT: Radiotherapy, SIB: simultaneous integrated boost, VMAT: Volumetric-modulated arc radiotherapy.

4.3. LRR results in W&W studies


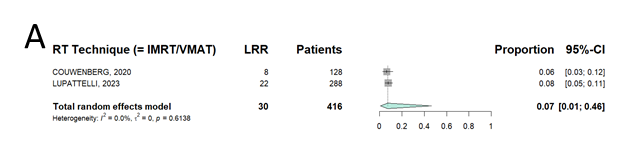


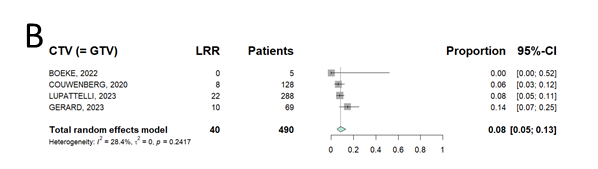


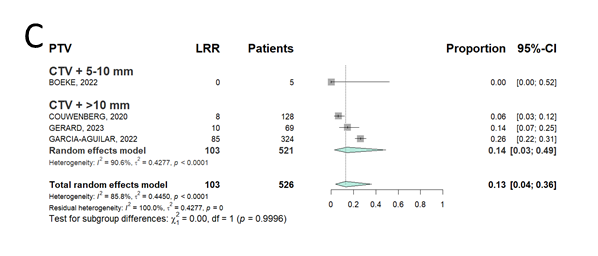


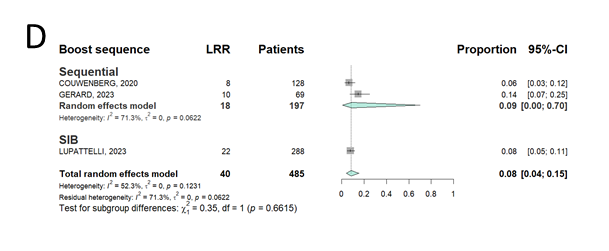


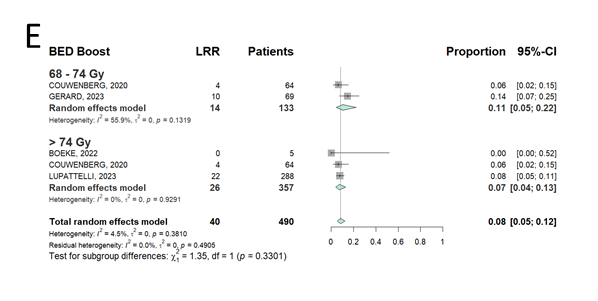

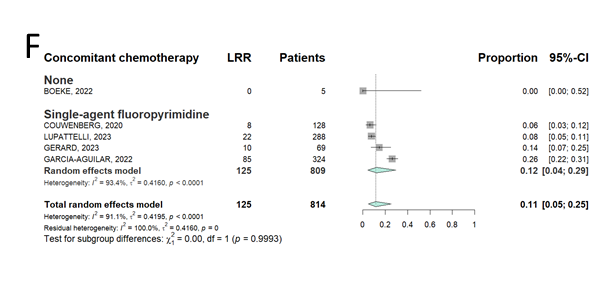


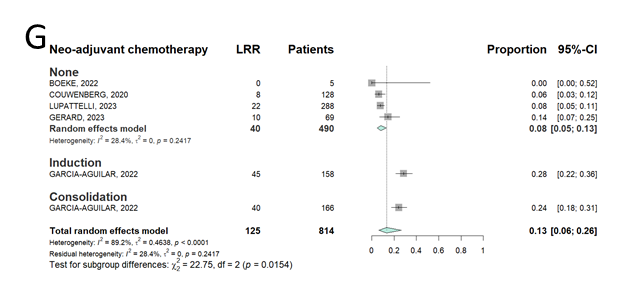


**Appendix B Figure 9:** Details of the meta-analysis of W&W studies reporting LRR as function of (A) RT modulation, (B) CTV definition, (C) PTV definition, (D) boost sequence, (E) boost BED, (F) concomitant chemotherapy, and (G) neo-adjuvant chemotherapy.

The “LRR” column is the number of LRR events within each study, the “Patients” column reported the total of the patients within each study among the pooled publications, the “Proportion” column is the LRR rate within each study, the “95%-CI” column is the 95% confidence interval of the LRR rate within each study.

BED: Biologically effective dose, CTV: Clinical target volume, GTV: Gross tumour volume, IMRT: intensity-modulated radiotherapy, LRR: Local recurrence rate, PTV: Planning target volume, RT: Radiotherapy, SIB: simultaneous integrated boost, VMAT: Volumetric-modulated arc radiotherapy, W&W: Watch and wait.
