# Supplementary material for: Radiotherapy boost to the primary tumour in locally advanced rectal cancer: Systematic review of practices and meta-analysis
Source: Clin Transl Radiat Oncol. 2025 Jul 13;54:101014. doi: 10.1016/j.ctro.2025.101014 (PMC12284667; doi:10.1016/j.ctro.2025.101014)
Supplement: Supplementary Data 3 [file mmc3.docx]

**Appendix C : Meta regression model of pathologic complete response (pCR) as a function of radiotherapy (RT) dose.**

# Meta regression methods

For cohorts reporting outcomes for a unique RT boost dose, a meta-regression model with the RT boost dose as a continuous predictor of the pCR was performed. In case of significant model, a non-linear correlation model would be used to determine pCR probability as function of the RT boost dose using a sigmoidal function.

# Meta regression results

The meta-regression model highlights that the boost biologically effective dose (BED) was significantly associated with a pCR increase. The regression coefficient was 0.043 (95% confidence interval: 0.025 – 0.062, p-value < 0.001). The boost BED explained 37.7% of the interstudy variance ($R_{*}^{2}$ coefficient). After adjusting for boost BED, moderate heterogeneity remained (I² = 65.6%). Using a non-linear correlation model, the pCR rate could be approximated using the following formula (Appendix C Figure 1):

$$pCR rate= \frac{1}{1+ e^{\frac{107.0-Boost BED}{22.8}}} Eq.C.1$$

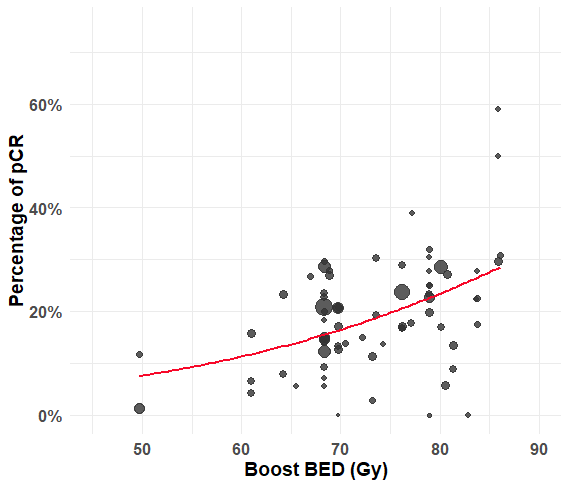


***Appendix C Figure 1:*** *Reported pCR rate as function of the boost BED delivered to the primary tumour. Each dot represents one study and its size is function of the total population of this study. The red line reveals a non-linear positive correlation between pCR and boost BED.*

*BED: Biologically effective dose, pCR: Pathologic complete response.*
