# Supplementary material for: Radiotherapy boost to the primary tumour in locally advanced rectal cancer: Systematic review of practices and meta-analysis
Source: Clin Transl Radiat Oncol. 2025 Jul 13;54:101014. doi: 10.1016/j.ctro.2025.101014 (PMC12284667; doi:10.1016/j.ctro.2025.101014)
Supplement: Supplementary Data 4 [file mmc4.docx]

**Supplementary Figures and Table**

**Pages 2 – 15: Supplementary Figures**

Page 2: Supplementary Figure 1: Flow chart for study selection at the first search.

Page 3: Supplementary Figure 2: Flow chart for study selection after the search was reloaded.

Page 4: Supplementary Figure 3: Results of the quality assessment analysis for the descriptive analysis.

Page 5: Supplementary Figure 4: Results of the quality assessment analysis for the meta-analysis.

Page 6 – 14: Supplementary Figure 5: Details of the meta-analysis on pCR rates.

Page 15: Publication bias assessment of the pCR meta-analysis

**Pages 16 – 28 : Supplementary Tables**

Page 16 – 17: Supplementary Table 1: Parameters extracted

Page 18: Supplementary Table 2: Quality assessment scale

Page 19 – 21: Supplementary Table 3: Publications included in the descriptive analysis.

Page 22: Supplementary Table 4: Publications excluded from the descriptive analysis

Page 23: Supplementary Table 5: Publications excluded from the meta-analysis

Page 24 – 26: Supplementary Table 6: Planned surgery publications included in the meta-analysis

Page 27: Supplementary Table 7: W&W publications included in the meta-analysis

Page 28: Supplementary Table 8: Heterogeneity evaluation of the pCR meta-analysis

**Pages 29 – 40 : References**

**Supplementary Figures**


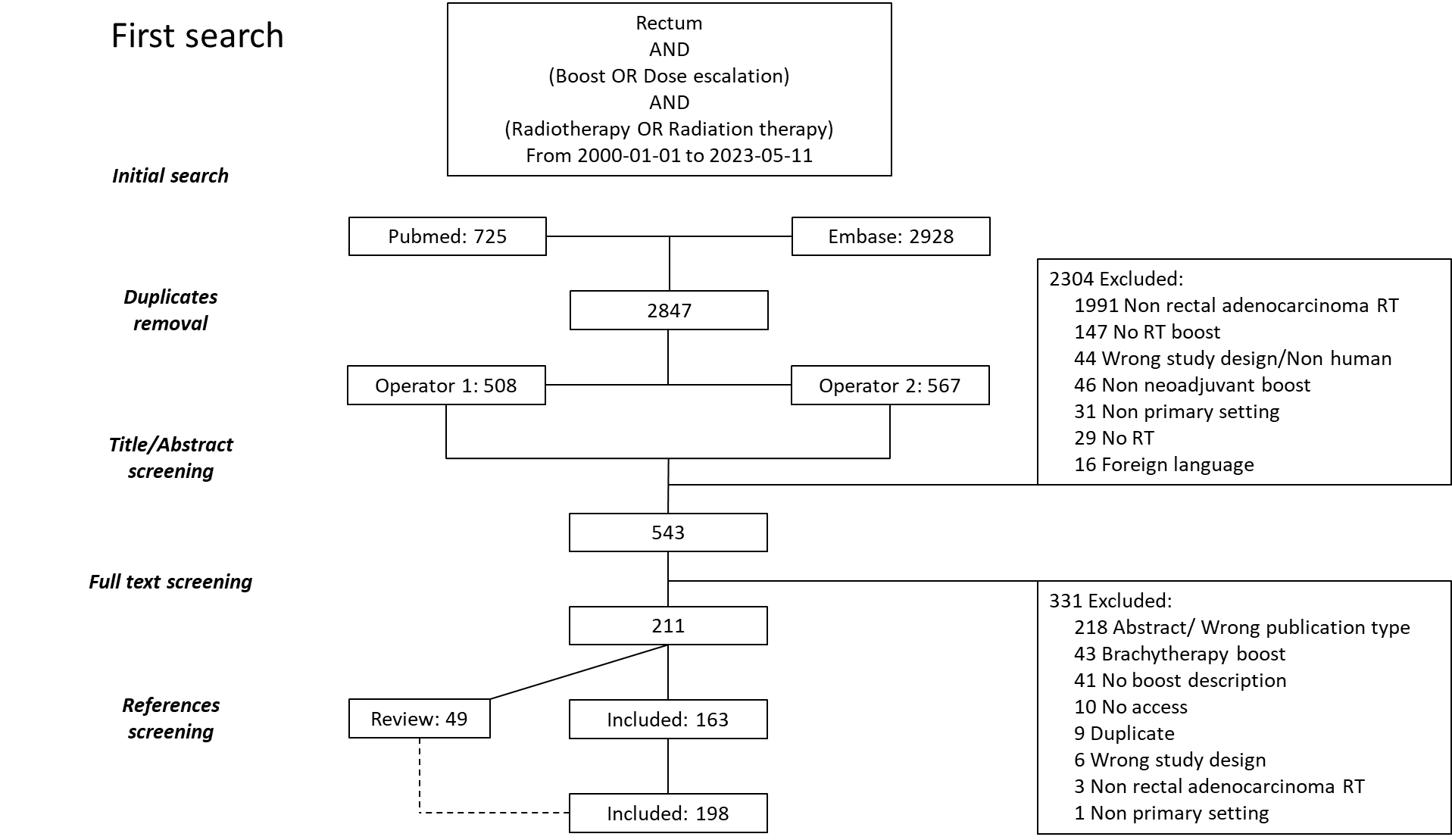


**Supplementary Figure 1:** Flow chart of study selection at the first search.

RT: Radiotherapy.


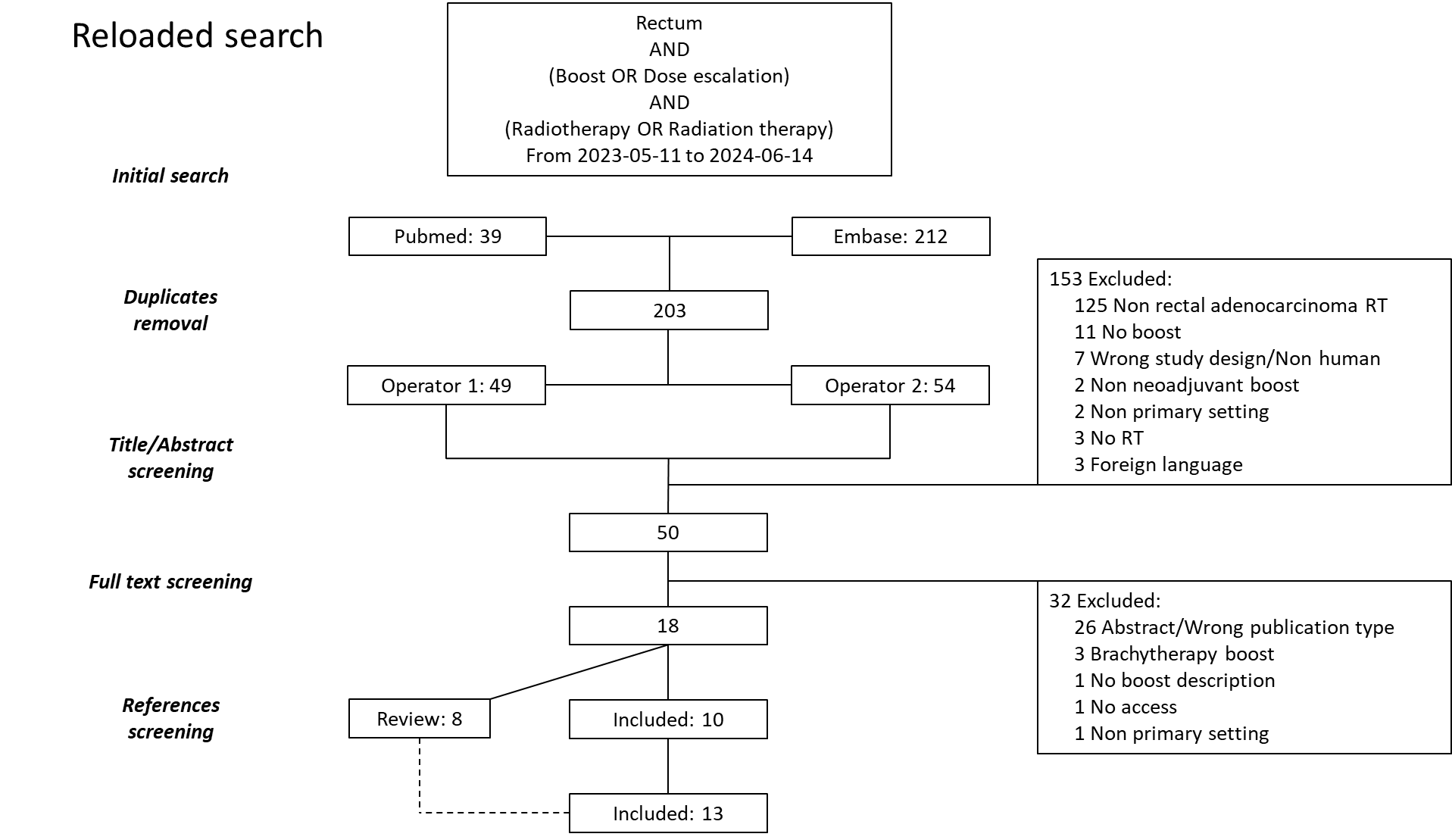


**Supplementary Figure 2:** Flow chart of study selection after the search was reloaded.

RT: Radiotherapy.


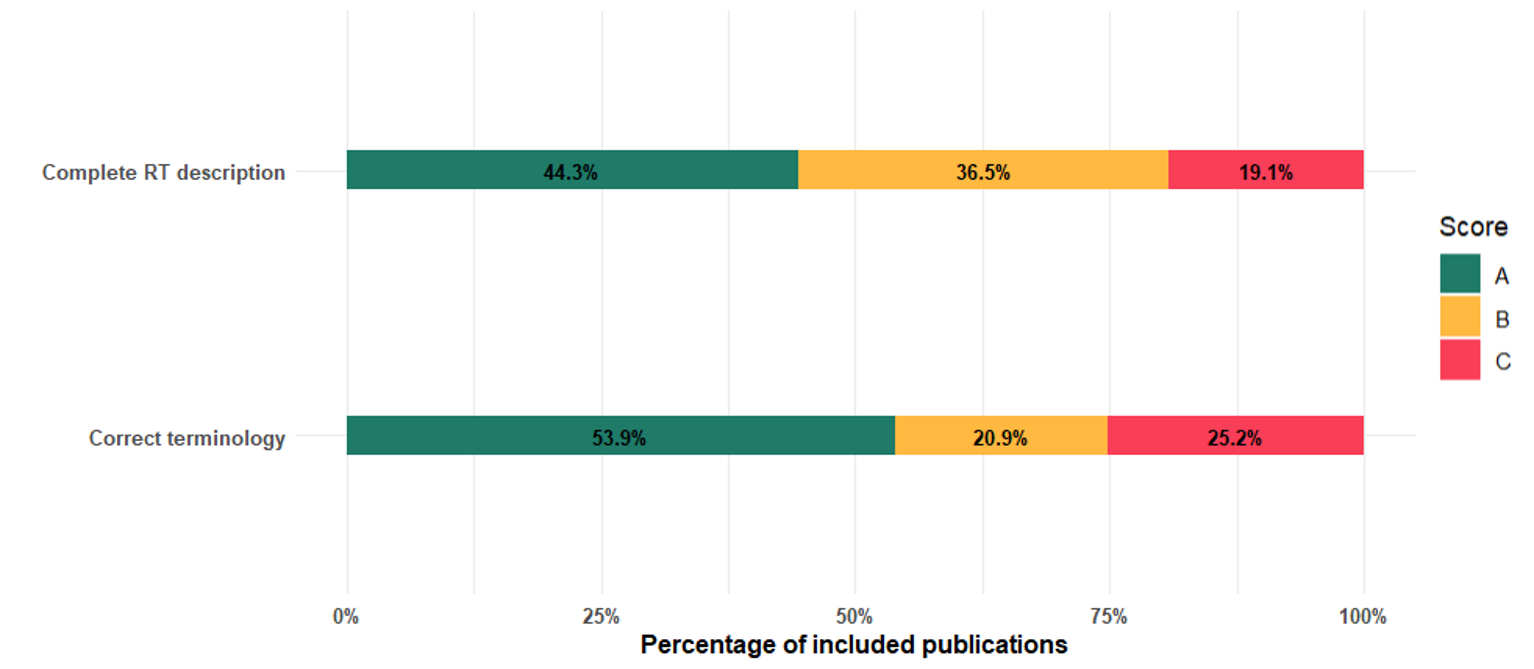


**Supplementary Figure 3:** Results of the quality assessment analysis for the 115 studies included in the descriptive analysis after institutional duplicate removal. Studies that received a score of 'C' on at least one of these items were excluded, resulting in a total of 83 studies included in the descriptive analysis.

RT: Radiotherapy.


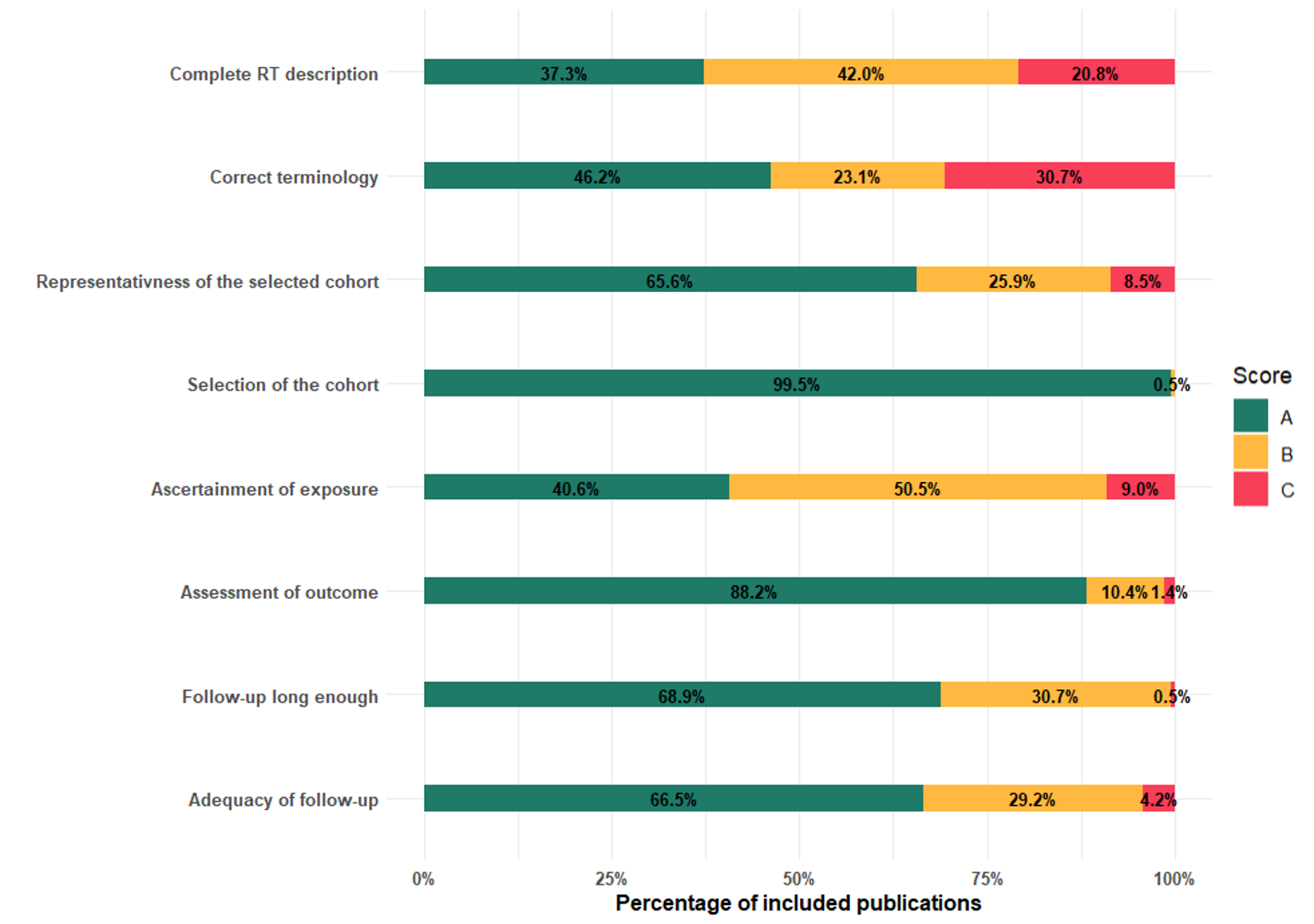


**Supplementary Figure 4:** Results of the quality assessment analysis for the 141 studies with 212 different cohorts initially included in the meta-analysis. To ensure quality, studies with a 'C' score on any of the items were excluded, leaving 78 studies with 97 different cohorts for the meta-analysis. RT: Radiotherapy.


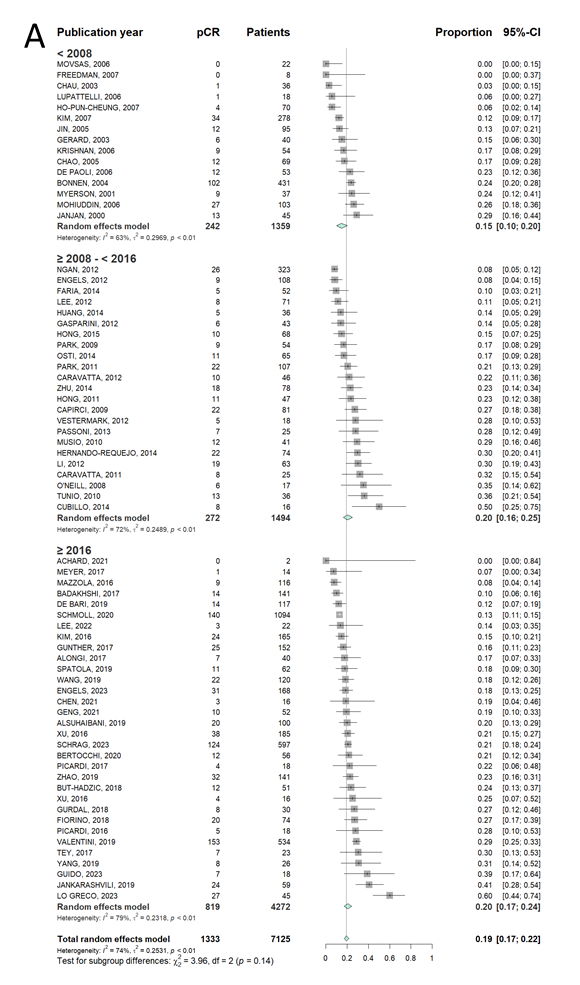


**Supplementary Figure 5:** Details of the meta-analysis of studies reporting pCR rate as function of (A) Publication year, (B) RT modulation, (C) CTV definition, (d) PTV definition, (E) boost sequence, (F) boost BED, (G) concomitant chemotherapy, (H) neo-adjuvant chemotherapy, and (I) Surgical delay.

The “pCR” column is the number of pCR events within each study, the “Patients” column reported the total of the patients within each study among the pooled publications, the “Proportion” column is the pCR rate within each study, the “95%-CI” column is the 95% confidence interval of the pCR rate within each study.

3D: Three-dimensional radiotherapy, BED: Biological equivalent dose, CTV: Clinical target volume, GTV: Gross tumour volume, IMRT: intensity-modulated radiotherapy, pCR: Pathologic complete response, PTV: Planning target volume, RT: Radiotherapy, SIB: simultaneous integrated boost, VMAT: Volumetric-modulated arc radiotherapy.


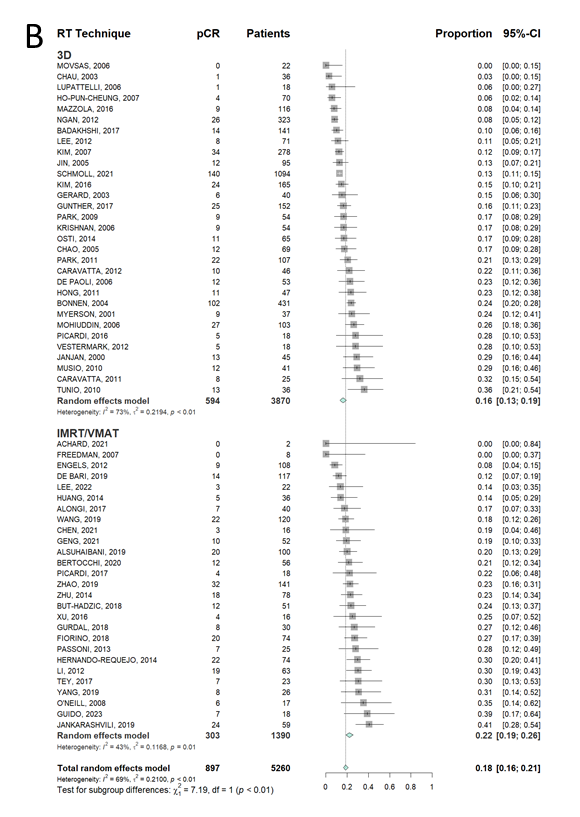


**Supplementary Figure 5 (continued)**


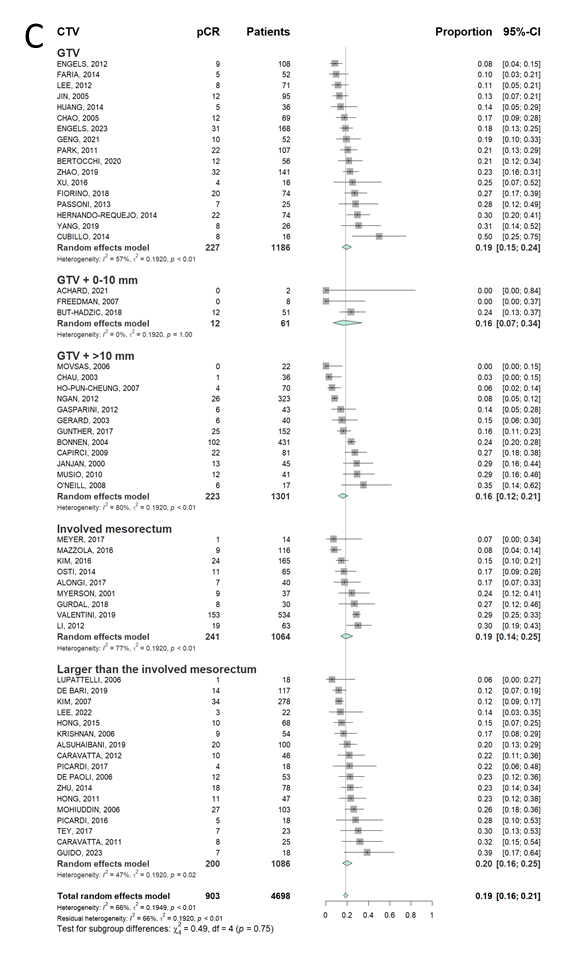


**Supplementary Figure 5 (continued)**


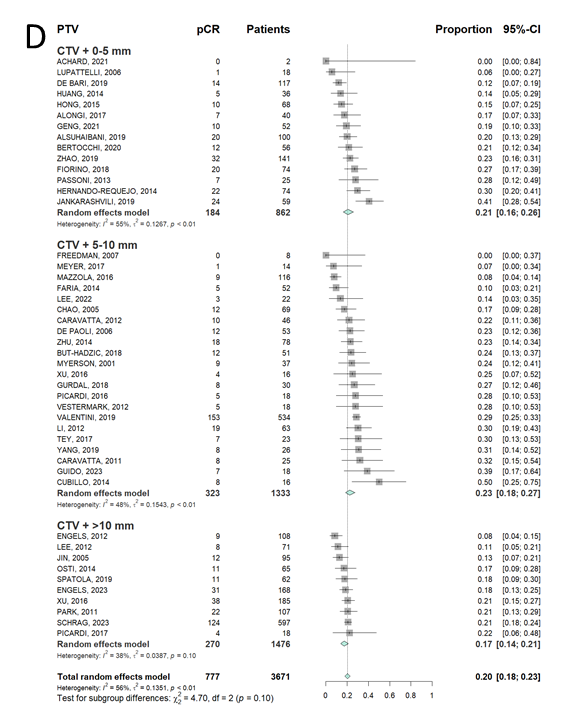


**Supplementary Figure 5 (continued)**


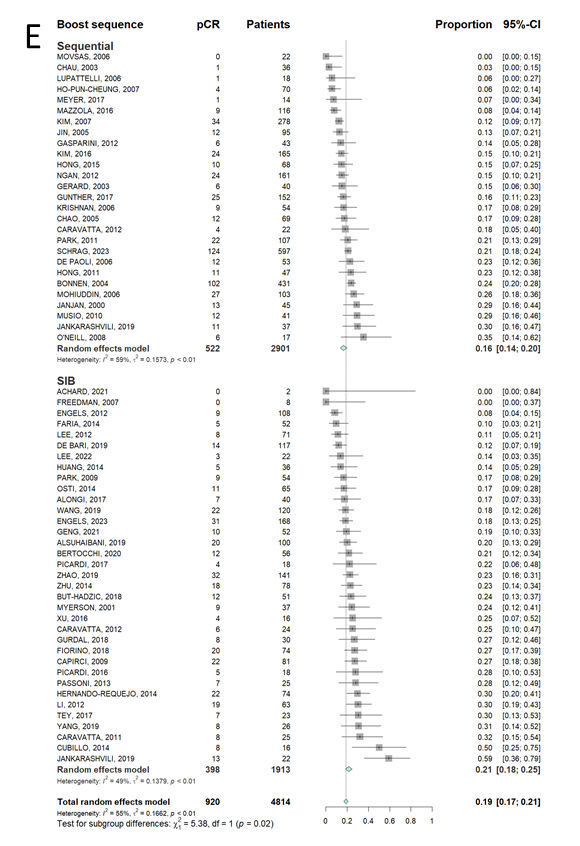


**Supplementary Figure 5 (continued)**


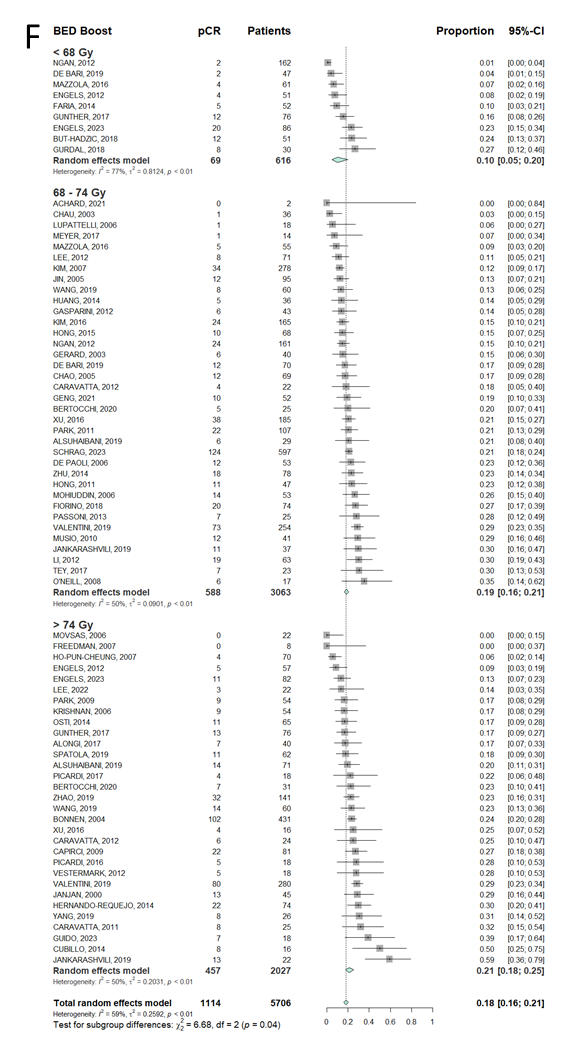


**Supplementary Figure 5 (continued)**


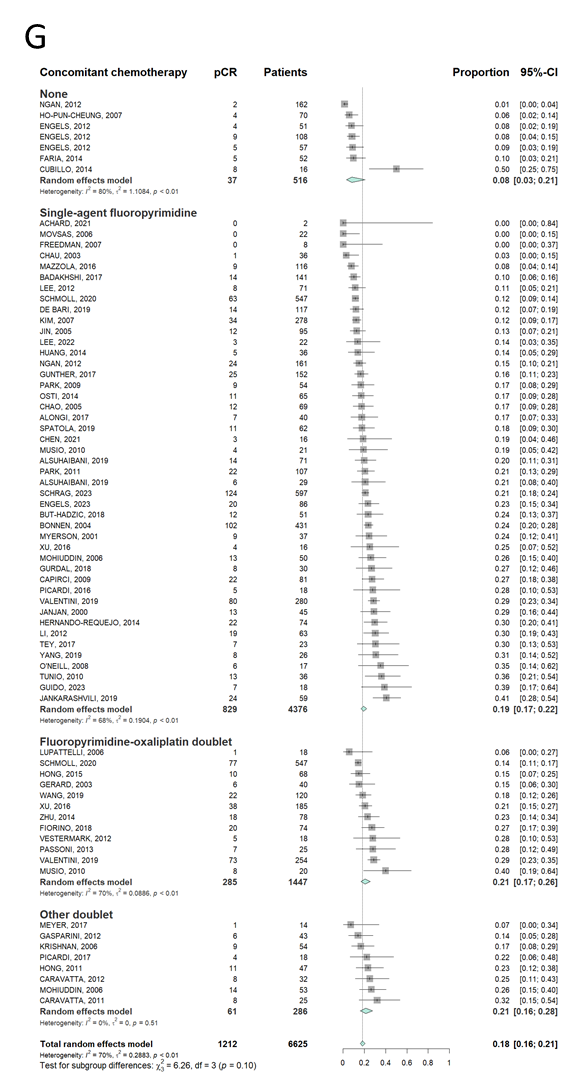


**Supplementary Figure 5 (continued)**


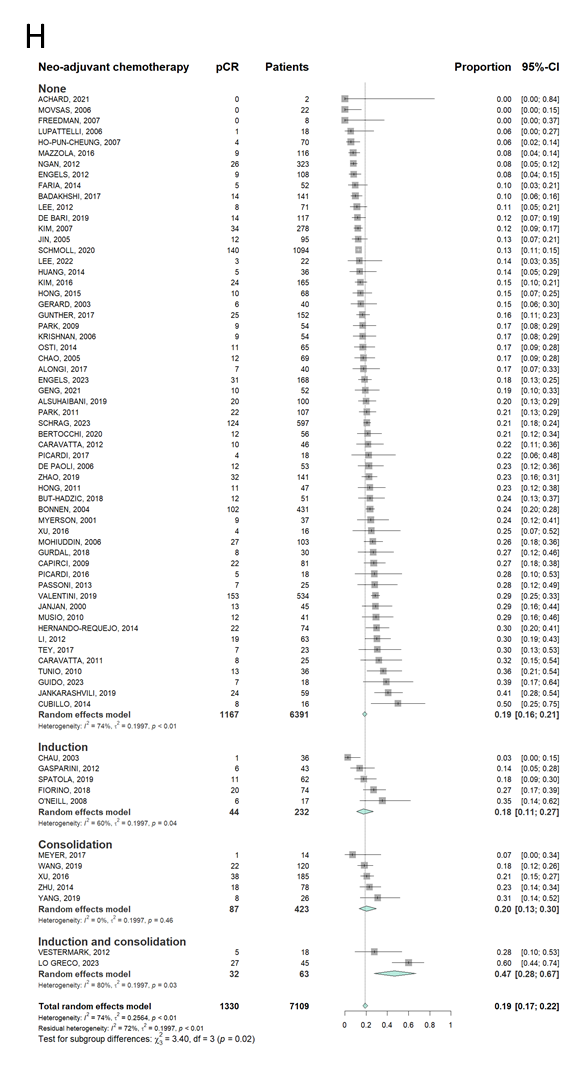


**Supplementary Figure 5 (continued)**


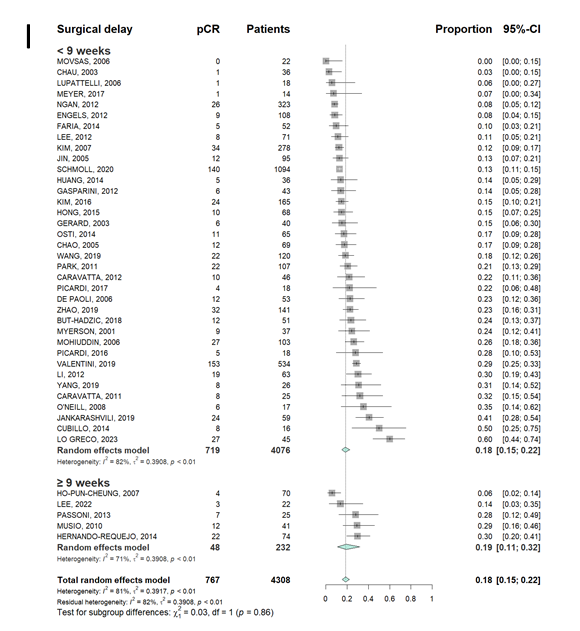


**Supplementary Figure 5 (continued)**


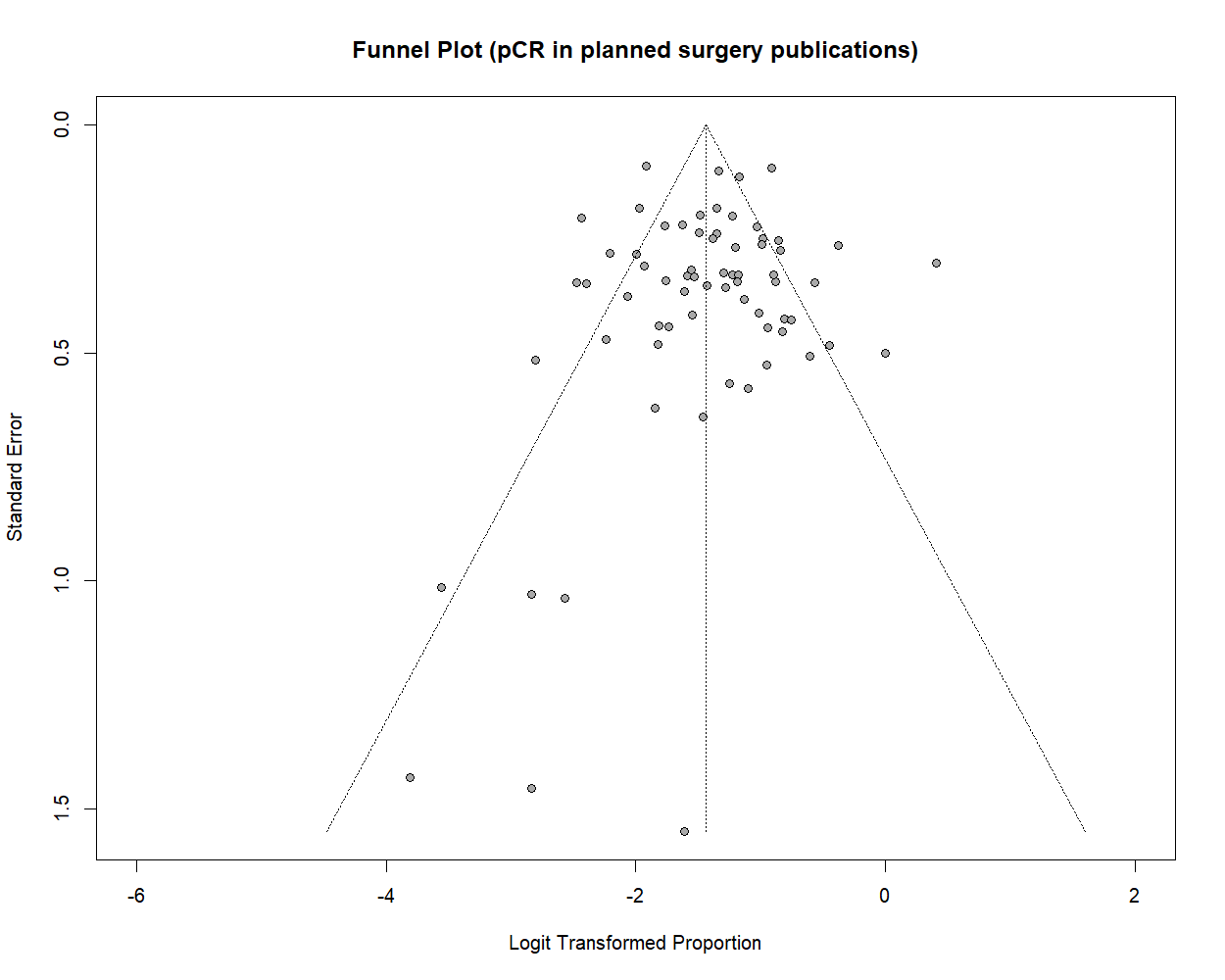


**Supplementary Figure 6:** Funnel plot of planned surgery publications reporting pCR.

pCR: Pathologic complete response.

**Supplementary Tables**

| **Class of data** | **Extracted characteristics** | **Categories** | | | | | | |
| --- | --- | --- | --- | --- | --- | --- | --- | --- |
| **Publication** | *Author* |  |  |  |  |  |  |  |
|  | *Year* | < 2008 | ≥ 2008 - < 2016 | ≥ 2016 |  |  |  |  |
|  | *Institution* |  |  |  |  |  |  |  |
|  | *Study design* | In silico | Protocol | Retrospective | Prospective |  |  |  |
| **RT technique** | *Modulation* | 3D | IMRT/VMAT | 3D/IMRT/VMAT | MRGRT | 3D/IMRT/PT |  |  |
|  | *Boost timing* | Sequential | Simultaneous | Combination of sequential or simultaneous |  |  |  |  |
|  | *Bifractionated* | No | Only for the boost | Whole treatment |  |  |  |  |
|  | *Adaptive RT for boost* | No | Yes |  |  |  |  |  |
| **RT preparation** | *Description of preparation* | No | Yes |  |  |  |  |  |
|  | *Oral preparation* | No contrast | Water as oral contrast | Oral contrast |  |  |  |  |
|  | *Rectum preparation* | No protocol | Empty rectum | Filling protocol | Rectal Gel | Rectal opacification |  |  |
|  | *Bladder preparation* | None | Comfortable full | Full | Water protocol | Baryum protocol | Other protocol |  |
|  | *Intravenous contrast* | No | Yes |  |  |  |  |  |
|  | *Treatment position* | Supine | Prone | Both |  |  |  |  |
| **RT boost delineation** | *Description of boost volume* | No | Yes |  |  |  |  |  |
|  | *Multi-modality imaging for GTV delineation* | None | DRE | Endoscopy | Ultrasonography | MRI | PET-CT |  |
|  | *Description of CTV* | No | Yes |  |  |  |  |  |
|  | *CTV margin* | CTV is GTV | Numeric margin | Anatomic margin | Numeric and anatomic margin |  |  |  |
|  | *Numeric CTV margin value* | 0 mm | > 0 – 10 mm | > 10 – 20 mm | Anisotropic |  |  |  |
|  | *Anatomic CTV margin* | None | Rectum involved | Mesorectum involved | Mesorectum and presacral space involved | Entire presacral space | Other |  |
|  | *Description of PTV* | No | None |  |  |  |  |  |
|  | *PTV is isotropic* | No | Yes |  |  |  |  |  |
|  | *PTV margin value** | 0 mm | > 0 – 5 mm | > 5 – 10 mm | > 10 – 15 mm | > 15 – 20 mm | > 20 – 30 mm |  |
| **RT dose** | *N fractions* | < 10 | 10 – 20 | > 20 |  |  |  |  |
|  | *BED elective* | < 68 Gy | 68 – 74 Gy | > 74 Gy |  |  |  |  |
|  | *BED boost* | < 68 Gy | 68 – 74 Gy | > 74 Gy |  |  |  |  |
| **Chemotherapy** | *Concomitant chemotherapy* | None | 5-fluorouracile | Capecitabine | 5-fluorouracile/Capecitabine + oxaliplatin | 5-fluorouracile/Capecitabine + another chemotherapy | 5-fluorouracile/Capecitabine + targeted therapy | Other |
|  | *Neo-adjuvant chemotherapy* | Induction | Consolidation | Induction and consolidation |  |  |  |  |
| **Follow-up** | *Follow-up strategy* | Scheduled surgery | Watch and wait |  |  |  |  |  |
|  | *Watch and wait management* | DRE | Endoscopy | MRI | PET-CT | CT | Ultrasonography |  |
|  | *Surgical delay* | < 9 weeks | ≥ 9 weeks |  |  |  |  |  |
|  | *Follow-up duration* | < 12 months | > 12 – 24 months | > 24 – 36 months | > 36 – 60 months | > 60 months |  |  |
| **Supplementary Table 1:** Rectal boost and treatment parameters extracted.  * In case of anisotropic PTV margin, the highest value is reported | | | | | | | | |

| **Score** | **Complete RT description** | **Correct terminology** | **Representativness of the selected cohort** | **Selection of the cohort** | **Ascertainment of exposure** | **Assessment of outcome** | **Follow-up long enough** | **Adequacy of follow up** |
| --- | --- | --- | --- | --- | --- | --- | --- | --- |
| **C** | Only dose is reported | Totally inaccurate | No locally advanced rectal cancer | No description | Not reported or >15% of patients not treated | No description | <1 year or not reported if local recurrence rate reported | No description of surgical delay and long-term follow-up |
| **B** | Incomplete RT description | Some inaccuracy | Combination of locally advanced and other rectal cancers | Cohorts from other institutions or database | Not all patients treated (up to 15%) | Different assessments between patients | 1-3 years if local recurrence rate reported | Incomplete description of surgical delay and long-term follow-up |
| **A** | Complete RT description (Including preparation, target volume description, multi-modality imaging use, RT modulation, prescribed dose, concomitant chemotherapy, and surgical delay | Accurate | Only locally advanced rectal cancer | Retrospective or prospective | All patients treated | One identical assesment for all patients | >3 years if local recurrence rate reported | Precise description of surgical delay and long-term follow-up |
| **Supplementary Table 2:** Modified Newcastle - Ottawa quality assessment scale for cohort studies. | | | | | | | | |

| **Author, year** | **Study design** | **RT modulation** | **Boost sequence** | **Position** | **CTV** | **PTV is Isotropic** | **Concomitant chemotherapy** | **Strategy** | **Surgical delay/ Evaluation of the cCR in W&W** |
| --- | --- | --- | --- | --- | --- | --- | --- | --- | --- |
| Achard, 2021 [1] | Retrospective | IMRT/VMAT | SIB | Supine | Numeric margin | Yes | Capecitabine | Planned surgery | Out of categories |
| Al-Khalidi, 2022 [2] | In silico | 3D | Sequential | Prone | Numeric and anatomic margin | Yes | - | - | - |
| Alsuhaibani, 2019 [3] | Retrospective | IMRT/VMAT | SIB | - | Numeric and anatomic margin | Yes | Capecitabine | Planned surgery | Out of categories |
| Anderson, 2007 [4] | In silico | 3D | Sequential | Prone | Numeric and anatomic margin | Yes | 5-fluorouracil | - | - |
| Appelt, 2015 [5] | Prospective | IMRT/VMAT | SIB | Supine | Anatomic margin | Yes | Other Singlet | W&W | 6 weeks |
| Arp, 2024 [6] | In silico | IMRT/VMAT | SIB | Supine | Anatomic margin | No | - | W&W | 6 – 12 weeks |
| Badakhshi, 2017 [7] | Retrospective | 3D | Sequential | Prone | - | - | 5-fluorouracil | Planned surgery | Out of categories |
| Bae, 2017 [8] | Retrospective | 3D/IMRT/VMAT | Sequential | Prone | Numeric and anatomic margin | Yes | 5-fluorouracil | Planned surgery | Out of categories |
| Bakkal, 2021 [9] | In silico | 3D/IMRT/VMAT | Sequential or SIB | Supine | Numeric margin | Yes | - | - | - |
| Ballonoff, 2008 [10] | Prospective | IMRT/VMAT | SIB | Prone | No margin | Yes | Capecitabine | Planned surgery | < 9 weeks |
| Bassi, 2008 [11] | In silico | - | - | Prone or supine | No margin | - | - | - | - |
| Bertocchi, 2020 [12] | Prospective | IMRT/VMAT | SIB | Supine | No margin | Yes | - | Planned surgery | Out of categories |
| Boeke, 2022 [13] | Prospective | MRI-guided RT | Sequential or SIB | - | No margin | Yes | None | W&W | 3 months |
| But-Hadzic, 2018 [14] | Prospective | IMRT/VMAT | SIB | - | Numeric margin | No | Capecitabine | Planned surgery | < 9 weeks |
| Capirci, 2009 [15] | Prospective | - | SIB | Prone | Numeric margin | - | 5-fluorouracil | Planned surgery | Out of categories |
| Chen, 2020 [16] | Retrospective | 3D/IMRT/VMAT | Sequential | Prone | - | No | 5-fluorouracil | Planned surgery | < 9 weeks |
| Chen, 2021 [17] | Retrospective | IMRT/VMAT | SIB | - | - | - | Capecitabine | Planned surgery | - |
| Chiloiro, 2023 [18] | Prospective | MRI-guided RT | SIB | - | No margin | Yes | 5-fluorouracil | W&W | 6 – 8 weeks |
| Colaco, 2014 [19] | In silico | 3D/IMRT/Protontherapy | Sequential | Prone | No margin | Yes | - | - | - |
| De Bari, 2019 [20] | Retrospective | IMRT/VMAT | SIB | - | Numeric and anatomic margin | Yes | Capecitabine | Planned surgery | Out of categories |
| De Paoli, 2006 [21] | Prospective | 3D | Sequential | Prone | Numeric and anatomic margin | Yes | Capecitabine | Planned surgery | < 9 weeks |
| Devlin, 2022 [22] | In silico | IMRT/VMAT | SIB | Supine | Numeric margin | Yes | - | - | - |
| Engels, 2023 [23] | Prospective | - | SIB | - | No margin | No | None | Planned surgery | Out of categories |
| Faria, 2014 [24] | Prospective | 3D/IMRT/VMAT | SIB | - | No margin | Yes | None | Planned surgery | < 9 weeks |
| Fiorino, 2018 [25] | Prospective | IMRT/VMAT | SIB | Supine | No margin | Yes | 5-fluorouracil-oxaliplatin doublet | W&W | - |
| Fleischmann, 2022 [26] | Protocol | IMRT/VMAT | Sequential | - | Numeric margin | - | Other-doublet | W&W | 10 weeks |
| Garant, 2022 [27] | Prospective | 3D | Sequential | - | Numeric margin | Yes | 5-fluorouracil | W&W | 13 weeks |
| Garcia-Aguilar, 2022 [28] | Prospective | 3D/IMRT/VMAT | Sequential or SIB | Prone or supine | Anatomic margin | No | 5-fluorouracil | W&W | 8 +/- 4 weeks |
| Gasparini, 2012 [29] | Prospective | - | Sequential | - | Numeric margin | - | Other-doublet | Planned surgery | < 9 weeks |
| Geng, 2021 [30] | Retrospective | IMRT/VMAT | SIB | Supine | No margin | Yes | Capecitabine | Planned surgery | Out of categories |
| **Supplementary Table 3:** List of publications included in the descriptive analysis.  3D: Three-dimensional radiotherapy, cCR: Clinical complete response, CTV: Clinical target volume, IMRT: intensity-modulated radiotherapy, MRI: Magnetic resonance imaging, PTV: Planning target volume, RT: Radiotherapy, SIB: simultaneous integrated boost, VMAT: Volumetric-modulated arc radiotherapy. | | | | | | | | | |
| Gerard, 2003 [31] | Prospective | 3D | Other | Prone | Numeric margin | - | 5-fluorouracil-oxaliplatin doublet | Planned surgery | < 9 weeks |
| Gerard, 2023 [32] | Prospective | 3D/IMRT/VMAT | Sequential | Prone or supine | No margin | Yes | Capecitabine | W&W | 24 weeks |
| Guido, 2023 [33] | Prospective | IMRT/VMAT | Sequential or SIB | Supine | Numeric and anatomic margin | Yes | Capecitabine | Planned surgery | Out of categories |
| Gunther, 2017 [34] | Retrospective | 3D | Other | Prone | Numeric margin | - | 5-fluorouracil | Planned surgery | Out of categories |
| Gurdal, 2018 [35] | Retrospective | IMRT/VMAT | SIB | Supine | Anatomic margin | Yes | 5-fluorouracil | Planned surgery | Out of categories |
| Hernando-Requejo, 2014 [36] | Retrospective | IMRT/VMAT | SIB | Prone | No margin | Yes | Capecitabine | Planned surgery | ≥ 9 weeks |
| Hong, 2011 [37] | Prospective | 3D | Sequential | - | Numeric and anatomic margin | - | Capectibanine-irinotecan doublet | Planned surgery | Out of categories |
| Ho-Pun-Cheung, 2007 [38] | Prospective | 3D | Sequential | Supine | Numeric margin | - | None | Planned surgery | ≥ 9 weeks |
| Huang, 2014 [39] | Prospective | IMRT/VMAT | SIB | - | No margin | Yes | Capecitabine | Planned surgery | < 9 weeks |
| Ingle, 2023 [40] | In silico | MRI-guided RT | Sequential | - | Numeric and anatomic margin | Yes | Capecitabine | - | - |
| Jabbour, 2012 [41] | Retrospective | 3D/IMRT/VMAT | Sequential | Prone or supine | Numeric and anatomic margin | Yes | 5-fluorouracil | Planned surgery | - |
| Jankarashvili, 2019 [42] | Retrospective | IMRT/VMAT | Sequential or SIB | Supine | Anatomic margin | Yes | Capecitabine | Planned surgery | < 9 weeks |
| Karaca, 2021 [43] | Retrospective | IMRT/VMAT | Sequential or SIB | Supine | Numeric and anatomic margin | Yes | 5-fluorouracil | W&W | - |
| Kilic, 2015 [44] | In silico | 3D/IMRT/VMAT | Sequential | Prone or supine | Numeric margin | Yes | - | - | - |
| Kim, 2016 [45] | Retrospective | 3D | Sequential | Prone | Anatomic margin | - | 5-fluorouracil | Planned surgery | < 9 weeks |
| Lee, 2022 [46] | Prospective | IMRT/VMAT | SIB | Supine | Numeric and anatomic margin | No | 5-fluorouracil | Planned surgery | ≥ 9 weeks |
| Liu, 2022 [47] | Prospective | IMRT/VMAT | Sequential | - | - | No | Capecitabine | Planned surgery | Out of categories |
| Lo Greco, 2023 [48] | Retrospective | 3D/IMRT/VMAT | Sequential | Prone | - | - | 5-fluorouracil | Planned surgery | < 9 weeks |
| Luna, 2019 [49] | In silico | 3D/IMRT/VMAT | Sequential | Prone | Numeric and anatomic margin | Yes | Capecitabine | - | - |
| Lupattelli, 2023 [50] | Retrospective | IMRT/VMAT | SIB | - | No margin | - | Capecitabine | W&W | - |
| Mazzola, 2016 [51] | Retrospective | 3D | Sequential | Prone | Anatomic margin | Yes | 5-fluorouracil | Planned surgery | Out of categories |
| Mohiuddin, 2006 [52] | Prospective | 3D | Sequential | - | Numeric and anatomic margin | - | 5-fluorouracil | Planned surgery | < 9 weeks |
| Movsas, 2006 [53] | Prospective | 3D | Sequential | Prone | Numeric margin | - | 5-fluorouracil | Planned surgery | < 9 weeks |
| Ngan, 2012 [54] | Prospective | 3D | Sequential | - | Numeric margin | - | None | Planned surgery | < 9 weeks |
| O'Neill, 2008 [55] | Retrospective | IMRT/VMAT | Sequential | Prone | Numeric margin | - | Capecitabine | Planned surgery | < 9 weeks |
| Osti, 2014 [56] | Prospective | 3D | SIB | - | Anatomic margin | No | Capecitabine | Planned surgery | < 9 weeks |
| Owens, 2020 [57] | Retrospective | IMRT/VMAT | SIB | Supine | Numeric margin | Yes | Capecitabine | W&W | 44 days |
| Parekh, 2013 [58] | Retrospective | 3D/IMRT/VMAT | Sequential or SIB | Prone | Numeric margin | Yes | 5-fluorouracil | Planned surgery | Out of categories |
| Park, 2011 [59] | Prospective | 3D | Sequential | - | No margin | No | Capecitabine | Planned surgery | < 9 weeks |
| Picardi, 2017 [60] | Prospective | IMRT/VMAT | SIB | Prone | Numeric and anatomic margin | No | Capecitabine-oxaliplatin doublet | Planned surgery | < 9 weeks |
| Radu, 2013 [61] | In silico | IMRT/VMAT | Sequential | - | Numeric margin | Yes | Capecitabine | - | - |
| Samuelian, 2012 [62] | Retrospective | 3D | Sequential | Prone or supine | No margin | Yes | 5-fluorouracil | Planned surgery | < 9 weeks |
| **Supplementary Table 3 (continued)** | | | | | | | | | |
| Schmoll, 2021 [63] | Prospective | 3D | Sequential | Prone or supine | - | - | Capecitabine | Planned surgery | < 9 weeks |
| Schrag, 2023 [64] | Prospective | 3D/IMRT/VMAT | Sequential | Prone or supine | Anatomic margin | No | 5-fluorouracil | Planned surgery | Out of categories |
| Seierstad, 2009 [65] | In silico | - | SIB | - | No margin | Yes | Capecitabine-oxaliplatin doublet | - | - |
| Shepherd, 2020 [66] | In silico | - | SIB | Supine | - | - | - | - | - |
| Spatola, 2019 [67] | Prospective | 3D/IMRT/VMAT | Other | Prone | - | Yes | 5-fluorouracil | Planned surgery | Out of categories |
| Temelli, 2019 [68] | In silico | IMRT/VMAT | SIB | Supine | - | Yes | - | - | - |
| Tepper, 2002 [69] | Prospective | 3D | Sequential | - | Numeric margin | - | 5-fluorouracil | Planned surgery | - |
| Tey, 2017 [70] | Prospective | IMRT/VMAT | SIB | Prone | No margin | Yes | Capecitabine | Planned surgery | Out of categories |
| Tunio, 2010 [71] | Prospective | 3D | Sequential | Prone | - | - | Capecitabine | Planned surgery | Out of categories |
| Verweij, 2023 [72] | Protocol | MRI-guided RT | Sequential | - | No margin | Yes | None | W&W | 11 – 13 weeks |
| Vestermark, 2012 [73] | Prospective | 3D | Sequential or SIB | Supine | - | Yes | Other-doublet | Planned surgery | Out of categories |
| Wawok, 2018 [74] | Prospective | 3D | Sequential | Prone | Anatomic margin | - | None | Planned surgery | < 9 weeks |
| Whaley, 2014 [75] | In silico | - | - | - | Anatomic margin | - | - | - | - |
| Xu, 2016 [76] | Prospective | IMRT/VMAT | SIB | Prone or supine | No margin | Yes | Capecitabine | Planned surgery | Out of categories |
| Yang, 2015 [77] | In silico | IMRT/VMAT | SIB | Supine | No margin | Yes | - | - | - |
| Yang, 2019 [78] | In silico | 3D | Sequential | - | - | - | 5-fluorouracil | - | - |
| Yang, 2019 [79] | Prospective | IMRT/VMAT | SIB | Supine | No margin | No | Capecitabine | Planned surgery | < 9 weeks |
| Zhang, 2019 [80] | Protocol | - | SIB | Supine | No margin | Yes | None | Planned surgery | ≥ 9 weeks |
| Zhao, 2016 [81] | In silico | 3D/IMRT/VMAT | SIB | Prone | No margin | Yes | - | - | - |
| Zhao, 2019 [82] | Prospective | IMRT/VMAT | SIB | Supine | No margin | Yes | Capecitabine | Planned surgery | < 9 weeks |
| Zhao, 2021 [83] | Retrospective | 3D | SIB | - | Anatomic margin | - | 5-fluorouracil | Planned surgery | - |
| **Supplementary Table 3 (continued)** | | | | | | | | | |

| **Institutional duplicates** | | | **Publications excluded through quality assessment analysis** |
| --- | --- | --- | --- |
| Allal, 2002 [84] | Freedman, 2007 [85] | Mohiuddin, 2013 [86] | Avancha, 2022 [87] |
| Allal, 2005 [88] | Gani, 2021 [89] | Musio, 2010 [90] | Avci, 2021 [91] |
| Alongi, 2017 [92] | Garcia-Aguilar, 2015 [93] | Myerson, 2001 [94] | Bitterman, 2015 [95] |
| Alvarez, 2024 [96] | Garcia-Aguilar, 2015 [97] | Myerson, 2002 [98] | Bountouroglou, 2004 [99] |
| Azria, 2005 [100] | Gerard, 2002 [101] | Park, 2009 [102] | Bruera, 2018 [103] |
| Bazarbashi, 2016 [104] | Gerard, 2015 [105] | Passoni, 2013 [106] | Bujko, 2013 [107] |
| Bird, 2021 [108] | Gevaert, 2012 [109] | Pfeiffer, 2005 [110] | Chan, 2000 [111] |
| Boldrini, 2018 [112] | Guillem, 2007 [113] | Picardi, 2016 [114] | Conroy, 2021 [115] |
| Bonnen, 2004 [116] | Habr-Gama, 2006 [117] | Pietsch, 2007 [118] | D'Amata, 2021 [119] |
| Bonomo, 2021 [120] | Habr-Gama, 2009 [121] | Raso, 2015 [122] | Datta, 2024 [123] |
| Braendengen, 2008 [124] | Habr-Gama, 2014 [125] | Roh, 2009 [126] | De Las Heras, 2013 [127] |
| Burbach, 2015 [128] | Habr-Gama, 2019 [129] | Rouanet, 2002 [130] | Di Tommaso, 2020 [131] |
| Burbach, 2016 [132] | Hong, 2015 [133] | Sanfilippo, 2001 [134] | Dumont, 2018 [135] |
| Capirci, 2002 [136] | Ingle, 2022 [137] | Sao Juliao, 2018 [138] | Garcia-Aguilar, 2011 [139] |
| Caravatta, 2011 [140] | Janjan, 2000 [141] | Tuta, 2019 [142] | Habr-Gama, 2013 [143] |
| Caravatta, 2012 [144] | Jeong, 2016 [145] | Urbano, 2006 [146] | Hashimoto, 2023 [147] |
| Chao, 2005 [148] | Jin, 2005 [149] | Valentini, 2001 [150] | Hudson, 2022 [151] |
| Chau, 2003 [152] | Kim, 2006 [153] | Valentini, 2008 [154] | Kennecke, 2012 [155] |
| Chiloiro, 2019 [156] | Kim, 2007 [157] | Valentini, 2019 [158] | Kim, 2002 [159] |
| Chiloiro, 2022 [160] | Klautke, 2005 [161] | Velenik, 2020 [162] | Klautke, 2007 [163] |
| Cilla, 2012 [164] | Kleijnen, 2019 [165] | Verweij, 2022 [166] | Markovina, 2017 [167] |
| Cilla, 2014 [168] | Krishnan, 2006 [169] | Vestermark, 2008 [170] | Mehta, 2001 [171] |
| Couwenberg, 2020 [172] | Lee, 2012 [173] | Wang, 2019 [174] | O'Connell, 2015 [175] |
| Cubillo, 2014 [176] | Lee, 2014 [177] | White, 2021 [178] | Ofshteyn, 2020 [179] |
| Czito, 2004 [180] | Li, 2012 [181] | Xu, 2014 [182] | Paszt, 2022 [183] |
| De Ridder, 2008 [184] | Li, 2021 [185] | Xu, 2017 [186] | Rodel, 2000 [187] |
| Dewdney, 2012 [188] | Loi, 2005 [189] | Yang, 2014 [190] | Schou, 2012 [191] |
| Eijkelenkamp, 2021 [192] | Lupattelli, 2006 [193] | Zhu, 2013 [194] | Singh, 2017 [195] |
| Elashwah, 2023 [196] | Lupattelli, 2017 [197] | Zhu, 2014 [198] | Sirak, 2020 [199] |
| Engels, 2012 [200] | Meyer, 2018 [201] |  | Wygoda, 2010 [202] |
| Engineer, 2013 [203] | Mohiuddin, 2000 [204] |  | Yamashita, 2017 [205] |
| Everaert, 2011 [206] | Mohiuddin, 2000 [207] |  | Yang, 2017 [208] |
| **Supplementary Table 4:** List of publications excluded from the descriptive analysis | | | |

| **Planned surgery publications** | | **Watch and wait publications** |
| --- | --- | --- |
| Allal, 2002 [84] | Liu, 2022 [47] | Bitterman, 2015 [95] |
| Allal, 2005 [88] | Loi, 2005 [189] | Habr-Gama, 2006 [117] |
| Avancha, 2022 [87] | Markovina, 2017 [167] | Habr-Gama, 2013 [143] |
| Avci, 2021 [91] | Mehta, 2001 [171] | Habr-Gama, 2014 [125] |
| Bae, 2017 [8] | Mohiuddin, 2000 [204] | Habr-Gama, 2019 [129] |
| Ballonoff, 2008 [10] | Mohiuddin, 2000 [207] | Sao Juliao, 2018 [138] |
| Bazarbashi, 2016 [104] | Mohiuddin, 2013 [86] |  |
| Braendengen, 2008 [124] | O'Connell, 2015 [175] |  |
| Bruera, 2018 [103] | Ofshteyn, 2020 [179] |  |
| Bujko, 2013 [107] | Parekh, 2013 [58] |  |
| Conroy, 2021 [115] | Rodel, 2000 [187] |  |
| Czito, 2004 [180] | Roh, 2009 [126] |  |
| De Las Heras, 2013 [127] | Rouanet, 2002 [130] |  |
| Dewdney, 2012 [188] | Samuelian, 2012 [62] |  |
| Di Tommaso, 2020 [131] | Sanfilippo, 2001 [134] |  |
| Dumont , 2018 [135] | Schou, 2012 [191] |  |
| Elashwah, 2023 [196] | Sirak, 2020 [199] |  |
| Engineer, 2013 [203] | Tuta, 2019 [142] |  |
| Garcia-Aguilar, 2011 [139] | Velenik, 2020 [162] |  |
| Garcia-Aguilar, 2015 [93] | Vestermark, 2008 [170] |  |
| Guillem, 2007 [113] | Wawok, 2018 [74] |  |
| Jabbour, 2012 [41] | Wygoda, 2010 [202] |  |
| Jeong, 2016 [145] | Xu, 2014 [209] |  |
| Kennecke, 2012 [155] | Yamashita, 2017 [205] |  |
| Kim, 2002 [159] | Yang, 2017 [208] |  |
| Klautke, 2007 [163] | Zhu, 2013 [194] |  |
| Lee, 2014 [177] |  |  |
| **Supplementary Table 5:** List of publications excluded from the meta-analysis through quality assessment analysis. | | |

| **Author, year** | **Publication year** | **RT Modulation** | **CTV** | **PTV** | **Boost sequence** | **Boost BED** | **Concomitant chemotherapy** | **Neo-adjuvant chemotherapy** | **Surgical delay** | **pCR reported** | **LRR reported** |
| --- | --- | --- | --- | --- | --- | --- | --- | --- | --- | --- | --- |
| Achard, 2021 [1] | ≥ 2016 | IMRT/VMAT | GTV + 0-10 mm | CTV + 0-5 mm | SIB | 68 - 74 Gy | Single-agent fluoropyrimidine | None | OUT | Yes | Yes |
| Alongi, 2017 [92] | ≥ 2016 | IMRT/VMAT | Involved mesorectum | CTV + 0-5 mm | SIB | > 74 Gy | Single-agent fluoropyrimidine | None | OUT | Yes | No |
| Alsuhaibani, 2019 [3] | ≥ 2016 | IMRT/VMAT | More than involved mesorectum | CTV + 0-5 mm | SIB | 68 - 74 Gy | Single-agent fluoropyrimidine | None | OUT | Yes | No |
|  | ≥ 2016 | IMRT/VMAT | More than involved mesorectum | CTV + 0-5 mm | SIB | > 74 Gy | Single-agent fluoropyrimidine | None | OUT | Yes | No |
| Badakhshi, 2017 [7] | ≥ 2016 | 3D | OUT | OUT | OUT | OUT | Single-agent fluoropyrimidine | None | OUT | Yes | No |
| Bertocchi, 2020 [12] | ≥ 2016 | IMRT/VMAT | GTV | CTV + 0-5 mm | SIB | 68 - 74 Gy | OUT | None | OUT | Yes | No |
|  | ≥ 2016 | IMRT/VMAT | GTV | CTV + 0-5 mm | SIB | > 74 Gy | OUT | None | OUT | Yes | No |
| Bonnen, 2004 [116] | < 2008 | 3D | GTV + > 10 mm | OUT | Sequential | > 74 Gy | Single-agent fluoropyrimidine | None | OUT | Yes | No |
| But-Hadzic, 2018 [14] | ≥ 2016 | IMRT/VMAT | GTV + 0-10 mm | CTV + 5-10 mm | SIB | < 68 Gy | Single-agent fluoropyrimidine | None | < 9 weeks | Yes | Yes |
| Capirci, 2009 [15] | ≥ 2008 - < 2016 | OUT | GTV + > 10 mm | OUT | SIB | > 74 Gy | Single-agent fluoropyrimidine | None | OUT | Yes | No |
| Caravatta, 2011 [140] | ≥ 2008 - < 2016 | 3D | More than involved mesorectum | CTV + 5-10 mm | SIB | > 74 Gy | Other doublet | None | < 9 weeks | Yes | Yes |
| Caravatta, 2012 [144] | ≥ 2008 - < 2016 | 3D | More than involved mesorectum | CTV + 5-10 mm | Sequential | 68 - 74 Gy | OUT | None | < 9 weeks | Yes | Yes |
|  | ≥ 2008 - < 2016 | 3D | More than involved mesorectum | CTV + 5-10 mm | SIB | > 74 Gy | OUT | None | < 9 weeks | Yes | Yes |
|  | ≥ 2008 - < 2016 | 3D | More than involved mesorectum | CTV + 5-10 mm | OUT | OUT | Other doublet | None | < 9 weeks | Yes | Yes |
| Chao, 2005 [148] | < 2008 | 3D | GTV | CTV + 5-10 mm | Sequential | 68 - 74 Gy | Single-agent fluoropyrimidine | None | < 9 weeks | Yes | Yes |
| Chau, 2003 [152] | < 2008 | 3D | GTV + > 10 mm | OUT | Sequential | 68 - 74 Gy | Single-agent fluoropyrimidine | Induction | < 9 weeks | Yes | Yes |
| Chen, 2021 [17] | ≥ 2016 | IMRT/VMAT | OUT | OUT | OUT | OUT | Single-agent fluoropyrimidine | OUT | OUT | Yes | Yes |
| Cubillo, 2014 [176] | ≥ 2008 - < 2016 | OUT | GTV | CTV + 5-10 mm | SIB | > 74 Gy | None | None | < 9 weeks | Yes | No |
| De Bari, 2019 [20] | ≥ 2016 | IMRT/VMAT | More than involved mesorectum | CTV + 0-5 mm | SIB | < 68 Gy | Single-agent fluoropyrimidine | None | OUT | Yes | No |
|  | ≥ 2016 | IMRT/VMAT | More than involved mesorectum | CTV + 0-5 mm | SIB | 68 - 74 Gy | Single-agent fluoropyrimidine | None | OUT | Yes | No |
| De Paoli, 2006 [21] | < 2008 | 3D | More than involved mesorectum | CTV + 5-10 mm | Sequential | 68 - 74 Gy | OUT | None | < 9 weeks | Yes | No |
| Engels, 2012 [200] | ≥ 2008 - < 2016 | IMRT/VMAT | GTV | CTV + > 10 mm | SIB | < 68 Gy | None | None | < 9 weeks | Yes | Yes |
|  | ≥ 2008 - < 2016 | IMRT/VMAT | GTV | CTV + > 10 mm | SIB | > 74 Gy | None | None | < 9 weeks | Yes | Yes |
| Engels, 2023 [23] | ≥ 2016 | OUT | GTV | CTV + > 10 mm | SIB | < 68 Gy | Single-agent fluoropyrimidine | None | OUT | Yes | Yes |
|  | ≥ 2016 | OUT | GTV | CTV + > 10 mm | SIB | > 74 Gy | OUT | None | OUT | Yes | Yes |
| Faria, 2014 [24] | ≥ 2008 - < 2016 | OUT | GTV | CTV + 5-10 mm | SIB | < 68 Gy | None | None | < 9 weeks | Yes | Yes |
| Fiorino, 2018 [25] | ≥ 2016 | IMRT/VMAT | GTV | CTV + 0-5 mm | SIB | 68 - 74 Gy | Fluoropyrimidine-oxaliplatin doublet | Induction | OUT | Yes | No |
| Freedman, 2007 [85] | < 2008 | IMRT/VMAT | GTV + 0-10 mm | CTV + 5-10 mm | SIB | > 74 Gy | Single-agent fluoropyrimidine | None | OUT | Yes | Yes |
| **Supplementary Table 6:** List of included publications in the meta-analysis for planned surgery cohorts.  3D: Three-dimensional radiotherapy, BED: Biological equivalent dose, CTV: Clinical target volume, GTV: Gross tumour volume, IMRT: intensity-modulated radiotherapy, LRR: Local recurrence rate, OUT: Out of category, pCR: Pathological complete response, PTV: Planning target volume, RT: Radiotherapy, SIB: simultaneous integrated boost, VMAT: Volumetric-modulated arc radiotherapy. | | | | | | | | | | | |
| Gasparini, 2012 [29] | ≥ 2008 - < 2016 | OUT | GTV + > 10 mm | OUT | Sequential | 68 - 74 Gy | Other doublet | Induction | < 9 weeks | Yes | Yes |
| Geng, 2021 [30] | ≥ 2016 | IMRT/VMAT | GTV | CTV + 0-5 mm | SIB | 68 - 74 Gy | OUT | None | OUT | Yes | Yes |
| Gerard, 2003 [31] | < 2008 | 3D | GTV + > 10 mm | OUT | Sequential | 68 - 74 Gy | Fluoropyrimidine-oxaliplatin doublet | None | < 9 weeks | Yes | Yes |
| Guido, 2023 [33] | ≥ 2016 | IMRT/VMAT | More than involved mesorectum | CTV + 5-10 mm | OUT | > 74 Gy | Single-agent fluoropyrimidine | None | OUT | Yes | Yes |
| Gunther, 2017 [34] | ≥ 2016 | 3D | GTV + > 10 mm | OUT | Sequential | < 68 Gy | Single-agent fluoropyrimidine | None | OUT | Yes | No |
|  | ≥ 2016 | 3D | GTV + > 10 mm | OUT | Sequential | > 74 Gy | Single-agent fluoropyrimidine | None | OUT | Yes | No |
| Gurdal, 2018 [35] | ≥ 2016 | IMRT/VMAT | Involved mesorectum | CTV + 5-10 mm | SIB | < 68 Gy | Single-agent fluoropyrimidine | None | OUT | Yes | Yes |
| Hernando-Requejo, 2014 [36] | ≥ 2008 - < 2016 | IMRT/VMAT | GTV | CTV + 0-5 mm | SIB | > 74 Gy | Single-agent fluoropyrimidine | None | ≥ 9 weeks | Yes | Yes |
| Hong, 2011 [37] | ≥ 2008 - < 2016 | 3D | More than involved mesorectum | OUT | Sequential | 68 - 74 Gy | Other doublet | None | OUT | Yes | Yes |
| Hong, 2015 [133] | ≥ 2008 - < 2016 | OUT | More than involved mesorectum | CTV + 0-5 mm | Sequential | 68 - 74 Gy | Fluoropyrimidine-oxaliplatin doublet | None | < 9 weeks | Yes | Yes |
| Ho-Pun-Cheung, 2007 [38] | < 2008 | 3D | GTV + > 10 mm | OUT | Sequential | > 74 Gy | None | None | ≥ 9 weeks | Yes | Yes |
| Huang, 2014 [39] | ≥ 2008 - < 2016 | IMRT/VMAT | GTV | CTV + 0-5 mm | SIB | 68 - 74 Gy | Single-agent fluoropyrimidine | None | < 9 weeks | Yes | Yes |
| Janjan, 2000 [141] | < 2008 | 3D | GTV + > 10 mm | OUT | Sequential | > 74 Gy | Single-agent fluoropyrimidine | None | OUT | Yes | No |
| Jankarashvili, 2019 [42] | ≥ 2016 | IMRT/VMAT | OUT | CTV + 0-5 mm | Sequential | 68 - 74 Gy | Single-agent fluoropyrimidine | None | < 9 weeks | Yes | No |
|  | ≥ 2016 | IMRT/VMAT | OUT | CTV + 0-5 mm | SIB | > 74 Gy | Single-agent fluoropyrimidine | None | < 9 weeks | Yes | No |
| Jin, 2005 [149] | < 2008 | 3D | GTV | CTV + > 10 mm | Sequential | 68 - 74 Gy | Single-agent fluoropyrimidine | None | < 9 weeks | Yes | No |
| Kim, 2016 [45] | ≥ 2016 | 3D | Involved mesorectum | OUT | Sequential | 68 - 74 Gy | OUT | None | < 9 weeks | Yes | Yes |
| Krishnan, 2006 [169] | < 2008 | 3D | More than involved mesorectum | OUT | Sequential | > 74 Gy | Other doublet | None | OUT | Yes | Yes |
| Lee, 2012 [173] | ≥ 2008 - < 2016 | 3D | GTV | CTV + > 10 mm | SIB | 68 - 74 Gy | Single-agent fluoropyrimidine | None | < 9 weeks | Yes | No |
| Lee, 2022 [46] | ≥ 2016 | IMRT/VMAT | More than involved mesorectum | CTV + 5-10 mm | SIB | > 74 Gy | Single-agent fluoropyrimidine | None | ≥ 9 weeks | Yes | Yes |
| Li, 2012 [181] | ≥ 2008 - < 2016 | IMRT/VMAT | Involved mesorectum | CTV + 5-10 mm | SIB | 68 - 74 Gy | Single-agent fluoropyrimidine | None | < 9 weeks | Yes | Yes |
| Lo Greco, 2023 [48] | ≥ 2016 | OUT | OUT | OUT | OUT | OUT | OUT | Induction and consolidation | < 9 weeks | Yes | Yes |
| Lupattelli, 2006 [193] | < 2008 | 3D | More than involved mesorectum | CTV + 0-5 mm | Sequential | 68 - 74 Gy | Fluoropyrimidine-oxaliplatin doublet | None | < 9 weeks | Yes | Yes |
| Mazzola, 2016 [51] | ≥ 2016 | 3D | Involved mesorectum | CTV + 5-10 mm | Sequential | < 68 Gy | Single-agent fluoropyrimidine | None | OUT | Yes | No |
|  | ≥ 2016 | 3D | Involved mesorectum | CTV + 5-10 mm | Sequential | 68 - 74 Gy | Single-agent fluoropyrimidine | None | OUT | Yes | No |
| Meyer, 2017 [201] | ≥ 2016 | OUT | Involved mesorectum | CTV + 5-10 mm | Sequential | 68 - 74 Gy | Other doublet | Consolidation | < 9 weeks | Yes | Yes |
| Mohiuddin, 2006 [52] | < 2008 | 3D | More than involved mesorectum | OUT | Sequential | OUT | Single-agent fluoropyrimidine | None | < 9 weeks | Yes | No |
|  | < 2008 | 3D | More than involved mesorectum | OUT | Sequential | 68 - 74 Gy | Other doublet | None | < 9 weeks | Yes | No |
| Movsas, 2006 [53] | < 2008 | 3D | GTV + > 10 mm | OUT | Sequential | > 74 Gy | Single-agent fluoropyrimidine | None | < 9 weeks | Yes | Yes |
| **Supplementary Table 6 (continued)** | | | | | | | | | | | |
|  | | | | | | | | | | | |
| Musio, 2010 [90] | ≥ 2008 - < 2016 | 3D | GTV + > 10 mm | OUT | Sequential | 68 - 74 Gy | Single-agent fluoropyrimidine | None | ≥ 9 weeks | Yes | No |
|  | ≥ 2008 - < 2016 | 3D | GTV + > 10 mm | OUT | Sequential | 68 - 74 Gy | Fluoropyrimidine-oxaliplatin doublet | None | ≥ 9 weeks | Yes | No |
| Myerson, 2001 [94] | < 2008 | 3D | Involved mesorectum | CTV + 5-10 mm | SIB | OUT | Single-agent fluoropyrimidine | None | < 9 weeks | Yes | No |
| Ngan, 2012 [54] | ≥ 2008 - < 2016 | 3D | GTV + > 10 mm | OUT | OUT | < 68 Gy | None | None | < 9 weeks | Yes | Yes |
|  | ≥ 2008 - < 2016 | 3D | GTV + > 10 mm | OUT | Sequential | 68 - 74 Gy | Single-agent fluoropyrimidine | None | < 9 weeks | Yes | Yes |
| O'Neill, 2008 [55] | ≥ 2008 - < 2016 | IMRT/VMAT | GTV + > 10 mm | OUT | Sequential | 68 - 74 Gy | Single-agent fluoropyrimidine | Induction | < 9 weeks | Yes | No |
| Osti, 2014 [56] | ≥ 2008 - < 2016 | 3D | Involved mesorectum | CTV + > 10 mm | SIB | > 74 Gy | Single-agent fluoropyrimidine | None | < 9 weeks | Yes | Yes |
| Park, 2009 [102] | ≥ 2008 - < 2016 | 3D | OUT | OUT | SIB | > 74 Gy | Single-agent fluoropyrimidine | None | OUT | Yes | Yes |
| Park, 2011 [59] | ≥ 2008 - < 2016 | 3D | GTV | CTV + > 10 mm | Sequential | 68 - 74 Gy | Single-agent fluoropyrimidine | None | < 9 weeks | Yes | Yes |
| Passoni, 2013 [106] | ≥ 2008 - < 2016 | IMRT/VMAT | GTV | CTV + 0-5 mm | SIB | 68 - 74 Gy | Fluoropyrimidine-oxaliplatin doublet | None | ≥ 9 weeks | Yes | No |
| Picardi, 2016 [114] | ≥ 2016 | 3D | More than involved mesorectum | CTV + 5-10 mm | SIB | > 74 Gy | Single-agent fluoropyrimidine | None | < 9 weeks | Yes | No |
| Picardi, 2017 [60] | ≥ 2016 | IMRT/VMAT | More than involved mesorectum | CTV + > 10 mm | SIB | > 74 Gy | Other doublet | None | < 9 weeks | Yes | Yes |
| Schmoll, 2020 [63] | ≥ 2016 | 3D | OUT | OUT | OUT | OUT | Single-agent fluoropyrimidine | None | < 9 weeks | Yes | Yes |
|  | ≥ 2016 | 3D | OUT | OUT | OUT | OUT | Fluoropyrimidine-oxaliplatin doublet | None | < 9 weeks | Yes | Yes |
| Schrag, 2023 [64] | ≥ 2016 | OUT | OUT | CTV + > 10 mm | Sequential | 68 - 74 Gy | Single-agent fluoropyrimidine | None | OUT | Yes | Yes |
| Spatola, 2019 [67] | ≥ 2016 | OUT | OUT | CTV + > 10 mm | OUT | > 74 Gy | Single-agent fluoropyrimidine | Induction | OUT | Yes | Yes |
| Tey, 2017 [70] | ≥ 2016 | IMRT/VMAT | More than involved mesorectum | CTV + 5-10 mm | SIB | 68 - 74 Gy | Single-agent fluoropyrimidine | None | OUT | Yes | Yes |
| Tunio, 2010 [71] | ≥ 2008 - < 2016 | 3D | OUT | OUT | OUT | OUT | Single-agent fluoropyrimidine | None | OUT | Yes | No |
| Valentini, 2019 [158] | ≥ 2016 | OUT | Involved mesorectum | CTV + 5-10 mm | OUT | > 74 Gy | Single-agent fluoropyrimidine | None | < 9 weeks | Yes | No |
|  | ≥ 2016 | OUT | Involved mesorectum | CTV + 5-10 mm | OUT | 68 - 74 Gy | Fluoropyrimidine-oxaliplatin doublet | None | < 9 weeks | Yes | No |
| Vestermark, 2012 [73] | ≥ 2008 - < 2016 | 3D | OUT | CTV + 5-10 mm | OUT | > 74 Gy | Fluoropyrimidine-oxaliplatin doublet | Induction and consolidation | OUT | Yes | No |
| Wang, 2019 [174] | ≥ 2016 | IMRT/VMAT | OUT | OUT | SIB | 68 - 74 Gy | Fluoropyrimidine-oxaliplatin doublet | Consolidation | < 9 weeks | Yes | No |
|  | ≥ 2016 | IMRT/VMAT | OUT | OUT | SIB | > 74 Gy | Fluoropyrimidine-oxaliplatin doublet | Consolidation | < 9 weeks | Yes | No |
| Xu, 2017 [186] | ≥ 2016 | OUT | OUT | CTV + > 10 mm | OUT | 68 - 74 Gy | Fluoropyrimidine-oxaliplatin doublet | Consolidation | OUT | Yes | No |
| Xu, 2016 [76] | ≥ 2016 | IMRT/VMAT | GTV | CTV + 5-10 mm | SIB | > 74 Gy | Single-agent fluoropyrimidine | None | OUT | Yes | No |
| Yang, 2019 [79] | ≥ 2016 | IMRT/VMAT | GTV | CTV + 5-10 mm | SIB | > 74 Gy | Single-agent fluoropyrimidine | Consolidation | < 9 weeks | Yes | No |
| Zhao, 2019 [82] | ≥ 2016 | IMRT/VMAT | GTV | CTV + 0-5 mm | SIB | > 74 Gy | OUT | None | < 9 weeks | Yes | Yes |
| Zhu, 2014 [198] | ≥ 2008 - < 2016 | IMRT/VMAT | More than involved mesorectum | CTV + 5-10 mm | SIB | 68 - 74 Gy | Fluoropyrimidine-oxaliplatin doublet | Consolidation | OUT | Yes | Yes |
| **Supplementary Table 6 (continued)** | | | | | | | | | | | |

| **Author, year** | **Publication year** | **RT Modulation** | **CTV** | **PTV** | **Boost sequence** | **Boost BED** | **Concomitant chemotherapy** | **Neo-adjuvant chemotherapy** | **cCR reported** | **LRR reported** |
| --- | --- | --- | --- | --- | --- | --- | --- | --- | --- | --- |
| Boeke, 2022 [13] | ≥ 2016 | OUT | GTV | CTV + 5-10 mm | OUT | > 74 Gy | None | None | Yes | Yes |
| Chiloiro, 2019 [156] | ≥ 2016 | OUT | Involved mesorectum | CTV + 0-5 mm | SIB | > 74 Gy | OUT | OUT | Yes | No |
| Couwenberg, 2020 [172] | ≥ 2016 | IMRT/VMAT | GTV | CTV + > 10 mm | Sequential | 68 - 74 Gy | Single-agent fluoropyrimidine | None | Yes | Yes |
|  | ≥ 2016 | IMRT/VMAT | GTV | CTV + > 10 mm | Sequential | > 74 Gy | Single-agent fluoropyrimidine | None | Yes | Yes |
| Garant, 2022 [27] | ≥ 2016 | 3D | GTV + > 10 mm | CTV + 5-10 mm | Sequential | 68 - 74 Gy | Single-agent fluoropyrimidine | None | Yes | No |
| Garcia-Aguilar, 2022 [28] | ≥ 2016 | OUT | OUT | CTV + > 10 mm | OUT | OUT | Single-agent fluoropyrimidine | Induction | Yes | Yes |
|  | ≥ 2016 | OUT | OUT | CTV + > 10 mm | OUT | OUT | Single-agent fluoropyrimidine | Consolidation | Yes | Yes |
| Gerard, 2023 [32] | ≥ 2016 | OUT | GTV | CTV + > 10 mm | Sequential | 68 - 74 Gy | Single-agent fluoropyrimidine | None | Yes | Yes |
| Lupattelli, 2023 [50] | ≥ 2016 | IMRT/VMAT | GTV | OUT | SIB | > 74 Gy | Single-agent fluoropyrimidine | None | Yes | Yes |
| Owens, 2020 [57] | ≥ 2016 | IMRT/VMAT | GTV + 0-10 mm | CTV + > 10 mm | SIB | 68 - 74 Gy | Single-agent fluoropyrimidine | None | Yes | No |
| **Supplementary Table 7:** List of included publications in the meta-analysis for W&W cohorts.  3D: Three-dimensional radiotherapy, BED: Biological equivalent dose, cCR: Clinical complete response, CTV: Clinical target volume, GTV: Gross tumour volume, IMRT: intensity-modulated radiotherapy, LRR: Local recurrence rate, OUT: Out of category, PTV: Planning target volume, RT: Radiotherapy, SIB: simultaneous integrated boost, VMAT: Volumetric-modulated arc radiotherapy, W&W: Watch and wait. | | | | | | | | | | |


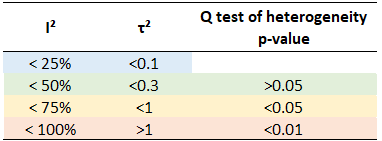


| **Meta-analysis of pCR**  **in planned surgery publications** | **I²** | **τ²** | **Q test of heterogeneity p-value** |
| --- | --- | --- | --- |
| **Publication year** | **74%** | **0.25** | **< 0.01** |
| < 2008 | 63% | 0.30 | < 0.01 |
| ≥ 2008 - < 2016 | 72% | 0.25 | < 0.01 |
| ≥ 2016 | 79% | 0.23 | < 0.01 |
| **RT Modulation** | **69%** | **0.21** | **< 0.01** |
| 3D | 73% | 0.22 | < 0.01 |
| IMRT/VMAT | 43% | 0.12 | 0.01 |
| **CTV** | **66%** | **0.19** | **< 0.01** |
| GTV | 57% | 0.19 | < 0.01 |
| GTV + 0-10 mm | 0% | 0.19 | 1.00 |
| GTV + > 10 mm | 80% | 0.19 | < 0.01 |
| Involved mesorectum | 77% | 0.19 | < 0.01 |
| Larger than involved mesorectum | 47% | 0.19 | 0.02 |
| **PTV** | **56%** | **0.14** | **< 0.01** |
| CTV + 0-5 mm | 55% | 0.13 | < 0.01 |
| CTV + 5-10 mm | 48% | 0.15 | < 0.01 |
| CTV + > 10 mm | 38% | 0.04 | 0.1 |
| **Boost sequence** | **55%** | **0.17** | **< 0.01** |
| Sequential | 59% | 0.16 | < 0.01 |
| SIB | 49% | 0.14 | < 0.01 |
| **Boost BED** | **59%** | **0.26** | **< 0.01** |
| < 68 Gy | 77% | 0.81 | < 0.01 |
| 68 - 74 Gy | 50% | 0.09 | < 0.01 |
| > 74 Gy | 50% | 0.20 | < 0.01 |
| **Concomitant chemotherapy** | **70%** | **0.29** | **< 0.01** |
| None | 80% | 1.11 | < 0.01 |
| Single-agent fluoropyrimidine | 68% | 0.19 | < 0.01 |
| Fluoropyrimidine-oxaliplatin doublet | 70% | 0.09 | < 0.01 |
| Other doublet | 0% | 0.00 | 0.51 |
| **Neo-adjuvant chemotherapy** | **74%** | **0.26** | **< 0.01** |
| None | 74% | 0.20 | < 0.01 |
| Induction | 60% | 0.20 | 0.04 |
| Consolidation | 0% | 0.20 | 0.46 |
| Induction and consolidation | 80% | 0.20 | 0.03 |
| **Surgical delay** | **81%** | **0.39** | **< 0.01** |
| < 9 weeks | 82% | 0.39 | < 0.01 |
| ≥ 9 weeks | 71% | 0.39 | < 0.01 |
| **Supplementary Table 8:** Heterogeneity analysis of the pCR meta-analysis of planned surgery publications.  3D: Three-dimensional radiotherapy, BED: Biological equivalent dose, CTV: Clinical target volume, GTV: Gross tumour volume, IMRT: intensity-modulated radiotherapy, pCR: Pathologic complete response, PTV: Planning target volume, RT: Radiotherapy, SIB: simultaneous integrated boost, VMAT: Volumetric-modulated arc radiotherapy. | | | |

**References**

1. Achard, V., et al., *Sexual organ-sparing with hydrogel spacer injections for rectal cancer radiotherapy: a feasibility pilot study.* Br J Radiol, 2021. **94**(1120): p. 20200931.

2. Al-Khalidi, R.E.H. and N.K. Abdulkareem, *Evaluation of Three-Dimensional Conformal Radiation Therapy in Rectal Cancer.* NeuroQuantology, 2022. **20**(8): p. 310-316.

3. Alsuhaibani, A., et al., *Dose Escalation with Simultaneous Integrated Boost (SIB) Using Volumetric Modulated Arc Therapy (VMAT) in Rectal Cancer.* Journal of Gastrointestinal Cancer, 2019. **50**(4): p. 735-739.

4. Anderson, C., et al., *PET-CT fusion in radiation management of patients with anorectal tumors.* Int J Radiat Oncol Biol Phys, 2007. **69**(1): p. 155-62.

5. Appelt, A.L., et al., *High-dose chemoradiotherapy and watchful waiting for distal rectal cancer: a prospective observational study.* Lancet Oncol, 2015. **16**(8): p. 919-27.

6. Arp, D.T., et al., *Treatment planning for patients with low rectal cancer in a multicenter prospective organ preservation study.* Physica Medica, 2024. **118**.

7. Badakhshi, H., et al., *The role of concomitant radiation boost in neoadjuvant chemoradiotherapy for locally advanced rectal cancer.* Anticancer Research, 2017. **37**(6): p. 3201-3206.

8. Bae, B.K., et al., *Simultaneous integrated boost intensity-modulated radiotherapy versus 3-dimensional conformal radiotherapy in preoperative concurrent chemoradiotherapy for locally advanced rectal cancer.* Radiat Oncol J, 2017. **35**(3): p. 208-216.

9. Bakkal, B.H. and O. Elmas, *Dosimetric comparison of organs at risk in 5 different radiotherapy plans in patients with preoperatively irradiated rectal cancer.* Medicine (Baltimore), 2021. **100**(1): p. e24266.

10. Ballonoff, A., et al., *Preoperative capecitabine and accelerated intensity-modulated radiotherapy in locally advanced rectal cancer: a phase II trial.* Am J Clin Oncol, 2008. **31**(3): p. 264-70.

11. Bassi, M.C., et al., *FDG-PET/CT imaging for staging and target volume delineation in preoperative conformal radiotherapy of rectal cancer.* Int J Radiat Oncol Biol Phys, 2008. **70**(5): p. 1423-6.

12. Bertocchi, E., et al., *A comparative analysis between radiation dose intensification and conventional fractionation in neoadjuvant locally advanced rectal cancer: a monocentric prospective observational study.* La radiologia medica, 2020. **125**(10): p. 990-998.

13. Boeke, S., et al., *Online MR guided dose escalated radiotherapy for organ preservation in distal rectal cancer.* Clin Transl Radiat Oncol, 2022. **37**: p. 153-156.

14. But-Hadzic, J. and V. Velenik, *Preoperative intensity-modulated chemoradiation therapy with simultaneous integrated boost in rectal cancer: 2-year follow-up results of phase II study.* Radiology and Oncology, 2018. **52**(1): p. 23-29.

15. Capirci, C., et al., *The Role of Dual-Time Combined 18-Fluorideoxyglucose Positron Emission Tomography and Computed Tomography in the Staging and Restaging Workup of Locally Advanced Rectal Cancer, Treated With Preoperative Chemoradiation Therapy and Radical Surgery.* International Journal of Radiation Oncology Biology Physics, 2009. **74**(5): p. 1461-1469.

16. Chen, H., et al., *Effect and Safety of Radiation Therapy Boost to Extramesorectal Lymph Nodes in Rectal Cancer.* Practical Radiation Oncology, 2020. **10**(5): p. e372-e377.

17. Chen, M., et al., *Radiation boost for synchronous solitary inguinal lymph node metastasis during neoadjuvant chemoradiotherapy for locally advanced rectal cancer.* Discover Oncology, 2021. **12**(1): p. 59.

18. Chiloiro, G., et al., *THeragnostic utilities for neoplastic DisEases of the rectum by MRI guided radiotherapy (THUNDER 2) phase II trial: interim safety analysis.* Radiat Oncol, 2023. **18**(1): p. 163.

19. Colaco, R.J., et al., *Protons offer reduced bone marrow, small bowel, and urinary bladder exposure for patients receiving neoadjuvant radiotherapy for resectable rectal cancer.* J Gastrointest Oncol, 2014. **5**(1): p. 3-8.

20. De Bari, B., et al., *Neoadjuvant chemoradiotherapy delivered with helical tomotherapy under daily image guidance for rectal cancer patients: efficacy and safety in a large, multi-institutional series.* Journal of Cancer Research and Clinical Oncology, 2019. **145**(4): p. 1075-1084.

21. De Paoli, A., et al., *Capecitabine in combination with preoperative radiation therapy in locally advanced, resectable, rectal cancer: a multicentric phase II study.* Ann Oncol, 2006. **17**(2): p. 246-51.

22. Devlin, L., et al., *The in-silico feasibility of dose escalated, hypofractionated radiotherapy for rectal cancer.* Clin Transl Radiat Oncol, 2022. **36**: p. 24-30.

23. Engels, B., et al., *Preoperative Radiotherapy with a Simultaneous Integrated Boost Compared to Chemoradiotherapy for cT3-4 Rectal Cancer: Long-Term Results of a Multicenter Randomized Study.* Cancers (Basel), 2023. **15**(15).

24. Faria, S., et al., *Phase II trial of short-course radiotherapy followed by delayed surgery for locoregionally advanced rectal cancer.* Colorectal Dis, 2014. **16**(2): p. O66-70.

25. Fiorino, C., et al., *A TCP-based early regression index predicts the pathological response in neo-adjuvant radio-chemotherapy of rectal cancer.* Radiotherapy and Oncology, 2018. **128**(3): p. 564-568.

26. Fleischmann, M., et al., *ACO/ARO/AIO-21 - Capecitabine-based chemoradiotherapy in combination with the IL-1 receptor antagonist anakinra for rectal cancer Patients: A phase I trial of the German rectal cancer study group.* Clinical and Translational Radiation Oncology, 2022. **34**: p. 99-106.

27. Garant, A., et al., *MORPHEUS Phase II-III Study: A Pre-Planned Interim Safety Analysis and Preliminary Results.* Cancers (Basel), 2022. **14**(15).

28. Garcia-Aguilar, J., et al., *Organ Preservation in Patients With Rectal Adenocarcinoma Treated With Total Neoadjuvant Therapy.* J Clin Oncol, 2022. **40**(23): p. 2546-2556.

29. Gasparini, G., et al., *A phase II study of neoadjuvant bevacizumab plus capecitabine and concomitant radiotherapy in patients with locally advanced rectal cancer.* Angiogenesis, 2012. **15**(1): p. 141-50.

30. Geng, J.H., et al., *Preliminary results of simultaneous integrated boost intensity-modulated radiation therapy based neoadjuvant chemoradiotherapy on locally advanced rectal cancer with clinically suspected positive lateral pelvic lymph nodes.* Annals of Translational Medicine, 2021. **9**(3).

31. Gérard, J.P., et al., *Preoperative concurrent chemoradiotherapy in locally advanced rectal cancer with high-dose radiation and oxaliplatin-containing regimen: the Lyon R0-04 phase II trial.* J Clin Oncol, 2003. **21**(6): p. 1119-24.

32. Gerard, J.P., et al., *Neoadjuvant chemoradiotherapy with radiation dose escalation with contact x-ray brachytherapy boost or external beam radiotherapy boost for organ preservation in early cT2-cT3 rectal adenocarcinoma (OPERA): a phase 3, randomised controlled trial.* Lancet Gastroenterol Hepatol, 2023. **8**(4): p. 356-367.

33. Guido, A., et al., *Adaptive Individualized high-dose preoperAtive (AIDA) chemoradiation in high-risk rectal cancer: a phase II trial.* Eur J Nucl Med Mol Imaging, 2023. **50**(2): p. 572-580.

34. Gunther, J.R., et al., *Preoperative radiation dose escalation for rectal cancer using a concomitant boost strategy improves tumor downstaging without increasing toxicity: A matched-pair analysis.* Adv Radiat Oncol, 2017. **2**(3): p. 455-464.

35. Gurdal, N., et al., *Neoadjuvant volumetric modulated arc therapy in rectal cancer and the correlation of pathological response with diffusion-weighted MRI and apoptotic markers.* Tumori, 2018. **104**(4): p. 266-272.

36. Hernando-Requejo, O., et al., *Complete pathological responses in locally advanced rectal cancer after preoperative IMRT and integrated-boost chemoradiation.* Strahlentherapie und Onkologie, 2014. **190**(6): p. 515-520.

37. Hong, Y.S., et al., *Preoperative chemoradiation with irinotecan and capecitabine in patients with locally advanced resectable rectal cancer: Long-term results of a phase II study.* International Journal of Radiation Oncology Biology Physics, 2011. **79**(4): p. 1171-1178.

38. Ho-Pun-Cheung, A., et al., *Cyclin D1 gene G870A polymorphism predicts response to neoadjuvant radiotherapy and prognosis in rectal cancer.* Int J Radiat Oncol Biol Phys, 2007. **68**(4): p. 1094-101.

39. Huang, M.Y., et al., *Helical tomotherapy combined with capecitabine in the preoperative treatment of locally advanced rectal cancer.* BioMed Research International, 2014. **2014**.

40. Ingle, M., et al., *Understanding the Benefit of Magnetic Resonance-guided Adaptive Radiotherapy in Rectal Cancer Patients: a Single-centre Study.* Clinical Oncology, 2023. **35**(2): p. e135-e142.

41. Jabbour, S.K., et al., *Intensity-modulated radiation therapy for rectal carcinoma can reduce treatment breaks and emergency department visits.* Int J Surg Oncol, 2012. **2012**: p. 891067.

42. Jankarashvili, N., et al., *Neoadjuvant volumetric modulated arc radiochemotherapy with a simultaneous integrated boost technique compared to standard chemoradiation for locally advanced rectal cancer.* Turkish Journal of Medical Sciences, 2019. **49**(5): p. 1484-1489.

43. Karaca, S. and K.A.A. Karacam, *A comparison of dosimetric and clinical parameters between different IMRT boost techniques in preoperative rectal cancer.* Journal of B.U.ON., 2021. **26**(4): p. 1231-1238.

44. Kiliç, D., et al., *Is there any impact of PET/CT on radiotherapy planning in rectal cancer patients undergoing preoperative IMRT?* Turk J Med Sci, 2015. **45**(1): p. 129-35.

45. Kim, K.H., et al., *Circumferential resection margin positivity after preoperative chemoradiotherapy based on magnetic resonance imaging for locally advanced rectal cancer: Implication of boost radiotherapy to the involved mesorectal fascia.* Japanese Journal of Clinical Oncology, 2016. **46**(4): p. 316-322.

46. Lee, D.S., et al., *Simultaneous Integrated Boost Volumetric Modulated Arc Therapy for Rectal Cancer: Long-Term Results after Protocol-Based Treatment.* Journal of Oncology, 2022. **2022**.

47. Liu, W.Y., et al., *The safety of an MRI simulation-guided boost after short-course preoperative radiotherapy for unresectable rectal cancer (SUNRISE): interim analysis of a randomized phase II trial.* Radiation Oncology, 2022. **17**(1).

48. Lo Greco, M.C., et al., *Integrated Intensified Chemoradiation in the Setting of Total Neoadjuvant Therapy (TNT) in Patients with Locally Advanced Rectal Cancer: A Retrospective Single-Arm Study on Feasibility and Efficacy.* Cancers, 2023. **15**(3).

49. Luna, R.B. and M.V. De Torres Olombrada, *MARC preoperative rectal cancer treatments vs. 3D conformal radiotherapy. A dose distribution comparative study.* PLoS ONE, 2019. **14**(8).

50. Lupattelli, M., et al., *Preoperative Intensified Chemoradiation with Intensity-Modulated Radiotherapy and Simultaneous Integrated Boost Combined with Capecitabine in Locally Advanced Rectal Cancer: Long-Term Outcomes of a Real-Life Multicenter Study.* Cancers (Basel), 2023. **15**(23).

51. Mazzola, R., et al., *Sequential boost in neoadjuvant irradiation for T3N0-1 rectal cancer: Long-term results from a single-center experience.* Tumori, 2016. **102**(3): p. 316-322.

52. Mohiuddin, M., et al., *Randomized phase II study of neoadjuvant combined-modality chemoradiation for distal rectal cancer: Radiation Therapy Oncology Group Trial 0012.* J Clin Oncol, 2006. **24**(4): p. 650-5.

53. Movsas, B., et al., *Phase II trial of preoperative chemoradiation with a hyperfractionated radiation boost in locally advanced rectal cancer.* Am J Clin Oncol, 2006. **29**(5): p. 435-41.

54. Ngan, S.Y., et al., *Randomized trial of short-course radiotherapy versus long-course chemoradiation comparing rates of local recurrence in patients with T3 rectal cancer: Trans-Tasman Radiation Oncology Group trial 01.04.* J Clin Oncol, 2012. **30**(31): p. 3827-33.

55. O'Neill, B., et al., *Successful downstaging of high rectal and recto-sigmoid cancer by neo-adjuvant chemo-radiotherapy.* Clin Med Oncol, 2008. **2**: p. 135-44.

56. Osti, M.F., et al., *Neoadjuvant chemoradiation with concomitant boost radiotherapy associated to capecitabine in rectal cancer patients.* Int J Colorectal Dis, 2014. **29**(7): p. 835-42.

57. Owens, R., et al., *Intensity-Modulated Radiotherapy With a Simultaneous Integrated Boost in Rectal Cancer.* Clinical Oncology, 2020. **32**(1): p. 35-42.

58. Parekh, A., et al., *Acute gastrointestinal toxicity and tumor response with preoperative intensity modulated radiation therapy for rectal cancer.* Gastrointest Cancer Res, 2013. **6**(5-6): p. 137-43.

59. Park, J.H., et al., *Randomized phase 3 trial comparing preoperative and postoperative chemoradiotherapy with capecitabine for locally advanced rectal cancer.* Cancer, 2011. **117**(16): p. 3703-12.

60. Picardi, V., et al., *Preoperative Chemoradiation With VMAT-SIB in Rectal Cancer: A Phase II Study.* Clinical Colorectal Cancer, 2017. **16**(1): p. 16-22.

61. Radu, C., et al., *Integrated peripheral boost in preoperative radiotherapy for the locally most advanced non-resectable rectal cancer patients.* Acta Oncologica, 2013. **52**(3): p. 528-537.

62. Samuelian, J.M., et al., *Reduced acute bowel toxicity in patients treated with intensity-modulated radiotherapy for rectal cancer.* Int J Radiat Oncol Biol Phys, 2012. **82**(5): p. 1981-7.

63. Schmoll, H.J., et al., *Pre- and Postoperative Capecitabine Without or With Oxaliplatin in Locally Advanced Rectal Cancer: PETACC 6 Trial by EORTC GITCG and ROG, AIO, AGITG, BGDO, and FFCD.* J Clin Oncol, 2021. **39**(1): p. 17-29.

64. Schrag, D., et al., *Preoperative Treatment of Locally Advanced Rectal Cancer.* N Engl J Med, 2023. **389**(4): p. 322-334.

65. Seierstad, T., et al., *MR-guided simultaneous integrated boost in preoperative radiotherapy of locally advanced rectal cancer following neoadjuvant chemotherapy.* Radiotherapy and Oncology, 2009. **93**(2): p. 279-284.

66. Shepherd, M., et al., *Developing knowledge-based planning for gynaecological and rectal cancers: a clinical validation of RapidPlan™.* Journal of Medical Radiation Sciences, 2020. **67**(3): p. 217-224.

67. Spatola, C., et al., *Intensified neoadjuvant radio-chemotherapy for locally advanced rectal cancer: mono-istitutional experience and long-term results.* Int-J-Radiat-Res, 2019. **17**(2): p. 265-273.

68. Temelli, O., et al., *Integral dose and dosimetric comparison of neoadjuvant simultaneous integrated boost (SIB) radiotherapy technique for rectal cancer using intensity-modulated radiotherapy (IMRT), volumetric modulated arc therapy (VMAT), and helical tomotherapy (HT).* UHOD - Uluslararasi Hematoloji-Onkoloji Dergisi, 2019. **29**(3): p. 147-156.

69. Tepper, J.E., et al., *Adjuvant therapy in rectal cancer: analysis of stage, sex, and local control--final report of intergroup 0114.* J Clin Oncol, 2002. **20**(7): p. 1744-50.

70. Tey, J., et al., *A phase II trial of preoperative concurrent chemotherapy and dose escalated intensity modulated radiotherapy (IMRT) for locally advanced rectal cancer.* J Cancer, 2017. **8**(16): p. 3114-3121.

71. Tunio, M.A., et al., *High-dose-rate intraluminal brachytherapy during preoperative chemoradiation for locally advanced rectal cancers.* World Journal of Gastroenterology, 2010. **16**(35): p. 4436-4442.

72. Verweij, M.E., et al., *Towards Response ADAptive Radiotherapy for organ preservation for intermediate-risk rectal cancer (preRADAR): protocol of a phase I dose-escalation trial.* BMJ Open, 2023. **13**(6): p. e065010.

73. Vestermark, L.W., H.A. Jensen, and P. Pfeiffer, *High-dose radiotherapy (60 Gy) with oral UFT/folinic acid and escalating doses of oxaliplatin in patients with non-resectable locally advanced rectal cancer (LARC): A phase i trial.* Acta Oncologica, 2012. **51**(3): p. 311-317.

74. Wawok, P., et al., *Preoperative radiotherapy and local excision of rectal cancer: Long-term results of a randomised study.* Radiotherapy and Oncology, 2018. **127**(3): p. 396-403.

75. Whaley, J.T., et al., *Clinical utility of integrated positron emission tomography/computed tomography imaging in the clinical management and radiation treatment planning of locally advanced rectal cancer.* Practical Radiation Oncology, 2014. **4**(4): p. 226-232.

76. Xu, W., et al., *Effect of concurrent radiotherapy and simultaneous oral capecitabine chemotherapy on locally advanced middle and lower rectal cancer.* International Journal of Clinical and Experimental Medicine, 2016. **9**(2): p. 3614-3620.

77. Yang, Y., et al., *A dosimetric analysis of preoperative intensity-modulated and image-guided radiation therapy with and without simultaneous integrated boost for locally advanced rectal cancer.* Technology in Cancer Research and Treatment, 2015. **14**(5): p. 557-563.

78. Yang, D.S., et al., *Sequential simulation computed tomography allows assessment of internal rectal movements during preoperative chemoradiotherapy in rectal cancer.* J Cancer Res Ther, 2019. **15**(1): p. 1-8.

79. Yang, Y., et al., *Preoperative volumetric modulated arc therapy with simultaneous integrated boost for locally advanced distal rectal cancer.* Technology in Cancer Research and Treatment, 2019. **18**.

80. Zhang, M.X., et al., *Dose escalation of preoperative short-course radiotherapy followed by neoadjuvant chemotherapy in locally advanced rectal cancer: Protocol for an open-label, single-centre, phase i clinical trial.* BMJ Open, 2019. **9**(3).

81. Zhao, J., et al., *Dosimetric comparisons of VMAT, IMRT and 3DCRT for locally advanced rectal cancer with simultaneous integrated boost.* Oncotarget, 2016. **7**(5): p. 6345-51.

82. Zhao, J., et al., *Concomitant dose escalation with image-guided tomotherapy in locally advanced mid–low rectal cancer: A single-center study.* Cancer Management and Research, 2019. **11**: p. 1579-1586.

83. Zhao, M., et al., *Apparent diffusion coefficient for the prediction of tumor response to neoadjuvant chemo-radiotherapy in locally advanced rectal cancer.* Radiat Oncol, 2021. **16**(1): p. 17.

84. Allal, A.S., et al., *Preoperative hyperfractionated radiotherapy for locally advanced rectal cancers: a phase I-II trial.* Int J Radiat Oncol Biol Phys, 2002. **54**(4): p. 1076-81.

85. Freedman, G.M., et al., *Phase I trial of preoperative hypofractionated intensity-modulated radiotherapy with incorporated boost and oral capecitabine in locally advanced rectal cancer.* Int J Radiat Oncol Biol Phys, 2007. **67**(5): p. 1389-93.

86. Mohiuddin, M., et al., *Neoadjuvant chemoradiation for distal rectal cancer: 5-year updated results of a randomized phase 2 study of neoadjuvant combined modality chemoradiation for distal rectal cancer.* Int J Radiat Oncol Biol Phys, 2013. **86**(3): p. 523-8.

87. Avancha, R. and V.S.R. Rakesh Kumar, *Surgical Outcomes in Carcinoma Rectum after Radical Radiation.* European Journal of Molecular and Clinical Medicine, 2022. **9**(5): p. 472-479.

88. Allal, A.S., et al., *Preoperative concomitant hyperfractionated radiotherapy and gemcitabine for locally advanced rectal cancers: A phase I-II trial.* Cancer Journal, 2005. **11**(2): p. 133-139.

89. Gani, C., et al., *A novel approach for radiotherapy dose escalation in rectal cancer using online MR-guidance and rectal ultrasound gel filling - Rationale and first in human.* Radiother Oncol, 2021. **164**: p. 37-42.

90. Musio, D., et al., *Comparison between intensified neoadjuvant treatment and standard preoperative chemoradiation for rectal cancer.* Tumori, 2010. **96**(1): p. 11-6.

91. Avci, G.G. and I.P. Aral, *The role of MRI and 18F-FDG PET/CT with respect to evaluation of pathological response in the rectal cancer patients after neoadjuvant chemoradiotherapy.* Indian J Cancer, 2021.

92. Alongi, F., et al., *Radiation dose intensification in pre-operative chemo-radiotherapy for locally advanced rectal cancer.* Clin Transl Oncol, 2017. **19**(2): p. 189-196.

93. Garcia-Aguilar, J., et al., *Organ preservation for clinical T2N0 distal rectal cancer using neoadjuvant chemoradiotherapy and local excision (ACOSOG Z6041): results of an open-label, single-arm, multi-institutional, phase 2 trial.* Lancet Oncol, 2015. **16**(15): p. 1537-1546.

94. Myerson, R.J., et al., *A phase I/II trial of three-dimensionally planned concurrent boost radiotherapy and protracted venous infusion of 5-FU chemotherapy for locally advanced rectal carcinoma.* International Journal of Radiation Oncology Biology Physics, 2001. **50**(5): p. 1299-1308.

95. Bitterman, D.S., et al., *Predictors of Complete Response and Disease Recurrence Following Chemoradiation for Rectal Cancer.* Front Oncol, 2015. **5**: p. 286.

96. Alvarez, J., et al., *ALLIANCE A022104/NRG-GI010: The Janus Rectal Cancer Trial: a randomized phase II/III trial testing the efficacy of triplet versus doublet chemotherapy regarding clinical complete response and disease-free survival in patients with locally advanced rectal cancer*. 2024.

97. Garcia-Aguilar, J., et al., *Effect of adding mFOLFOX6 after neoadjuvant chemoradiation in locally advanced rectal cancer: A multicentre, phase 2 trial.* The Lancet Oncology, 2015. **16**(8): p. 957-966.

98. Myerson, R., et al., *Early results from a phase I/II radiation dose-escalation study with concurrent amifostine and infusional 5-fluorouracil chemotherapy for preoperative treatment of unresectable or locally recurrent rectal carcinoma.* Semin Oncol, 2002. **29**(6 Suppl 19): p. 29-33.

99. Bountouroglou, N., et al., *The RACOX phase I study: Radiation (RA), capecitabine (C) and oxaliplatin (OX) as adjuvant treatment of stage II and III rectal cancer.* Journal of B.U.ON., 2004. **9**(4): p. 383-390.

100. Azria, D., et al., *Prognostic impact of epidermal growth factor receptor (EGFR) expression on loco-regional recurrence after preoperative radiotherapy in rectal cancer.* BMC cancer, 2005. **5**.

101. Gerard, J.P., et al., *Long-term control of T2-T3 rectal adenocarcinoma with radiotherapy alone.* Int J Radiat Oncol Biol Phys, 2002. **54**(1): p. 142-9.

102. Park, H.C., et al., *Temporal Patterns of Fatigue Predict Pathologic Response in Patients Treated With Preoperative Chemoradiation Therapy for Rectal Cancer.* International Journal of Radiation Oncology Biology Physics, 2009. **75**(3): p. 775-781.

103. Bruera, G., et al., *Dose-finding study of oxaliplatin associated to capecitabine-based preoperative chemoradiotherapy in locally advanced rectal cancer.* Oncotarget, 2018. **9**(25): p. 17906-17914.

104. Bazarbashi, S., et al., *Pre-operative chemoradiotherapy using capecitabine and cetuximab followed by definitive surgery in patients with operable rectal cancer.* Hematol Oncol Stem Cell Ther, 2016. **9**(4): p. 147-153.

105. Gerard, J.P., et al., *Organ preservation in rectal adenocarcinoma (T1) T2-T3 Nx M0. Historical overview of the Lyon Sud - nice experience using contact x-ray brachytherapy and external beam radiotherapy for 120 patients.* Acta Oncol, 2015. **54**(4): p. 545-51.

106. Passoni, P., et al., *Feasibility of an adaptive strategy in preoperative radiochemotherapy for rectal cancer with image-guided tomotherapy: Boosting the dose to the shrinking tumor.* International Journal of Radiation Oncology Biology Physics, 2013. **87**(1): p. 67-72.

107. Bujko, K., et al., *Preoperative radiotherapy and local excision of rectal cancer with immediate radical re-operation for poor responders: A prospective multicentre study.* Radiotherapy and Oncology, 2013. **106**(2): p. 198-205.

108. Bird, D., et al., *The benefit of MR-only radiotherapy treatment planning for anal and rectal cancers: A planning study.* J Appl Clin Med Phys, 2021. **22**(11): p. 41-53.

109. Gevaert, T., et al., *Implementation of HybridArc treatment technique in preoperative radiotherapy of rectal cancer: dose patterns in target lesions and organs at risk as compared to helical Tomotherapy and RapidArc.* Radiation Oncology, 2012. **7**(1).

110. Pfeiffer, P., *High-dose radiotherapy and concurrent UFT plus 1-leucovorin in locally advanced rectal cancer: A phase I trial.* Acta Oncologica, 2005. **44**(3): p. 224-229.

111. Chan, A.K., et al., *Preoperative chemotherapy and pelvic radiation for tethered or fixed rectal cancer: a phase II dose escalation study.* Int J Radiat Oncol Biol Phys, 2000. **48**(3): p. 843-56.

112. Boldrini, L., et al., *Hybrid Tri-Co-60 MRI radiotherapy for locally advanced rectal cancer: An in silico evaluation.* Technical Innovations and Patient Support in Radiation Oncology, 2018. **6**: p. 5-10.

113. Guillem, J.G., et al., *A prospective pathologic analysis using whole-mount sections of rectal cancer following preoperative combined modality therapy: implications for sphincter preservation.* Ann Surg, 2007. **245**(1): p. 88-93.

114. Picardi, V., et al., *Concurrent chemoradiation with concomitant boost in locally advanced rectal cancer: A phase II study.* Anticancer Research, 2016. **36**(8): p. 4081-4087.

115. Conroy, T., et al., *Neoadjuvant chemotherapy with FOLFIRINOX and preoperative chemoradiotherapy for patients with locally advanced rectal cancer (UNICANCER-PRODIGE 23): a multicentre, randomised, open-label, phase 3 trial.* Lancet Oncol, 2021. **22**(5): p. 702-715.

116. Bonnen, M., et al., *Long-term results using local excision after preoperative chemoradiation among selected T3 rectal cancer patients.* Int J Radiat Oncol Biol Phys, 2004. **60**(4): p. 1098-105.

117. Habr-Gama, A., *Assessment and management of the complete clinical response of rectal cancer to chemoradiotherapy.* Colorectal Dis, 2006. **8 Suppl 3**: p. 21-4.

118. Pietsch, A.P., et al., *Effect of neoadjuvant chemoradiation on postoperative fecal continence and anal sphincter function in rectal cancer patients.* International Journal of Colorectal Disease, 2007. **22**(11): p. 1311-1317.

119. D'Amata, G., et al., *The “Watch and wait” approach following chemoradiotherapy for rectal cancer: a case series and review of literature.* Ann Ital Chir, 2021. **92**(5): p. 531-538.

120. Bonomo, P., et al., *1.5 T MR-linac planning study to compare two different strategies of rectal boost irradiation.* Clin Transl Radiat Oncol, 2021. **26**: p. 86-91.

121. Habr-Gama, A., et al., *Increasing the rates of complete response to neoadjuvant chemoradiotherapy for distal rectal cancer: results of a prospective study using additional chemotherapy during the resting period.* Dis Colon Rectum, 2009. **52**(12): p. 1927-34.

122. Raso, R., et al., *Assessment and clinical validation of margins for adaptive simultaneous integrated boost in neo-adjuvant radiochemotherapy for rectal cancer.* Phys Med, 2015. **31**(2): p. 167-72.

123. Datta, D., et al., *Non-operative management in low-lying rectal cancers undergoing chemoradiation.* Journal of Cancer Research and Therapeutics, 2024. **20**(1): p. 417-422.

124. Braendengen, M., et al., *Randomized phase III study comparing preoperative radiotherapy with chemoradiotherapy in nonresectable rectal cancer.* J Clin Oncol, 2008. **26**(22): p. 3687-94.

125. Habr-Gama, A., et al., *Local recurrence after complete clinical response and watch and wait in rectal cancer after neoadjuvant chemoradiation: impact of salvage therapy on local disease control.* Int J Radiat Oncol Biol Phys, 2014. **88**(4): p. 822-8.

126. Roh, M.S., et al., *Preoperative multimodality therapy improves disease-free survival in patients with carcinoma of the rectum: NSABP R-03.* J Clin Oncol, 2009. **27**(31): p. 5124-30.

127. De Las Heras, M., et al., *Multicenter phase II clinical trial of preoperative capecitabine with concurrent radiotherapy in patients with locally advanced rectal cancer.* Clinical and Translational Oncology, 2013. **15**(4): p. 294-299.

128. Burbach, J.P., et al., *RandomizEd controlled trial for pre-operAtive dose-escaLation BOOST in locally advanced rectal cancer (RECTAL BOOST study): study protocol for a randomized controlled trial.* Trials, 2015. **16**: p. 58.

129. Habr-Gama, A., et al., *Organ Preservation in cT2N0 Rectal Cancer After Neoadjuvant Chemoradiation Therapy: The Impact of Radiation Therapy Dose-escalation and Consolidation Chemotherapy.* Annals of Surgery, 2019. **269**(1).

130. Rouanet, P., et al., *Restorative and nonrestorative surgery for low rectal cancer after high-dose radiation: long-term oncologic and functional results.* Dis Colon Rectum, 2002. **45**(3): p. 305-13; discussion 313-5.

131. Di Tommaso, M., et al., *Treatment Intensification for Locally Advanced Rectal Cancer: Impact on Pathological Complete Response and Outcomes.* In Vivo, 2020. **34**(3): p. 1223.

132. Burbach, J.P.M., et al., *Inter-observer agreement of MRI-based tumor delineation for preoperative radiotherapy boost in locally advanced rectal cancer.* Radiotherapy and Oncology, 2016. **118**(2): p. 399-407.

133. Hong, T.S., et al., *NRG oncology radiation therapy oncology group 0822: A phase 2 study of preoperative chemoradiation therapy using intensity modulated radiation therapy in combination with capecitabine and oxaliplatin for patients with locally advanced rectal cancer.* International Journal of Radiation Oncology Biology Physics, 2015. **93**(1): p. 29-36.

134. Sanfilippo, N.J., et al., *T4 rectal cancer treated with preoperative chemoradiation to the posterior pelvis followed by multivisceral resection: Patterns of failure and limitations of treatment.* International Journal of Radiation Oncology Biology Physics, 2001. **51**(1): p. 176-183.

135. Dumont, F., et al., *Model predicting the ypN0 status after good response to chemoradiotherapy in rectal cancer.* American Journal of Surgery, 2018. **216**(3): p. 438-443.

136. Capirci, C., et al., *Concurrent boost radiotherapy as preoperative treatment for locally advanced rectal carcinoma: A new beam arrangement.* Tumori, 2002. **88**(4): p. 325-330.

137. Ingle, M., et al., *Quantitative analysis of diffusion weighted imaging in rectal cancer during radiotherapy using a magnetic resonance imaging integrated linear accelerator.* Physics and Imaging in Radiation Oncology, 2022. **23**: p. 32-37.

138. São Julião, G.P., et al., *Is neoadjuvant chemoradiation with dose-escalation and consolidation chemotherapy sufficient to increase surgery-free and distant metastases-free survival in baseline cT3 rectal cancer?* European Journal of Surgical Oncology, 2018. **44**(1): p. 93-99.

139. Garcia-Aguilar, J., et al., *Optimal timing of surgery after chemoradiation for advanced rectal cancer: preliminary results of a multicenter, nonrandomized phase II prospective trial.* Ann Surg, 2011. **254**(1): p. 97-102.

140. Caravatta, L., et al., *Concomitant boost radiotherapy and multidrug chemotherapy in the neoadjuvant treatment of locally advanced rectal cancer: Results of a phase II study.* Acta Oncologica, 2011. **50**(8): p. 1151-1157.

141. Janjan, N.A., et al., *Prospective trial of preoperative concomitant boost radiotherapy with continuous infusion 5-fluorouracil for locally advanced rectal cancer.* International Journal of Radiation Oncology Biology Physics, 2000. **47**(3): p. 713-718.

142. Tuta, M., et al., *Total neoadjuvant treatment of locally advanced rectal cancer with high risk factors in Slovenia.* Radiology and Oncology, 2019.

143. Habr-Gama, A., et al., *Watch and wait approach following extended neoadjuvant chemoradiation for distal rectal cancer: are we getting closer to anal cancer management?* Dis Colon Rectum, 2013. **56**(10): p. 1109-17.

144. Caravatta, L., et al., *Neoadjuvant accelerated concomitant boost radiotherapy and multidrug chemotherapy in locally advanced rectal cancer: A dose-escalation study.* American Journal of Clinical Oncology: Cancer Clinical Trials, 2012. **35**(5): p. 424-431.

145. Jeong, J.H., et al., *Phase 1 Study of Preoperative Chemoradiation Therapy With Temozolomide and Capecitabine in Patients With Locally Advanced Rectal Cancer.* International Journal of Radiation Oncology Biology Physics, 2016. **96**(2): p. 289-295.

146. Urbano, M.T.G., et al., *Intensity-modulated radiotherapy in patients with locally advanced rectal cancer reduces volume of bowel treated to high dose levels.* International Journal of Radiation Oncology Biology Physics, 2006. **65**(3): p. 907-916.

147. Hashimoto, T., et al., *Total neoadjuvant therapy followed by a watch-and-wait strategy for patients with rectal cancer (TOWARd): protocol for single-arm phase II/III confirmatory trial (JCOG2010).* BJS Open, 2023. **7**(6).

148. Chao, M., et al., *Preoperative chemotherapy and radiotherapy for locally advanced rectal cancer.* ANZ Journal of Surgery, 2005. **75**(5): p. 286-291.

149. Jin, C.K., et al., *Preoperative concurrent radiotherapy with capecitabine before total mesorectal excision in locally advanced rectal cancer.* International Journal of Radiation Oncology Biology Physics, 2005. **63**(2): p. 346-353.

150. Valentini, V., et al., *Preoperative chemoradiation with raltitrexed ('Tomudex') for T2/N+ and T3/N+ rectal cancers: A phase I study.* European Journal of Cancer, 2001. **37**(16): p. 2050-2055.

151. Hudson, E.M., et al., *A Phase II trial of Higher RadiOtherapy Dose in the Eradication of early rectal cancer (APHRODITE): Protocol for a multicentre, open-label randomised controlled trial.* BMJ Open, 2022. **12**(4).

152. Chau, I., et al., *Neoadjuvant systemic fluorouracil and mitomycin C prior to synchronous chemoradiation is an effective strategy in locally advanced rectal cancer.* British Journal of Cancer, 2003. **88**(7): p. 1017-1024.

153. Kim, D.Y., et al., *Preoperative chemoradiotherapy with concomitant small field boost irradiation for locally advanced rectal cancer: a multi-institutional phase II study (KROG 04-01).* Dis Colon Rectum, 2006. **49**(11): p. 1684-91.

154. Valentini, V., et al., *Infusional 5-Fluorouracil and ZD1839 (Gefitinib-Iressa) in Combination With Preoperative Radiotherapy in Patients With Locally Advanced Rectal Cancer: A Phase I and II Trial (1839IL/0092).* International Journal of Radiation Oncology Biology Physics, 2008. **72**(3): p. 644-649.

155. Kennecke, H., et al., *Pre-operative bevacizumab, capecitabine, oxaliplatin and radiation among patients with locally advanced or low rectal cancer: A phase II trial.* European Journal of Cancer, 2012. **48**(1): p. 37-45.

156. Chiloiro, G., et al., *MR-guided radiotherapy in rectal cancer: First clinical experience of an innovative technology.* Clinical and Translational Radiation Oncology, 2019. **18**: p. 80-86.

157. Kim, D.Y., et al., *Comparison of 5-fluorouracil/leucovorin and capecitabine in preoperative chemoradiotherapy for locally advanced rectal cancer.* Int J Radiat Oncol Biol Phys, 2007. **67**(2): p. 378-84.

158. Valentini, V., et al., *The INTERACT Trial: Long-term results of a randomised trial on preoperative capecitabine-based radiochemotherapy intensified by concomitant boost or oxaliplatin, for cT2 (distal)-cT3 rectal cancer.* Radiother Oncol, 2019. **134**: p. 110-118.

159. Kim, J.S., et al., *Preoperative chemoradiation using oral capecitabine in locally advanced rectal cancer.* International Journal of Radiation Oncology Biology Physics, 2002. **54**(2): p. 403-408.

160. Chiloiro, G., et al., *THUNDER 2: THeragnostic Utilities for Neoplastic DisEases of the Rectum by MRI guided radiotherapy.* BMC Cancer, 2022. **22**(1): p. 67.

161. Klautke, G., et al., *Intensified concurrent chemoradiotherapy with 5-fluorouracil and irinotecan as neoadjuvant treatment in patients with locally advanced rectal cancer.* British Journal of Cancer, 2005. **92**(7): p. 1215-1220.

162. Velenik, V., et al., *Influence of concurrent capecitabine based chemoradiotherapy with bevacizumab on the survival rate, late toxicity and health-related quality of life in locally advanced rectal cancer: a prospective phase II CRAB trial.* Radiol Oncol, 2020. **54**(4): p. 461-469.

163. Klautke, G., et al., *Intensified irinotecan-based neoadjuvant chemoradiotherapy in rectal cancer: four consecutive designed studies to minimize acute toxicity and to optimize efficacy measured by pathologic complete response.* Radiother Oncol, 2007. **85**(3): p. 379-84.

164. Cilla, S., et al., *Volumetric Modulated Arc Therapy with Simultaneous Integrated Boost for Locally Advanced Rectal Cancer.* Clinical Oncology, 2012. **24**(4): p. 261-268.

165. Kleijnen, J.J.E., et al., *MRI-based tumor inter-fraction motion statistics for rectal cancer boost radiotherapy.* Acta Oncol, 2019. **58**(2): p. 232-236.

166. Verweij, M.E., et al., *Impact of Dose-Escalated Chemoradiation on Quality of Life in Patients With Locally Advanced Rectal Cancer: 2-Year Follow-Up of the Randomized RECTAL-BOOST Trial.* Int J Radiat Oncol Biol Phys, 2022. **112**(3): p. 694-703.

167. Markovina, S., et al., *Improved Metastasis- and Disease-Free Survival With Preoperative Sequential Short-Course Radiation Therapy and FOLFOX Chemotherapy for Rectal Cancer Compared With Neoadjuvant Long-Course Chemoradiotherapy: Results of a Matched Pair Analysis.* Int J Radiat Oncol Biol Phys, 2017. **99**(2): p. 417-426.

168. Cilla, S., et al., *Assessing the feasibility of volumetric-modulated arc therapy using simultaneous integrated boost (SIB-VMAT): An analysis for complex head-neck, high-risk prostate and rectal cancer cases.* Medical Dosimetry, 2014. **39**(1): p. 108-116.

169. Krishnan, S., et al., *Phase II study of capecitabine (Xeloda®) and concomitant boost radiotherapy in patients with locally advanced rectal cancer.* International Journal of Radiation Oncology*Biology*Physics, 2006. **66**(3): p. 762-771.

170. Vestermark, L.W., et al., *Long-term results of a phase II trial of high-dose radiotherapy (60 Gy) and UFT/l-leucovorin in patients with non-resectable locally advanced rectal cancer (LARC).* Acta Oncologica, 2008. **47**(3): p. 428-433.

171. Mehta, V.K., et al., *Radiotherapy, concomitant protracted-venous-infusion 5-fluorouracil, and surgery for ultrasound-staged T3 or T4 rectal cancer.* Dis Colon Rectum, 2001. **44**(1): p. 52-8.

172. Couwenberg, A.M., et al., *Efficacy of Dose-Escalated Chemoradiation on Complete Tumor Response in Patients with Locally Advanced Rectal Cancer (RECTAL-BOOST): A Phase 2 Randomized Controlled Trial.* International Journal of Radiation Oncology*Biology*Physics, 2020. **108**(4): p. 1008-1018.

173. Lee, J.H., et al., *Long-term follow-up of preoperative pelvic radiation therapy and concomitant boost irradiation in locally advanced rectal cancer patients: a multi-institutional phase II study (KROG 04-01).* Int J Radiat Oncol Biol Phys, 2012. **84**(4): p. 955-61.

174. Wang, J., et al., *Long-course neoadjuvant chemoradiotherapy with versus without a concomitant boost in locally advanced rectal cancer: A randomized, multicenter, phase II trial (FDRT-002).* Radiation Oncology, 2019. **14**(1).

175. O'Connell, M.J., et al., *Capecitabine and oxaliplatin in the preoperative multimodality treatment of rectal cancer: Surgical end points from national surgical adjuvant breast and bowel project trial R-04.* Journal of Clinical Oncology, 2014. **32**(18): p. 1927-1934.

176. Cubillo, A., et al., *A prospective pilot study of target-guided personalized chemotherapy with intensity-modulated radiotherapy in patients with early rectal cancer.* Am J Clin Oncol, 2014. **37**(2): p. 117-21.

177. Lee, J.A., et al., *Tumor volume reduction assessed by planning computed tomography in patients with rectal cancer during preoperative chemoradiation: impact of residual tumor volume on the prediction of pathologic tumor regression.* Tumori, 2014. **100**(2): p. 158-62.

178. White, I., et al., *Interobserver variability in target volume delineation for CT/MRI simulation and MRI-guided adaptive radiotherapy in rectal cancer.* Br J Radiol, 2021. **94**(1128): p. 20210350.

179. Ofshteyn, A., et al., *Adding Boost to Standard Neoadjuvant Radiation for Rectal Cancer Improves Likelihood of Complete Response.* J Gastrointest Surg, 2020. **24**(7): p. 1655-1662.

180. Czito, B.G., et al., *A Phase I trial of preoperative eniluracil plus 5-fluorouracil and radiation for locally advanced or unresectable adenocarcinoma of the rectum and colon.* Int J Radiat Oncol Biol Phys, 2004. **58**(3): p. 779-85.

181. Li, J.L., et al., *Preoperative concomitant boost intensity-modulated radiotherapy with oral capecitabine in locally advanced mid-low rectal cancer: A phase II trial.* Radiotherapy and Oncology, 2012. **102**(1): p. 4-9.

182. Xu, Q., et al., *Quantifying rigid and nonrigid motion of liver tumors during stereotactic body radiation therapy.* Int J Radiat Oncol Biol Phys, 2014. **90**(1): p. 94-101.

183. Paszt, A., et al., *Clinical benefits of oral capecitabine over intravenous 5-fluorouracyl regimen in case of neoadjuvant chemoradiotherapy followed by surgery for locally advanced rectal cancer.* Pathology and Oncology Research, 2022. **28**.

184. De Ridder, M., et al., *Phase II Study of Preoperative Helical Tomotherapy for Rectal Cancer.* International Journal of Radiation Oncology Biology Physics, 2008. **70**(3): p. 728-734.

185. Li, S., et al., *Effect of simultaneous integrated boost intensity modulated radiation therapy (Sib-imrt) and non-operative strategy on outcomes of distal rectal cancer patients with clinically positive lateral pelvic lymph node.* Cancer Management and Research, 2021. **13**: p. 537-546.

186. Xu, L., et al., *Prognostic significance of tumour regression grade after neoadjuvant chemoradiotherapy for a cohort of patients with locally advanced rectal cancer: an 8-year retrospective single-institutional study.* Colorectal Dis, 2017. **19**(7): p. O263-o271.

187. Rödel, C., et al., *Extensive surgery after high-dose preoperative chemoradiotherapy for locally advanced recurrent rectal cancer.* Dis Colon Rectum, 2000. **43**(3): p. 312-9.

188. Dewdney, A., et al., *Multicenter randomized phase II clinical trial comparing neoadjuvant oxaliplatin, capecitabine, and preoperative radiotherapy with or without cetuximab followed by total mesorectal excision in patients with high-risk rectal cancer (EXPERT-C).* J Clin Oncol, 2012. **30**(14): p. 1620-7.

189. Loi, S., et al., *Oxaliplatin combined with infusional 5-fluorouracil and concomitant radiotherapy in inoperable and metastatic rectal cancer: A phase I trial.* British Journal of Cancer, 2005. **92**(4): p. 655-661.

190. Yang, T.J., et al., *Clinical and dosimetric predictors of acute hematologic toxicity in rectal cancer patients undergoing chemoradiotherapy.* Radiother Oncol, 2014. **113**(1): p. 29-34.

191. Schou, J.V., et al., *Induction chemotherapy with capecitabine and oxaliplatin followed by chemoradiotherapy before total mesorectal excision in patients with locally advanced rectal cancer.* Ann Oncol, 2012. **23**(10): p. 2627-2633.

192. Eijkelenkamp, H., et al., *Planning target volume margin assessment for online adaptive MR-guided dose-escalation in rectal cancer on a 1.5 T MR-Linac.* Radiotherapy and Oncology, 2021. **162**: p. 150-155.

193. Lupattelli, M., et al., *Oxaliplatin with raltitrexed and preoperative radiotherapy in T3-T4 extraperitoneal rectal cancer. A dose finding study.* Tumori, 2006. **92**(6): p. 474-480.

194. Zhu, J., et al., *Phase II trial of first-line chemoradiotherapy with intensity-modulated radiation therapy followed by chemotherapy for synchronous unresectable distant metastases rectal adenocarcinoma.* Radiation Oncology, 2013. **8**(1).

195. Singh, K., et al., *A prospective randomized trial comparing capecitabine-based chemoradiotherapy with 5-FU-based chemoradiotherapy in neoadjuvant setting in locally advanced carcinoma rectum.* Indian J Cancer, 2017. **54**(1): p. 347-351.

196. Elashwah, A., et al., *Retrospective Evaluation of the Impact of Dose Escalation Using Pre-operative Simultaneous Integrated Boost Volumetric Modulated Arc Therapy on the Outcome of Locally Advanced Rectal Cancer Patients.* Journal of Gastrointestinal Cancer, 2023. **54**(3): p. 927-936.

197. Lupattelli, M., et al., *Preoperative intensity-modulated radiotherapy with a simultaneous integrated boost combined with Capecitabine in locally advanced rectal cancer: Short-term results of a multicentric study.* Radiation Oncology, 2017. **12**(1).

198. Zhu, J., et al., *Concomitant boost IMRT-based neoadjuvant chemoradiotherapy for clinical stage II/III rectal adenocarcinoma: Results of a phase II study.* Radiation Oncology, 2014. **9**(1).

199. Sirák, I., et al., *The Time Between Chemoradiation and Surgery for Rectal Carcinoma Negatively Influences Mesorectal Excision Quality.* Pathology & Oncology Research, 2020. **26**(3): p. 1565-1572.

200. Engels, B., et al., *Phase II study of preoperative helical tomotherapy with a simultaneous integrated boost for rectal cancer.* Int J Radiat Oncol Biol Phys, 2012. **83**(1): p. 142-8.

201. Meyer, J., et al., *A Phase I Clinical Trial of the Phosphatidylserine-targeting Antibody Bavituximab in Combination With Radiation Therapy and Capecitabine in the Preoperative Treatment of Rectal Adenocarcinoma.* Am J Clin Oncol, 2018. **41**(10): p. 972-976.

202. Wygoda, M., et al., *Preoperative radiotherapy and concurrent chemotherapy with bolus 5-fluorouracil for rectal cancer: A prospective analysis of 98 patients.* Tumori, 2010. **96**(5): p. 709-712.

203. Engineer, R., et al., *Escalated radiation dose alone vs. concurrent chemoradiation for locally advanced and unresectable rectal cancers: Results from phase II randomized study.* International Journal of Colorectal Disease, 2013. **28**(7): p. 959-966.

204. Mohiuddin, M., et al., *Prognostic significance of postchemoradiation stage following preoperative chemotherapy and radiation for advanced/recurrent rectal cancers.* International Journal of Radiation Oncology Biology Physics, 2000. **48**(4): p. 1075-1080.

205. Yamashita, H., et al., *Comparison of volumetric-modulated arc therapy using simultaneous integrated boosts (SIB-VMAT) of 45 Gy/55 Gy in 25 fractions with conventional radiotherapy in preoperative chemoradiation for rectal cancers: A propensity score case-matched analysis.* Radiation Oncology, 2017. **12**(1).

206. Everaert, H., et al., *Prediction of response to neoadjuvant radiotherapy in patients with locally advanced rectal cancer by means of sequential 18FDG-PET.* Int J Radiat Oncol Biol Phys, 2011. **80**(1): p. 91-6.

207. Mohiuddin, M., et al., *Preoperative chemoradiation in fixed distal rectal cancer: dose time factors for pathological complete response.* Int J Radiat Oncol Biol Phys, 2000. **46**(4): p. 883-8.

208. Yang, R., et al., *Laparoscopic surgery after neoadjuvant therapy in elderly patients with rectal cancer.* Journal of B.U.ON., 2017. **22**(4): p. 869-874.

209. Xu, B.H., et al., *Pilot study of intense neoadjuvant chemoradiotherapy for locally advanced rectal cancer: retrospective review of a phase II study.* Tumori, 2014. **100**(2): p. 149-57.
